# Sequence-defined positioning of amine and amide residues to control catechol driven wet adhesion†

**DOI:** 10.1039/d0sc03457f

**Published:** 2020-08-31

**Authors:** Lukas Fischer, Alexander K. Strzelczyk, Nils Wedler, Christian Kropf, Stephan Schmidt, Laura Hartmann

**Affiliations:** Institut für Organische und Makromolekulare Chemie, Heinrich-Heine-Universität Düsseldorf Universitätsstr. 1 40225 Düsseldorf Germany stephan.schmidt@hhu.de laura.hartmann@hhu.de; Laundry & Home Care, Henkel AG & Co. KGaA Henkelstr. 67 40589 Düsseldorf Germany

## Abstract

Catechol and amine residues, both abundantly present in mussel adhesion proteins, are known to act cooperatively by displacing hydration barriers before binding to mineral surfaces. In spite of synthetic efforts toward mussel-inspired adhesives, the effect of positioning of the involved functional groups along a polymer chain is not well understood. By using sequence-defined oligomers grafted to soft hydrogel particles as adhesion probes, we study the effect of catechol–amine spacing, as well as positioning relative to the oligomer terminus. We demonstrate that the catechol–amine spacing has a significant effect on adhesion, while shifting their position has a small effect. Notably, combinations of non-charged amides and catechols can achieve similar cooperative effects on adhesion when compared to amine and catechol residues. Thus, these findings provide a blueprint for the design of next generation mussel-inspired adhesives.

## Abstract 2

The catechol driven adhesion of precision macromolecules on glass surfaces is quantified by soft colloidal probe readout. Catechol moieties are shown to synergize with amine and amide residues depending on residue spacing and residue order.

## Graphical abstract

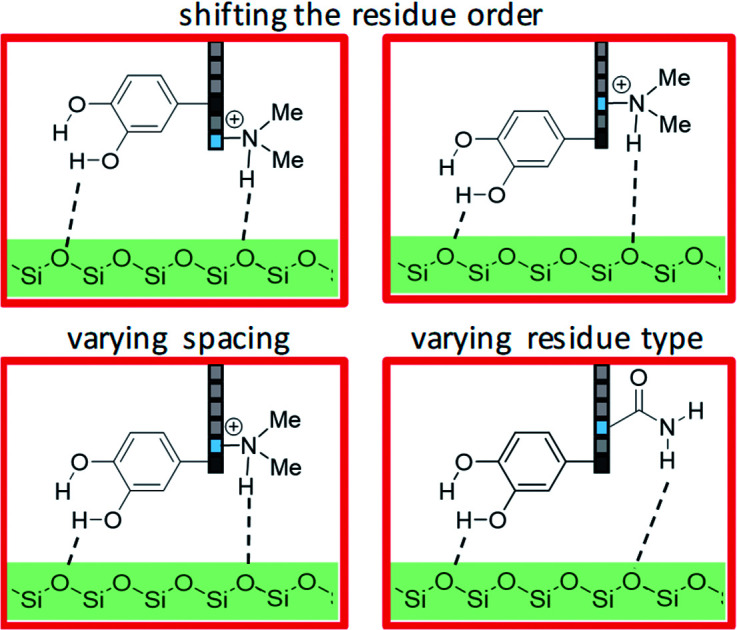

## Introduction

Marine organisms such as mussels, barnacles, or sandcastle worms are prime examples of biological wet adhesion. They exhibit strong attachments to inorganic and organic surfaces in aqueous medium, even in the presence of high salt concentrations.^1,2^ In aqueous environment, the adhesion is inhibited by both water and hydrated salt ions through the formation of thin layers preventing the direct contact between adhesive groups at the material surfaces.^3,4^ Mussels in particular have evolved adhesive proteins (mussel foot proteins, Mfps) that circumvent this problem by displacing the hydration layers and then bridging to the surface *via* strong bonding primarily through l-3,4-dihydroxyphenylalanine (DOPA) groups.^5–7^ Recent findings state that the high amount of DOPA in proximity to cationic amino acids is responsible for these unique properties.^8–10^ This synergistic effect between DOPA and primary amines is due to dispatching the hydration layer of the surface *via* charged amines allowing the catechol residues to bind to the surface. Such synergy between catechol (DOPA) and charged groups could be confirmed using synthetic polymers combining anionic and cationic residues.^11–13^ Inspired by the adhesive properties of the Mfps, a wide range of polymers with high DOPA content were synthesized toward advanced adhesives and surface coatings.^1,6,14–23^ However, sequence effects like the spacing of the charged groups and catechol residues were given little attention for the design of such mussel-inspired synthetic adhesives.

The adhesive proteins of mussels contain a large amount of DOPA and amine residues, *e.g.* Mfp-5 carries 30 mol% DOPA and 28 mol% amines, which are usually in close proximity.^23^ However, another class of residue typically represented at higher than 10 mol% (in Mfp-2, Mfp-3, Mfp-4, and Mfp-6) is asparagine carrying a primary amide.^24–27^ Asparagine as a “helix-breaker” residue is believed to increase the flexibility of the Mfps improving the accessibility of the adhesive DOPA groups. Intriguingly, for Mfp-3 the amide side chains are predominantly found in direct proximity to amine and DOPA residues.^27^ The function of Mfp-3 as a primer for strong underwater adhesion has been shown by direct adhesion measurements *via* atomic force microscopy or the surface force apparatus,^7,28^ but the role of amide side chains on adhesion has not been studied so far. Therefore, in this study we present the synthesis of sequence-defined oligo(amidoamine)s carrying selected combinations of catechol, tertiary amine and primary amide residues, similar to the arrangement of arginine, DOPA and amine residues found in Mfp-3 and study their adhesion energies on glass surfaces.

## Results and discussion

### Synthesis of sequence defined oligomers

**Fig. 1:**
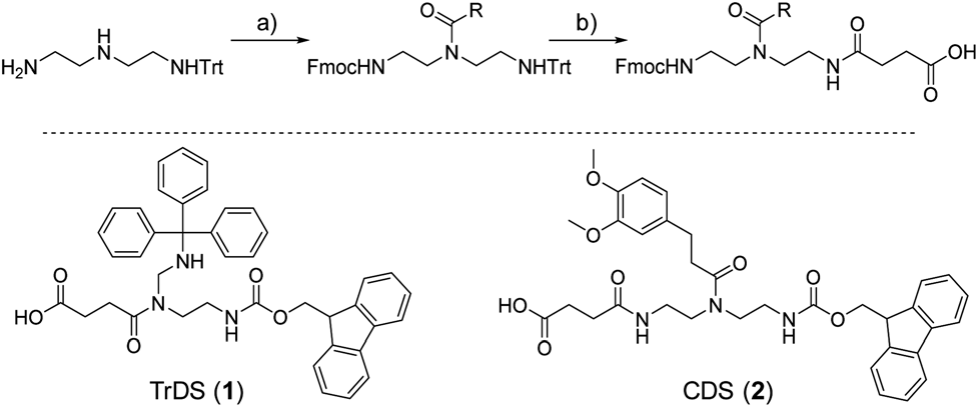
New synthesis route towards functional building blocks; (a) Fmoc-OSu, 3 eq. triethylamine in THF at −78 °C followed by 1 eq. activated acid; (b) 10 eq. TFA in DCM followed by precipitation and 1 eq. succinic anhydride, 3 eq. triethylamine in DCM.

As a cationic residue a tertiary amine was chosen to prevent crosslinking with the catechols particularly at higher pH. In addition, choosing this non-natural cationic residue instead of primary amines might provide additional indication that the catechol–amine synergy is due to the removal of the hydration layer by the charge effect and not due to additional hydrogen bonding by the amines. Along these lines, as a non-natural spacer building block between the catechol, amine and amide residues we use a short ethylene glycol chain (EDS block) to show the feasibility of transferring the catechol driven adhesion mechanism to synthetic polymers. The oligomer synthesis was adapted from an already established method using tailor-made building blocks for solid phase assembly to generate the sequence-defined structures.^29,30^ Similar to solid phase peptide synthesis, the building blocks carry both, a carboxy and an Fmoc protected primary amine group, that allow step-wise chain growth on an amine functionalized resin. Here two new building blocks were synthesized, one carrying a protected catechol moiety and one carrying an orthogonal protected primary amine, to later introduce the tertiary amine and primary amide *via* amide coupling on solid support (Fig. 1).

**Fig. 2:**
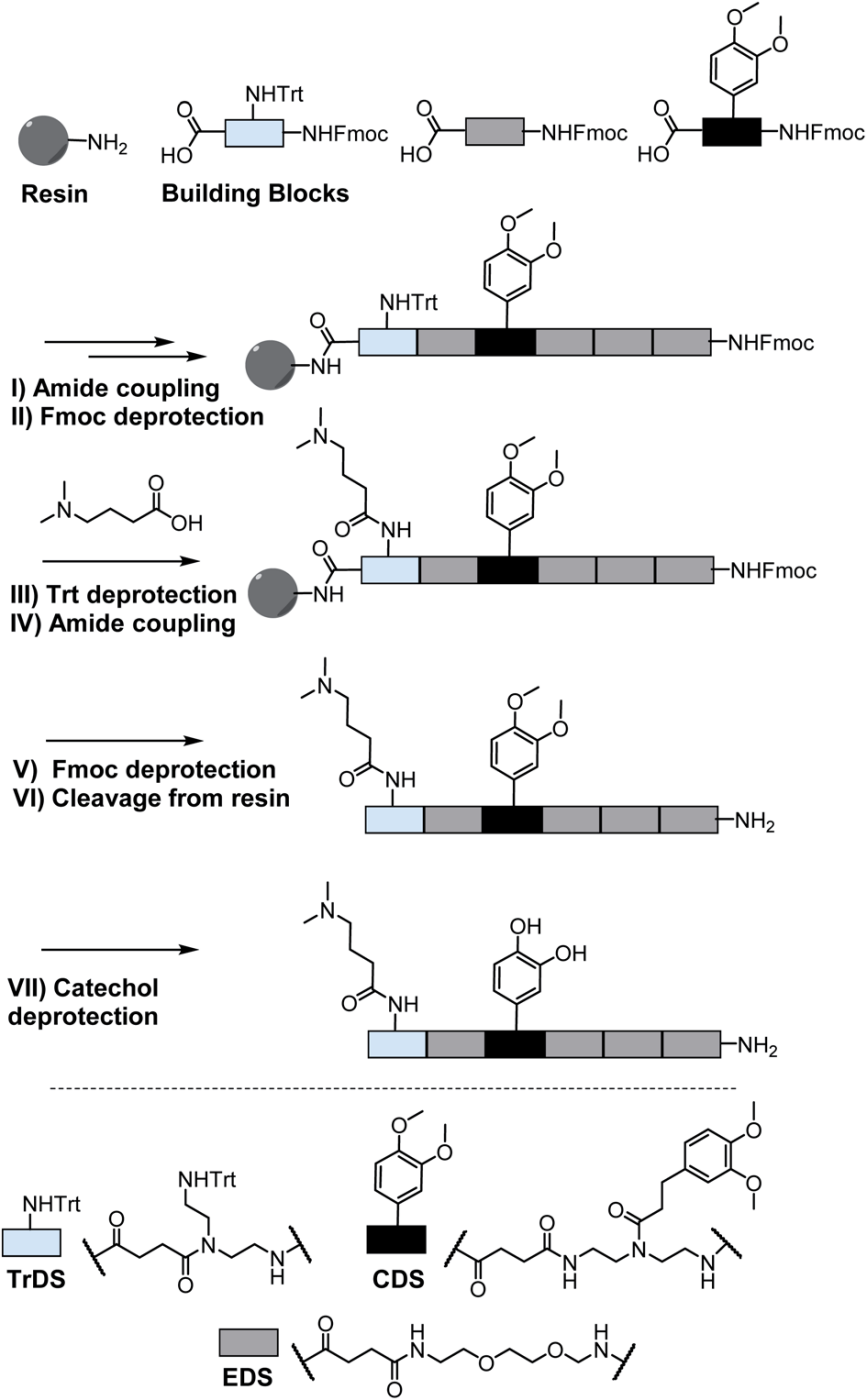
Exemplary scheme for solid phase synthesis of an oligomer using a rink amide resin; (I) 5 eq. building block, 5 eq. PyBOP, 10 eq. DIPEA in DMF; (II + V) 20% piperidine in DMF; (III) 0.15 M HCl in trifluoroethanol; (IV) 10 eq. acid, 10 eq. PyBOP, 20 eq. DIPEA; (VI) 95% TFA, 2.5% DCM and 2.5% triisopropylsilane; (VII) 16 eq. trifluoromethanesulfonic acid, 8 eq. thioanisole in TFA.

A major challenge in the solid phase synthesis of sequence-defined polymers is the access to tailor-made building blocks in sufficient quantity and purity, ideally in a time and cost-efficient manner. Here, an advanced method providing the required building blocks was developed streamlining the previous approach to a straightforward 3-step route with greatly improved atom economy and higher yields.^29,31–34^ In the first step, one of the two primary amines of diethylenetriamine was protected using trityl chloride. Afterward, the second primary amine was selectively converted using Fmoc-OSu in THF at −78 °C, with subsequent addition of an activated acid which carries the desired side chain functionality. The last step includes the cleavage of the trityl group and reaction with succinic anhydride. With this new protocol two different building blocks were synthesized. The first building block TrDS (**1**) offers a trityl protected amine, orthogonal to the Fmoc protection group, for further functionalization during solid phase synthesis. The second novel building block CDS (**2**) was developed to introduce a methyl ether protected catechol moiety in the side chain using the acyl chloride of 3-(3,4-dimethoxyphenyl)propionic acid. This protecting strategy ensured stability during acidic conditions of the building block synthesis as well as basic conditions during solid phase synthesis. Together with the previously introduced building blocks EDS, TrDS, and CDS, solid phase supported synthesis following previously reported coupling conditions was applied (Fig. 2). The oligomer scaffold was assembled by step-wise amide coupling and subsequent Fmoc deprotection of the terminal amine. For the introduction of side chains presenting a tertiary amine or primary amide groups, the TrDS building block was used: after full synthesis of the backbone, the trityl group of TrDS was cleaved using 0.15 M HCl in trifluorethanol, a condition resulting in full release of the trityl group while maintaining stability of the acid labile solid support.^35^ Next, the desired side chain functionalities were introduced by coupling the corresponding carboxylic acid using PyBOP as a coupling reagent. After cleavage of the oligomer from the solid phase, the catechol moieties were deprotected using trifluormethanesulfonic acid and thioanisole in trifluoracetic acid following a procedure previously introduced by Kiso *et al.*^36^ Full deprotection and successful isolation of the desired oligomer structures were confirmed by ^1^H-NMR, ^13^C-NMR and HR-ESI MS (see ESI S4†).

**Fig. 3:**
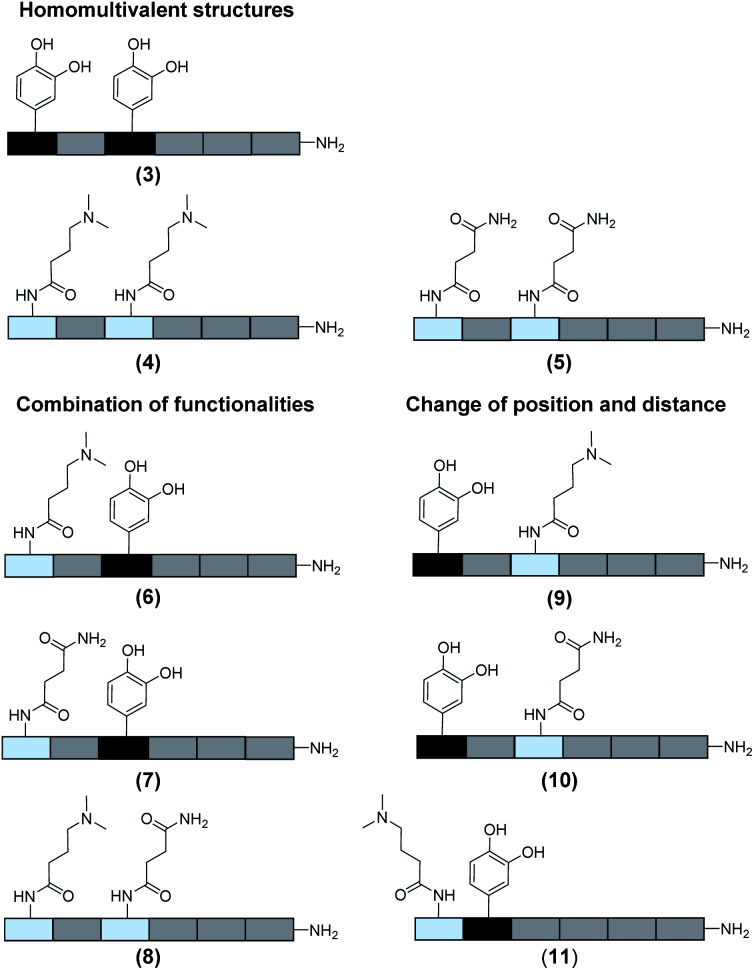
Overview of the oligomers.

In total 9 different oligomers were synthesized (Fig. 3). All structures carry a terminal amine group for later coupling onto microgels and use in adhesion studies. In order to study combination and positioning effects of the different functional groups on adhesion, various sets of oligomers were synthesized. As homofunctional structures, the oligomers **3–5** each carry two identical functional groups, either catechol, tertiary amine, or primary amide both in position 1 and 3. Oligomers **6**, **7** and **8** combine two of the functional groups to form the three possible combinations. Oligomers **9** and **10** change the position of catechol and amine or amide, to investigate the influence of the order of functional groups. In addition, oligomer **11** reduces the spacing between amine and catechol. All oligomers have a length of six building blocks with the EDS building blocks serving as spacers between the functional building blocks keeping the overall size of all oligomers the same. Importantly, for all catechol bearing structures, oxidation in water was not observed within several days (see ESI S8†). Therefore, we assume that in the course of the following adhesion studies, catechol–quinone transitions did not take place.

### SCP preparation and adhesion measurements

**Fig. 4:**
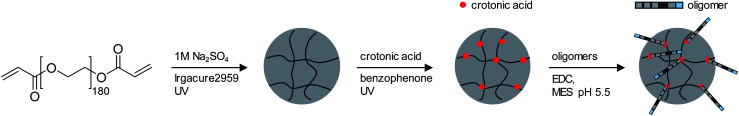
Synthesis of PEG based SCPs. (1) liquid–liquid phase separation of PEG macromonomers in 1 M NaSO_4_ followed by UV crosslinking; (2) photochemical grafting of crotonic acid using benzophenone; (3) coupling of oligomers by carbodiimide chemistry.

For the adhesion measurements, soft microgels (soft colloidal probes, SCPs) based on poly(ethylene glycol) (PEG) were functionalized with the sequence-defined oligomers (**3–11**) and allowed to settle and bind to glass surfaces.^37^ The glass surfaces were used here as a model for inorganic silica-based materials. To prepare the SCPs, microdroplets of poly(ethylene glycol diacrylamide) were formed *via* liquid–liquid phase separation in a concentrated sodium sulphate solution followed by UV crosslinking (Fig. 4).^38^ The oligomers were introduced by grafting of crotonic acid under UV irradiation in presence of benzophenone followed by the repeated coupling of the oligomers *via* carbodiimide chemistry. The degree of oligomer functionalization in the PEG network was determined in two steps *via* titration with toluidine blue, a crotonic acid binding dye.^37^ First, the amount of crotonic acid was determined before coupling the oligomers. Second, the residual, unreacted crotonic acid residues were titrated after the oligomer coupling step. The coupling efficiency was larger than 90%, and the oligomer functionalization degrees were determined as ∼86 μmol per gram PEG (see ESI S5†). Hence, 13.5–14.2 wt% of the PEG-SCPs are oligomers. Using the SCP elastic moduli as an estimate for the specific volume in of PEG in water,^39^ the PEG swelling degree can be calculated giving an oligomer concentration of 11 mmol l^−1^ in the SCP scaffold.^37^

**Fig. 5:**
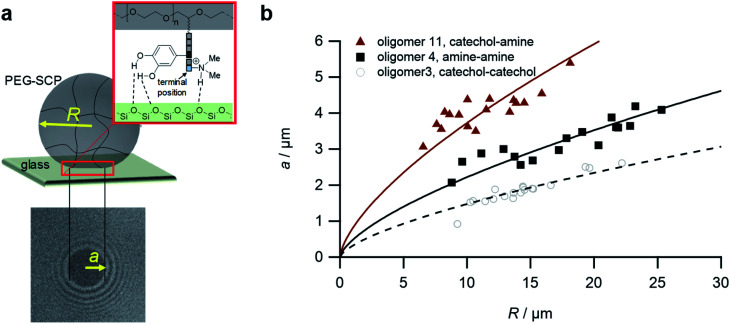
The SCP adhesion assay. (a) Schematic representation of an oligomer-functionalized SCP adhering to a glass slide. The reflection interference contrast microscopy image (bottom) shows a typical contact area (dark area in the center) and newton rings providing the geometry of the SCP, *i.e.* the parameters *a* and *R*. (b) Typical JKR plots and fits (lines) according to eqn (1) depicting the oligomers **3** (empty circles), **4** (squares) and **11** (triangles).

Upon adhesion, the SCPs mechanically deform and form distinct contact areas with the glass surface. To quantify the SCP-adhesion energies (*W*_adh_) on glass, the contact radii (*a*) were measured by micro-interferometry (Fig. 5) and evaluated by the Johnson–Kendall–Roberts (JKR) model of adhesion:^40–42^1
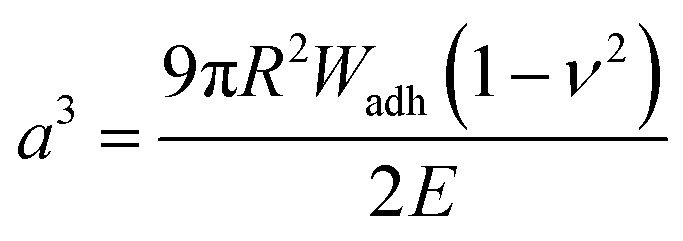
where *W*_adh_ is the adhesion energy, *E* is the elastic modulus of the SCPs, and *ν* the Poisson ratio. The adhesion energies were read from the plots of the contact area *a* and the SCP radius *R* (Fig. 5). The SCP method allows detecting adhesion energies with high precision and has been broadly applied, *e.g.* to study biomolecular interactions,^42,43^ hydrophobic forces,^44^ and analytes in the solute by very sensitive competitive binding assays.^41,45^

**Fig. 6:**
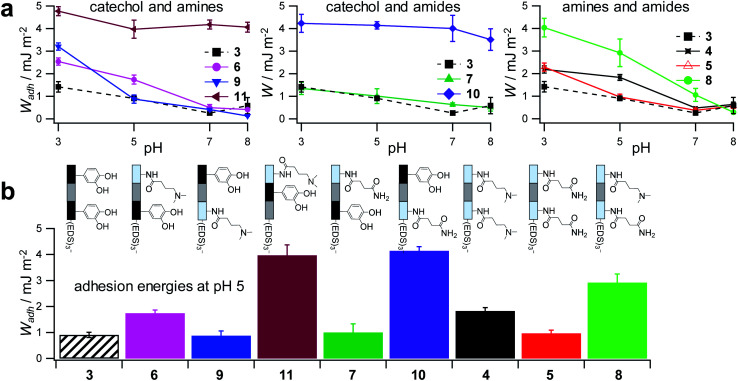
Adhesion energies measured for oligomer-functionalized SCPs (a) measurements against a glass surface in 0.1 M sodium chloride solution from pH 3 to pH 8. (b) Adhesion energies mimicking the pH during protein secretion in initial mussel adhesion.^2^ Oligomer concentration normalized adhesion energy values are very similar to non-normalized values (ESI S9†).

To control the solute conditions, the SCP-adhesion assay was conducted in 0.1 M sodium chloride and between pH 3–8 (Fig. 6). The pH controls the glass surface charge by protonation/deprotonation of the silanol groups, which broadly affects the adhesion. At low pH the surface is able to donate hydrogen bonds to the ethylene glycol groups at the PEG and EDS backbone, whereas almost complete deprotonation is expected at pH 7,^46^ rendering the surface unable to donate hydrogen bonds. In addition, the hydration barrier is stronger for charged surfaces at high pH.^47^ This explains the observed overall decreasing adhesion energies with increasing pH for all oligomers (Fig. 6b). The measurements confirmed the synergistic effect between cationic amines and catechols since the catechol/amine (**6**, **9** and **11**) combinations always achieve higher adhesion when compared to catechol/catechol (**3**). This shows that the catechol/amine synergy also works with tertiary amines instead of the natural primary amines supporting the hypothesis that it is the charge-induced displacement of the hydration layer that increases catechol binding. With the sequence-controlled oligomers we could additionally show the effect of catechol/amine spacing. In case where the catechol and amine residues are in close vicinity (**11**), the adhesion energy is drastically amplified compared to the oligomers with an additional EDS spacer between catechol and amine (**6** and **9**). In addition, the adhesion was affected by changing the position of the catechol and amine residues (**6** and **9**). When the amine is located at the terminating position (the free chain end not attached to the SCP) (**6**), the decrease in adhesion between pH 3 and pH 5 is not as strong when compared to the oligomer with the catechol at the terminating position (**9**). This could be due to the increased ionic interactions between the terminal amine and the partially deprotonated surface at pH 5 compensating the loss of silanol hydrogen bonding at elevated pH. Comparison with structures that do not contain catechol but combinations of amines and primary amide side chains confirm this trend (**4**, **5** and **8**). The amides can interact with the silica groups at the surface *via* hydrogen bonding but when cationic amines are included (**8**) the adhesion appears to be stronger at elevated pH on the anionic glass surface due to additional ionic bonding. Overall, these results agree with earlier studies on the synergistic adhesion effects of amine and catechol residues,^8,9^ but for the first time show that their positioning and spacing is of key importance to maximize such synergy. Along these lines, *via* dynamic single molecule adhesion measurements Li *et al.*^10^ found that reversing the amine catechol positioning affects the adhesion, which they attributed to a different load distribution within the molecules upon pull-off.

Surprisingly, the combination of amide and catechol residues showed an even larger dependence on the residue positioning. In case the catechol is the terminating group (**10**), the adhesion energy is significantly stronger when compared to placing the amide at the chain end (**7**). The adhesion is even stronger when compared to amine/catechol combinations with similar spacing (**6** and **9**). This suggests that there are additional interactions amplifying the catechol-mediated adhesion with the glass surface, similar to the amine/catechol synergism. For amide/catechol combinations this could be in part due to the ionic resonance structure of the primary amide (25–30% ionic character)^48^ helping to displace the surface hydration layer on the glass surface. In addition, we hypothesize that there is an intricate balance between intra- and intermolecular hydrogen bond interactions. Intramolecular hydrogen bonding of the functional side chains would reduce their interaction with the surface and thus the overall adhesion. It seems that the introduction of primary amide side chains shifts this balance toward promoting adhesion. We have observed previously for sequence-controlled oligomers mimicking biopolymers that indeed the positioning of residues and the resulting variations in the conformation of the molecule play a key role for their intermolecular interactions *e.g.* when targeting protein receptors.^49^ We cannot conclude yet on the mechanisms of increasing catechol-mediated adhesion when introducing primary amide side chains but when looking back at the natural role model, Mfp-3, DOPA moieties are indeed very often accompanied by neighbouring asparagine building blocks. Thus the effect we observe here is likely to take place also in the natural mussel adhesives. The spacing of the functional residues in the natural Mfps can be much shorter, usually one to four amino acids apart, compared to the spacing in the oligomers, which is equivalent to a spacing of about seven amino acids. Nevertheless, we could still detect a synergy between the functional groups, perhaps due to the coiling of the flexible backbone resulting in shorter effective spacings. This indicates that the synergy of the functional groups could be transferred to structurally different synthetic polymers.

## Conclusions

Taken together, combining catechols and amines on a scaffold promotes wet adhesion in accordance with the literature.^8–10^ Intriguingly, the spacing of these residues on the polymer chain strongly affects adhesion to negatively charged silica surfaces. Charged moieties and catechols should be very close to maximize adhesion, which is also in accordance with their positioning in the mussel adhesion proteins. Notably also non-natural charged residues such as the tertiary amines used here are capable of increasing the catechol binding due to the displacement of hydration layers and condensed ions. In addition, introducing other functional groups present in the natural sequences such as primary amides may also have synergistic effects on adhesion as they showed increased adhesion in comparison to the amine/catechol combinations in this study. Further studies will be required to reveal the molecular mechanisms behind a potential synergy between amides and catechols and the effect of polymer conformation on catechol driven adhesion. Although details of the potential mechanism remain unknown, this shows that there is still much to be learned and much to be gained by controlling the positioning of interacting residues in bio-inspired sequence-controlled polymers.

## Conflicts of interest

The authors filed a patent application DE 102019208832.5 “Polymere für die Behandlung von Oberflächen”.

## Supplementary Material

SC-011-D0SC03457F-s001
