# Supplementary material for: Sequence-defined positioning of amine and amide residues to control catechol driven wet adhesion
Source: Chem Sci. 2020 Aug 31;11(36):9919–24. doi: 10.1039/d0sc03457f (PMC8162180; doi:10.1039/d0sc03457f)
Supplement: SC-011-D0SC03457F-s001 [file SC-011-D0SC03457F-s001.pdf]

Electronic Supplementary Material (ESI) for Chemical Science.
This journal is © The Royal Society of Chemistry 2020

Supporting Information

Sequence-defined positioning of amine and amide residues to control catechol driven wet adhesion.

Lukas Fischer,†[a] Alexander K. Strzelczyk, †[a] Nils Wedler,[b] Christian Kropf,[b] Stephan Schmidt*[a] and Laura Hartmann*[a]

[a] *Institut für Organische und Makromolekulare Chemie, Heinrich-Heine-Universität Düsseldorf, Universitätsstr. 1, 40225 Düsseldorf, Germany.*
[b] *Laundry & Home Care, Henkel AG & Co. KGaA, Henkelstr. 67, 40589 Düsseldorf, Germany.*

Contents

S1 Materials and methods 2
Materials 2
Oligomer synthesis 2
Soft colloidal probe (SCP) synthesis 3
SCP characterization 3
S2 Instrumentation 3
Nuclear Magnetic Resonance Spectroscopy (NMR) 3
High Resolution – Mass Spectrometry (HR-MS) 4
Reversed Phase – High Pressure Liquid Chromatography (RP-HPLC) 4
Preparative Reversed Phase – High Pressure Liquid Chromatography (Prep-RP-HPLC) 4
Freeze Dryer 4
S3 Building Block Synthesis and Chemical Analysis 5
S4 Oligomer Synthesis and Chemical Analysis 11
On Resin Deprotection Of Trityl 11
Side Chain Coupling 11
Deprotection Of Catechols 11
Oligomer Chemical Analysis 11
S5 Determination of SCP Functionalization Degrees 46
Oligomer Functionalization of PEG-CA-SCPs 46
Crotonic Acid Titration via UV-VIS Spectroscopy 47
Determination of oligomer functionalization degree via microscope based TBO titration 47
S6 Determination of the SCPs elastic modulus 48
S7 Reflection Interference Contrast Microscopy (RICM) measurements Setup 49
Determination of the Contact Radius 49
Correction Factors 50
Contact radius determination 50
S8 Stability of the catechol group 51
S9 Non-normalized and oligomer concentration normalized adhesion energy values 52
Supporting References 53

Supporting Information

S1 Materials and methods

Materials

Triisopropylsilane (TIPS) (98%), triethylsilane (99%) and 4-(dimethylamino)butyric acid hydrochloride (98%) were purchased from Sigma-Aldrich. Diisopropylethylamine (DIPEA) (≥99%) was purchased from Carl Roth. Dimethylformamide (DMF) (99.8%, for peptide synthesis), piperidine (99%), triphenylmethyl chloride (Trt-Cl) (98%) and succinic anhydride (99%) were purchased from Acros Organics. Dichloromethane (DCM) (99.99%), sodium chloride (99.98%), tetrahydrofuran (THF) (analytical reagent grade), ethyl acetate (analytical reagent grade) and sodium hydrogen carbonate (analytical reagent grade) were purchased from Fisher Scientific. Triethylamine (pure) was purchased from AppliChem. Trifluoroacetic acid (TFA) (99%) and (benzotriazol-1-yloxy)tripyrrolidinophosphonium hexafluorophosphate (PyBOP) (98%) were purchased from Fluorochem. Succinamic acid (97%) and Thioanisol (99%) were purchased from Alfa Aesar. 3-(3,4-Dimethoxyphenyl)propionic acid (99%) was purchased from BLD Pharmatech Ltd. Fmoc-Osu (99%), trifluoromethanesulfonic acid (98%) and trifluoroethanol (99%) were purchased from Carbolution. Diethyl ether (contains BHT as inhibitor, >99%) was purchased from Honeywell. Tentagel® S RAM resin was purchased from Rapp Polymere. Sodium sulfate (99.5%) was purchased from fisher chemicals. Polyethylene glycol diacrylate (PEG(8000)-DiAc) was purchased from Alfa Aesar. Irgacure 2959 (98%) and crotonic acid (98%) were purchased from Sigma-Aldrich. Benzophenone (99%) was purchased from Acros Organics. 1-Ethyl-3-(3-dimethylaminopropyl)carbodiimid-hydrochlorid (EDC HCl) (≥99%) was purchased from Carl Roth. Water was purified with a Milli-Q system (Millipore) obtaining a final resistivity of 18 MΩcm.

Oligomer synthesis

All oligomers were synthesized using the building blocks EDS, TrDS and CDS as previously described.[1] The oligomers were assembled via iterative deprotection and amide coupling on a Tentagel® S RAM resin. For deprotection, the resin was treated with 20% piperidine in DMF (2x 15 min) and washed with DMF (10x). For the coupling step, the building block (5 eq.) and PyBOP (5 eq.) were dissolved in DMF and DIPEA (10 eq.) was added. The resin was treated with the coupling solution for 1 hr with subsequent DMF washing (10x). After assembly of the full sequence, the trityl groups were cleaved by treating the resin with 0.1 M HCl in trifluorethanol (2x1.5 h). Then the resin was washed with DMF (5x) and the free amines were deprotonated with 20% DIPEA in DMF for 10 minutes. For introducing the side chains, the resin was treated for 1 hr with a solution of solution of either succinamic acid or 4-(dimethylamino)butyric acid (5 eq.), PyBOP (5 eq.) and DIPEA (10 eq.) in DMF and washing in DMF (10x) afterward. The structures were cleaved from solid support with a solution of TFA/TIPS (95/5), precipitated in diethyl ether and the precipitate was lyophilized. All oligomers with a protected catechol moiety were deprotected by treatment with 16 eq. trifluoromethanesulfonic acid and 8 eq. thioanisole per methyl ether in TFA for 16 h. Afterward the

reaction solution was precipitated in diethyl ether and the deprotected oligomers were lyophilized. The chemical analysis of the building block and oligomers are shown in the supporting information S1-S4)

Soft colloidal probe (SCP) synthesis

A dispersion of poly(ethylene glycol diacrylamide) (PEGdAAm, 50 mg, 6.3 µmol, Mn = 8000 Da) microdroplets was prepared by phase separation in 10 mL 1M sodium sulfate solution under vigorous agitation.[2] Irgacure 2959 (2.1 mg, 5.4 µmol) was added and the dispersion was photopolymerized under UV light for 90 s (Heraeus HiLite Power curing unit (Heraeus Kulzer, Germany). The diameter of received microgels was between 10-40 µm. After centrifugation/washing, crotonic acid was grafted onto the SCPs by exchange of water with ethanol, addition of benzophenone (250 mg, 1.4 mmol) and crotonic acid (1.5 g, 17.7 mmol) flushing with nitrogen for 60 s followed by UV irradiation for 1080 s.[3] The particles were washed with ethanol and water to remove all reactants. In the final step, the oligomers with unprotected amine end groups were coupled to crotonic acid on the SCPs in 0.1 M MES buffer pH 5.5 containing 32.5 mM (1-ethyl-3-[3-dimethylaminopropyl]carbodiimide hydrochloride), and 0.225 mM oligomers followed by washing with water. The carbodiimide coupling was repeated to maximize the functionalization degree for all oligomers (supporting information S5).

SCP characterization

AFM force-indentation measurements with a NanoWizard 2 system (JPK instruments AG, Berlin, Germany) was performed to determine the elastic moduli of the SCPs. As AFM probe a silica particle with a diameter of 4.6 µm was adhered with epoxy glue onto a tipless, non-coated cantilever (spring constant 0.32 N/m; CSC12, NanoAndMore GmbH). Several force curves were recorded for different SCPs and analyzed with an appropriate contact model developed by Glaubitz et al. (supporting information S6). The degree of oligomer functionalization in the SCP network was determined by titrating crotonic acid residues with toluidine blue O (TBO). 1.0 mL of a dispersion containing crotonic acid functionalized SCPs were dried by first exchanging the water by ethanol in the continuous phase and then treating in a vacuum oven at 50°C until constant weight. After the dry mass was determined, 1.0 mL of 312.5 µM TBO solution at pH10 was added and shaken in the dark for 12 hrs. Next, 0.3 mL of the TBO solution supernatant of the was diluted with 1.7 mL water at pH 10 and the absorbance at 633 nm was detected and compared to the TBO reference (no infusion of SCPs) to calculate the degree of crotonic acid functionalization. Comparing the amount of SCP crotonic acid functionalization before and after the oligomer coupling gave the oligomer functionalization degree (supporting information S5).

S2 Instrumentation

Nuclear Magnetic Resonance Spectroscopy (NMR)

^{1}H-NMR and ^{13}C NMR were recorded on a Bruker Avance III 300, a Bruker Avance DRX-500 or a Bruker Avance III 600. Chemical shifts were reported as delta (δ) in parts per million (ppm) and

coupling constants as *J* in Hertz (Hz). Multiplicities are stated as following: s = singlet, d = doublet, t = triplet, q = quartet, m = multiplet.

High Resolution – Mass Spectrometry (HR-MS)

HR-MS measurements were conducted on a Bruker UHR-QTOF maxis 4G with a direct inlet via syringe pump, an ESI source and a quadrupole Time of Flight (QTOF) analyzer. Samples were dissolved in water with a concentration of 1 mg/mL.

Reversed Phase – High Pressure Liquid Chromatography (RP-HPLC)

RP-HPLC was performed with an Agilent 1260 Infinity instrument coupled to a variable wavelength detector (VWD) set to 214 nm. As a column a Poroshell 120 EC-C18 1.8 µM (3.0x50 mm, 2.5 µm) reversed phase column was used. The mobile phase A consisted of 95/5 H_2O/MeCN with 0.1% formic acid and mobile phase B consisted of 95/5 MeCN/H_2O with 0.1% formic acid. The flowrate for all measurements was 0.4 mL/min.

Preparative Reversed Phase – High Pressure Liquid Chromatography (Prep-RP-HPLC)

Prep-RP-HPLC was conducted on an Agilent 1260 Infinity instrument coupled to a variable wavelength detector (VWD) set to 214 nm. As a column a CAPCELL PAL C18 (20mmI.D. x 250 mm, 5 µm) reversed phase column was used. The mobile phase A consisted of H_2O with 0.1% formic acid and mobile phase B consisted of MeCN with 0.1% formic acid. All samples were purified with a flowrate of 10 ml/min and a gradient of 100% A to 50% A over 15 min. Fractions were collected by an automated collector and were then lyophilized.

Freeze Dryer

Lyophilization of the final structures was conducted on an Alpha 1-4 LD plus instrument from Martin Christ Freeze Dryers GmbH. The lyophilization was done at a pressure of 0.1 mbar.

S3 Building Block Synthesis and Chemical Analysis

The building block EDS was synthesized according to literature.[4]

Synthesis Route for Functional Building Blocks

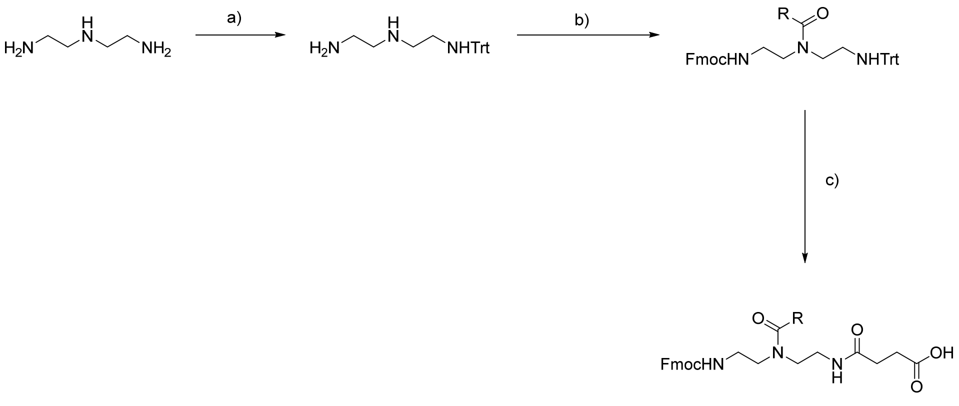

Figure S1. Overview of building block synthesis route: a) 0.25 eq. trityl chloride in DCM; b) 1 eq. Fmoc-OSu, 3 eq. triethylamine in THF at -78°C for 2 h followed by 1 eq. activated acid; c) 10 eq. TFA in DCM for 1 h followed by precipitation and 1 eq. succinic anhydride, 3 eq. triethylamine in DCM for 2 h.

Functional building blocks were synthesized with the new synthesis route shown in Figure S1.

a) To a solution of diethylenetriamine in DCM a solution of trityl chloride (0.25 eq.) in DCM was added over 1 h at 0°C. The reaction was stirred for 16 h at room temperature and afterwards extracted with a saturated $NaHCO_3$ solution (3x). The organic phase was dried with $MgSO_4$ and the solvent was evaporated under reduced pressure to give the crude product as a brown oil.
b) The crude product of a) was dissolved in THF and triethylamine (3 eq.) and a solution of Fmoc-OSu (1 eq.) in THF was added over 2 h at -78°C. Afterwards the activated acid (1 eq.) in THF was added and the reaction was stirred for 16 h at room temperature. The reaction mixture was extracted with a saturated NaCl solution (3x) and the organic phase was dried with $MgSO_4$ and the solvent was evaporated under reduced pressure to give the crude product as a brown foam.
c) The crude product of b) was dissolved in DCM and triethylsilane (10 eq.) and 10 vol-% TFA were added. The reaction was stirred at room temperature for 1 h. Afterwards the solvent was evaporated under reduced pressure and the product was precipitated in diethyl ether. The precipitate was dissolved in DCM and triethylamine (3 eq.) and succinic anhydride (1 eq.) were added. The reaction was stirred for 2 h at room temperature and afterwards extracted with a citric acid solution (3x). The organic phase was dried with $MgSO_4$ and the solvent was evaporated under reduced pressure to give the crude product as a brown foam.

4-((2-((((9H-fluoren-9-yl)methoxy)carbonyl)amino)ethyl)(2-(tritylamino)ethyl)amino)-4-oxobutanoic acid (TrDS) (**1**)

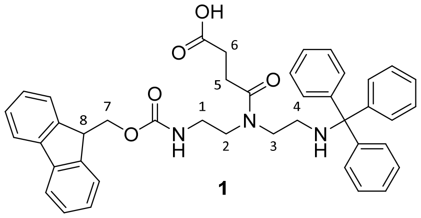

1

TrDS (**1**) was synthesized following the synthesis route in Figure S1 (step a and b). The crude product was recrystallized in DCM and diethyl ether (1:1) to give a white powder with a yield of 24 g (72%).

^{1}H-NMR (600 MHz, DMSO-*d*$_{6}$, 80°C) δ [ppm]: 7.86 (d, *J* = 7.6 Hz, 2H, Fmoc-H), 7.67 (d, *J* = 7.4 Hz, 2H, Fmoc-H), 7.45-7.35 (m, 8H, Fmoc-H, Trt-H), 7.32 (t, *J* = 7.6 Hz, 2H, Fmoc-H), 7.29 (t, *J* = 7.7 Hz, 6H, Trt-H), 7.19 (t, *J* = 7.3 Hz, 3H, Trt-H), 4.44-4.29 (m, 2H, H-7), 4.25-4.20 (m, 1H, H-8), 3.30-2.55 (m, 8H, H-1,H-2,H-3,H-4), 2.50-2.20 (m, 4H, H-5,H-6).

^{13}C-NMR (126 MHz, $CDCl_3$+DMSO-*d*$_{6}$) δ [ppm]: 174.25, 172.26, 156.26, 156.13, 145.16, 143.47, 140.65, 128.05, 127.94, 127.43, 127.15, 126.56, 125.92, 124.70, 124.61, 119.40, 119.38, 70.43, 65.73, 65.21, 53.20, 48.47, 46.73, 45.95, 45.35, 42.00, 38.79, 31.00, 29.24, 29.10, 28.67, 27.67, 27.36, 24.93, 22.08, 14.81, 13.66.

HR-ESI-MS: calculated mass for $C_{42}H_{42}N_3O_5$ $[M+H]^+$ 668.3119, found 668.3119.

RP-HPLC (gradient from 0% to 100% eluent B over 30 min at 25°C): t_r=20.9 min, relative purity 98%.

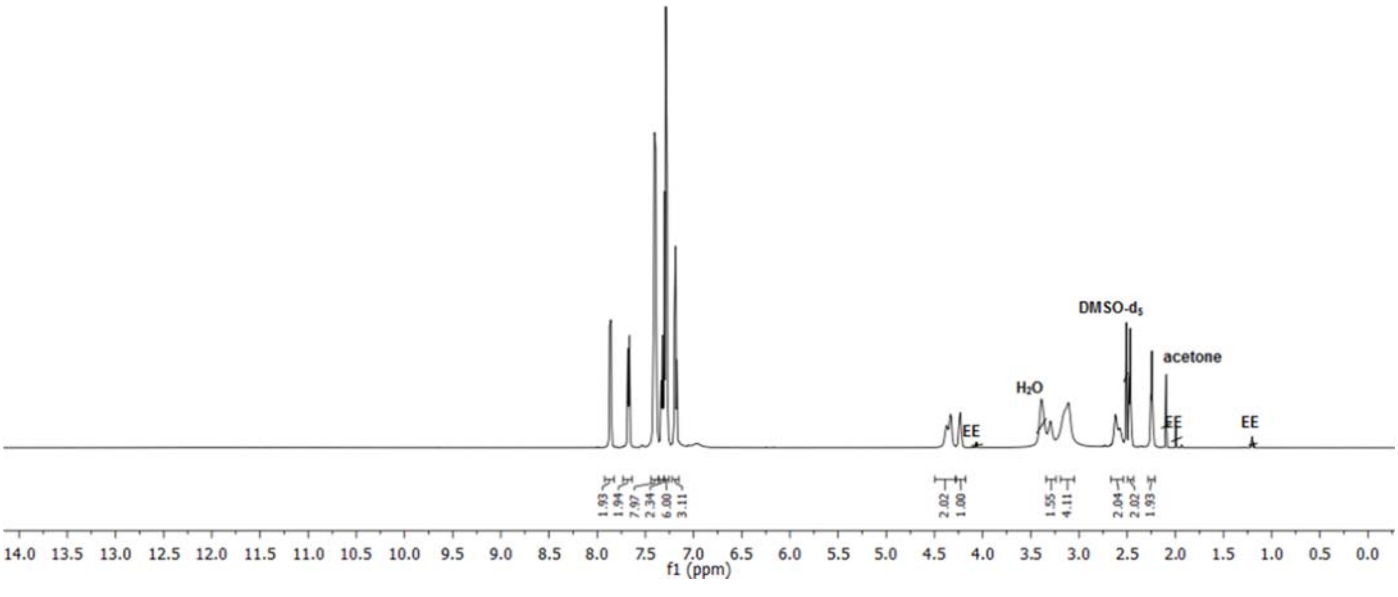

Figure S2. ^{1}H-NMR spectrum of compound **1** (600 MHz, DMSO-*d*$_{6}$, 80°C).

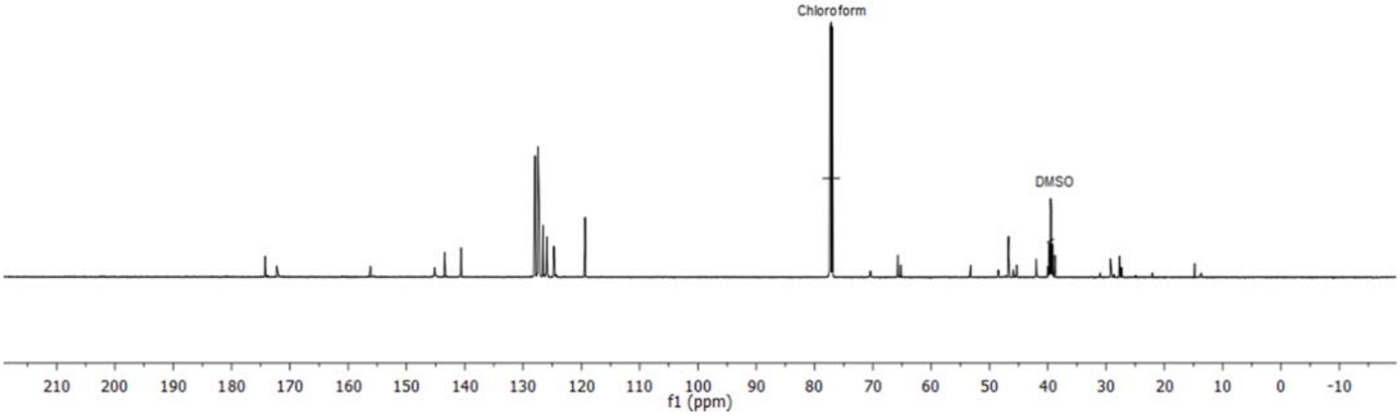

Figure S3. ^{13}C-NMR spectrum of compound **1** (126 MHz, $CDCl_3$+DMSO-d_6).

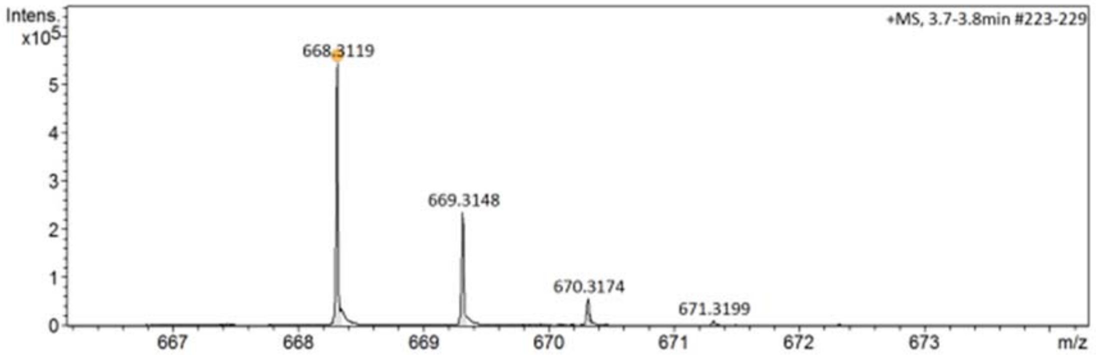

Figure S4. HR-ESI (ESI^+ Q-TOF) of compound **1**.

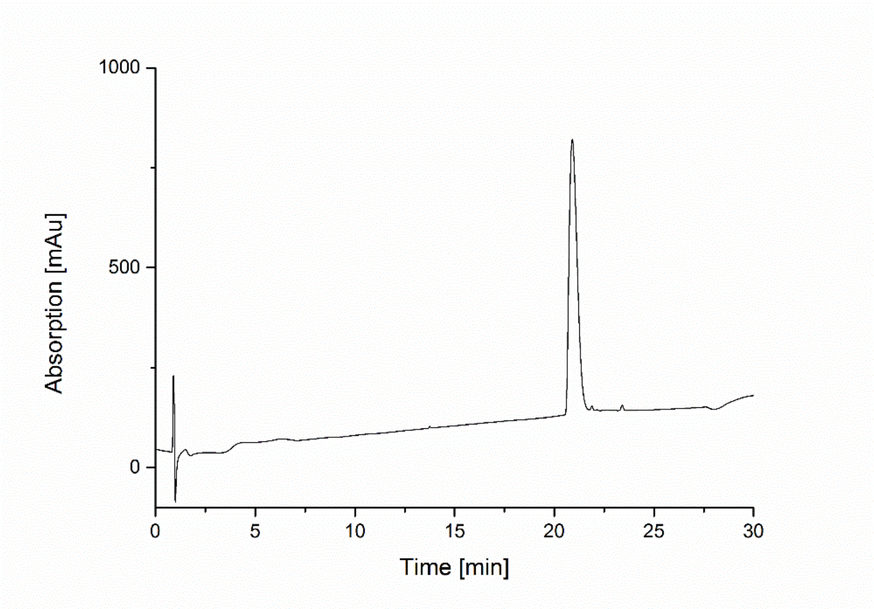

Figure S5. RP-HPLC chromatogram of compound **1** (gradient from 0% to 100% eluent B over 30 min at 25°C).

7-(3-(3,4-dimethoxyphenyl)propanoyl)-1-(9H-fluoren-9-yl)-3,11-dioxo-2-oxa-4,7,10-triazatetradecan-14-oic acid (CDS) (**2**)

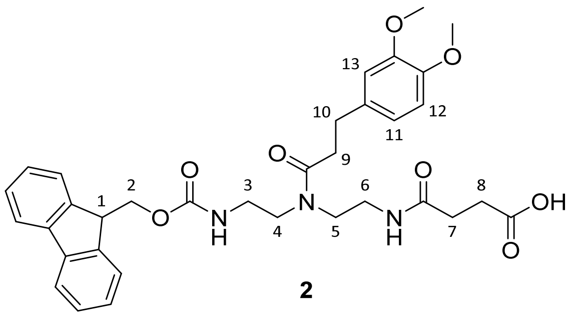

2

CDS (**2**) was synthesized following the synthesis route in Figure S1. The crude product was recrystallized in acetone and DCM (1:1) to give a white powder with a yield of 17 g (60%).

^{1}H-NMR (600 MHz, DMSO-$d_{6,}$) δ [ppm]: 8.1 (m, NH), 7.9 (m, NH), 7.88 (d, J = 7.7 Hz, 2H, Fmoc-H), 7.68 (d, J = 7.4 Hz, 1H, Fmoc-H), 7.64 (d, J = 7.5 Hz, 1H. Fmoc-H), 7.47 (m, NH), 7.41 (t, J = 7.5 Hz, 2H, Fmoc-H), 7.36-7.29 (m, 2H, Fmoc-H,NH), 6.83-6.60 (m, 3H, H-11, H-12, H-13), 4.28 (dd, J = 17.4, 7.0 Hz, 2H, H-2), 4.21 (t, J = 7.0 Hz, 0.5H, H-1), 4.16 (t, J = 7.0 Hz, 0.5H, H-1), 3.75-3.63 (m, 6H, H-14), 3.29 (m, 4H, H-4, H-5), 3.19-3.07 (m, 4H, H-3, H-6), 2.75-2.69 (m, 2H, H-9), 2.60-2.55 (m, 2H, H-10), 2.45-2.38 (m, 2H, H-7), 2.35-2.27 (m, 2H, H-8).

^{13}C-NMR (126 MHz, DMSO-d_6) δ [ppm]: 174.25, 172.26, 156.26, 156.13, 145.16, 143.47, 140.65, 128.05, 127.94, 127.43, 127.15, 126.56, 125.92, 124.70, 124.61, 119.40, 119.38, 70.43, 65.73, 65.21,

53.20, 48.47, 46.73, 45.95, 45.35, 42.00, 38.79, 31.00, 29.24, 29.10, 28.67, 27.67, 27.36, 24.93, 22.08, 14.81, 13.66.

HR-ESI-MS: calculated mass for $C_{34}H_{40}N_3O_8$ $[M+H]^+$ 618.2810, found 618.2807.

RP-HPLC (gradient from 0% to 100% eluent B over 30 min at 25°C):t_r=21.1 min, purity 99%.

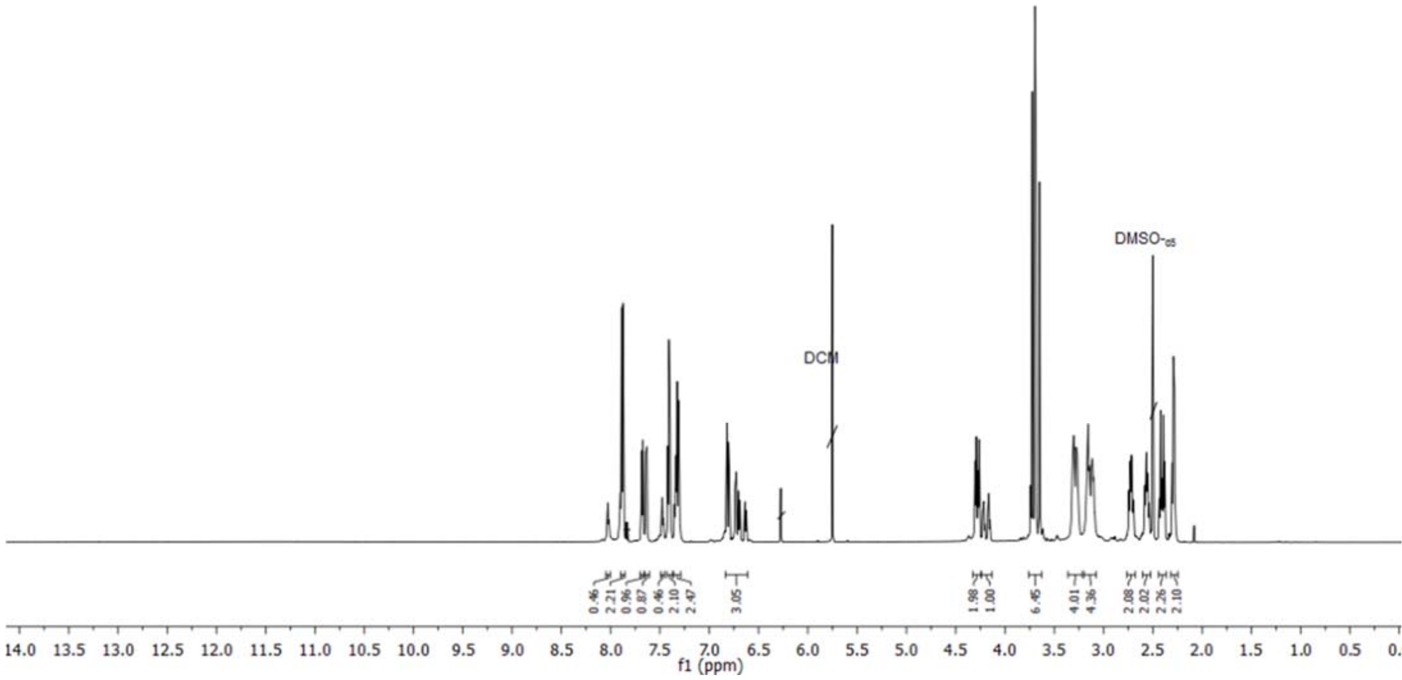

Figure S6. ^{1}H-NMR spectrum of compound **2** (600 MHz, DMSO-d_6).

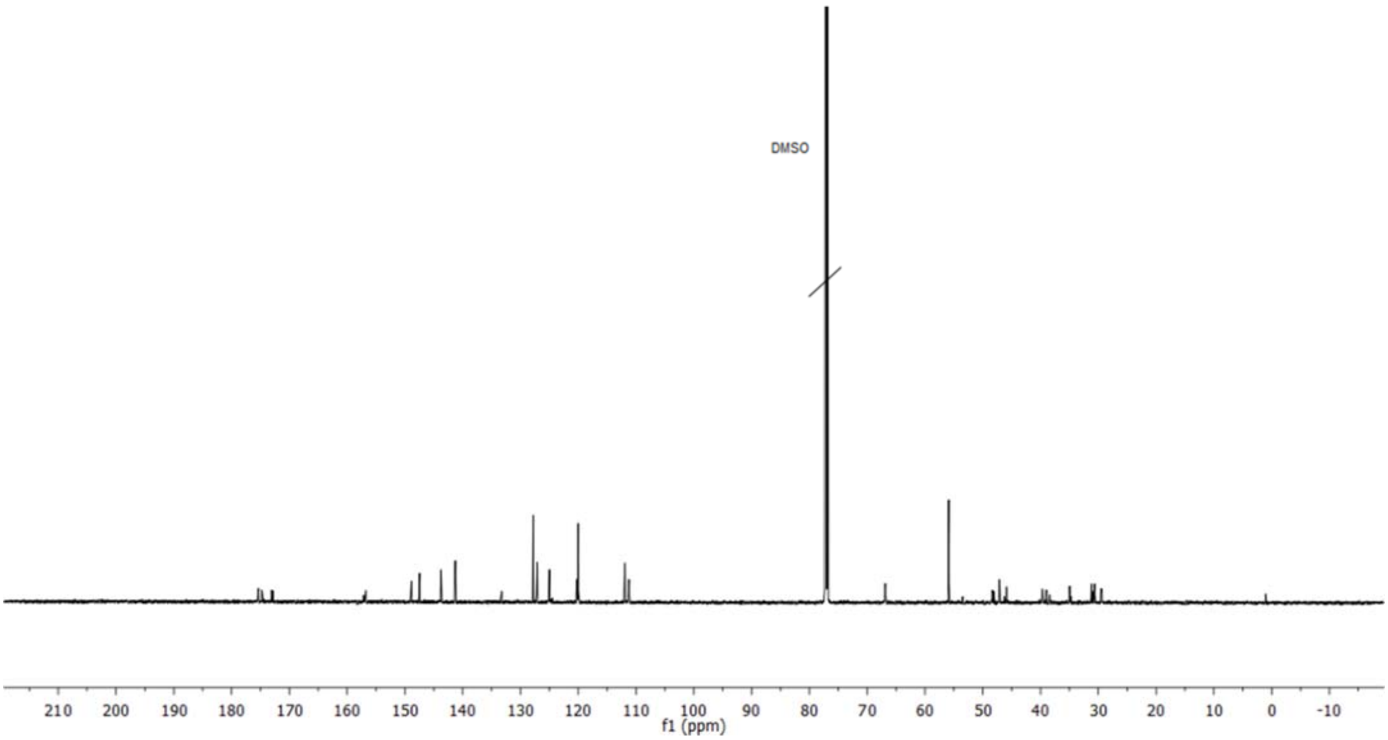

Figure S7. ^{13}C-NMR spectrum of compound **2** (126 MHz, DMSO-d_6).

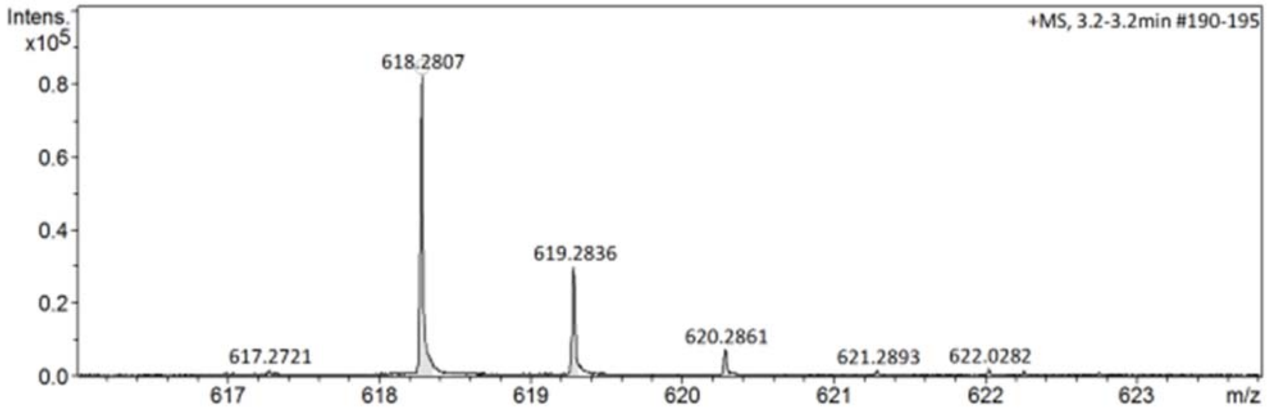

Figure S8. HR-ESI (ESI$^+$ Q-TOF) of compound **2**.

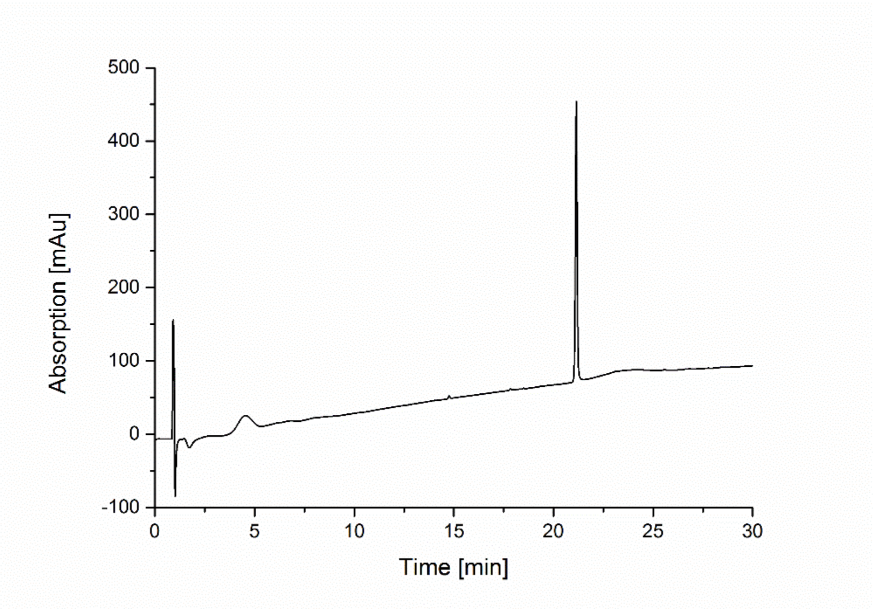

Figure S9. RP-HPLC chromatogram of compound **2** (gradient from 0% to 100% eluent B over 30 min at 25°C).

S4 Oligomer Synthesis and Chemical Analysis

All oligomers were synthesized on solid support according to literature[1] using the building blocks EDS, TrDS and CDS.

On Resin Deprotection Of Trityl

The resin was treated with 0.1 M HCl in trifluorethanol (2x1.5 h). Afterwards the resin was washed with DMF (5x) and the free amines were deprotonated with 20% DIPEA in DMF for 10 minutes.

Side Chain Coupling

After trityl deprotection the resin was treated for 1 h with a solution of 5 eq. acid, 5 eq. PyBOP and 10 eq. DIPEA in DMF. Afterwards the resin was washed with DMF (10x).

Deprotection Of Catechols

All oligomers with a protected catechol moiety were deprotected in solution. For this they were treated with 16 eq. trifluoromethanesulfonic acid and 8 eq. thioanisole per methyl ether in TFA for 16 h. Afterwards the reaction solution was precipitated in diethyl ether and the deprotected oligomers were freeze dried.

Oligomer Chemical Analysis

(**3**) *protected*

Supporting Information

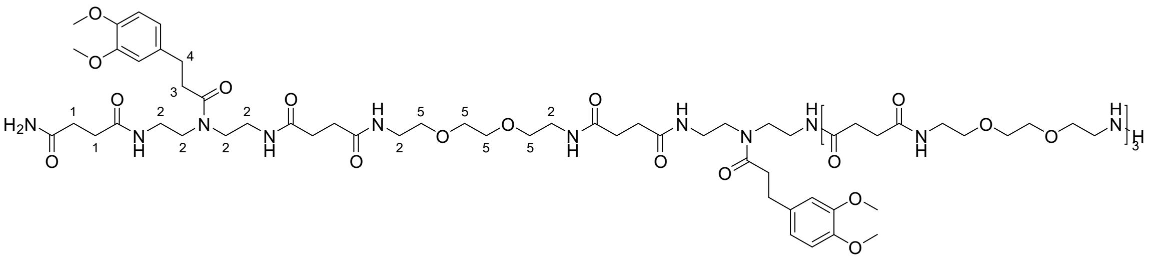

Compound **3 protected** was obtained with a yield of 64% after cleavage from solid support and lyophilization. .

^{1}H-NMR (600 MHz, D_2O) δ [ppm]: 6.90 (m, 4H, $H_{Aromatic}$), 6.79 (d, J = 8.3 Hz, 2H, $H_{Aromatic}$), 6.75 (d, J = 8.0 Hz, 2H, $H_{Aromatic}$), 3.80 (s, 6H, OCH_3), 3.78 (s, 6H, OCH_3) 3.74-3.54 (m, 32H, H-5), 3.40-3.17 (m, 32H, H-2), 2.82 (m, 4H, H-4), 2.64 (m, 4H, H-3), 2.51-2.36 (m, 24H, H-1).

^{13}C-NMR (126 MHz, D_2O) δ [ppm]: 178.36, 178.23, 176.63, 175.73, 175.68, 175.64, 175.59, 175.55, 175.53, 175.51, 175.49, 175.38, 175.36, 149.04, 147.63, 134.91, 121.82, 113.12, 112.81, 70.55, 70.42, 69.82, 67.36, 56.61, 56.56, 48.05, 45.86, 45.77, 40.09, 39.89, 39.82, 38.29, 37.99, 35.20, 35.14, 31.97, 31.93, 31.90, 31.84, 31.76, 31.70, 31.67, 31.58, 31.17, 31.03.

HR-ESI-MS: calculated mass for $C_{78}H_{132}N_{15}O_{26}$ $[M+3H]^{3+}$ 564.9817, found 564.9825.

RP-HPLC (gradient from 0% to 50% eluent B over 30 min at 25°C):t_r=14.0 min, purity 92%.

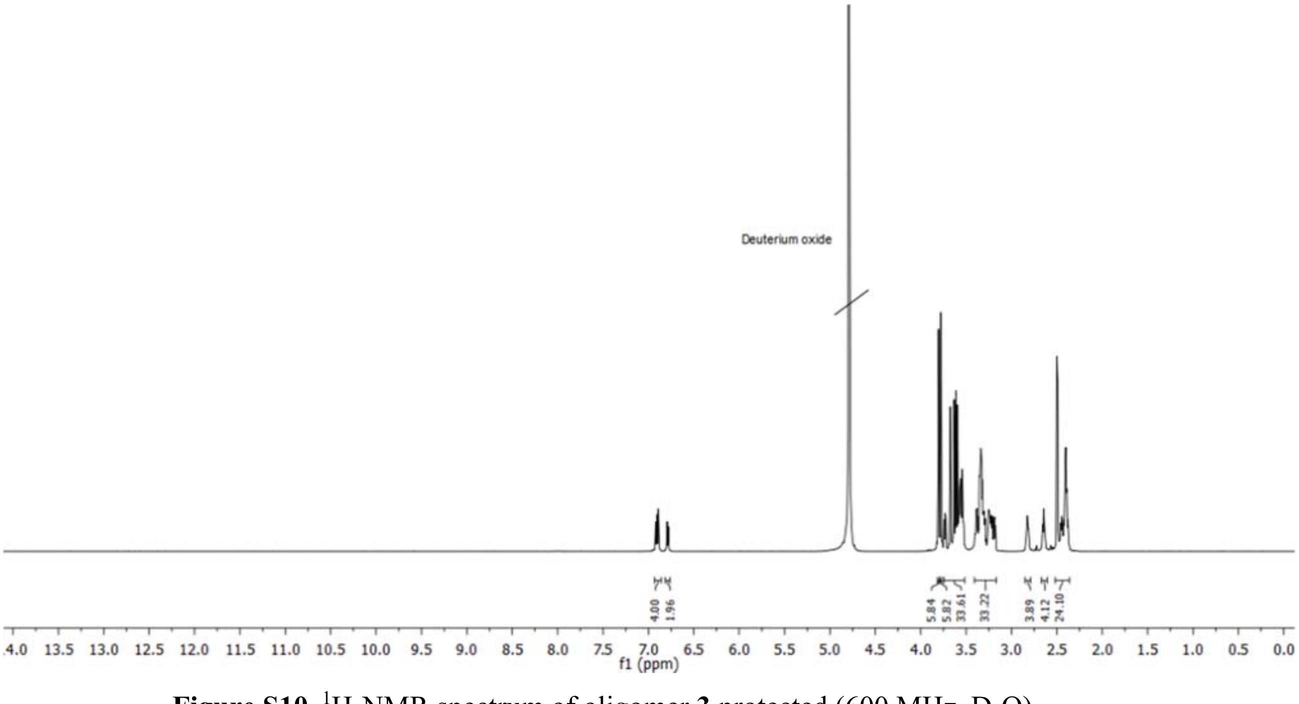

Figure S10. ^{1}H-NMR spectrum of oligomer **3** protected (600 MHz, D_2O).

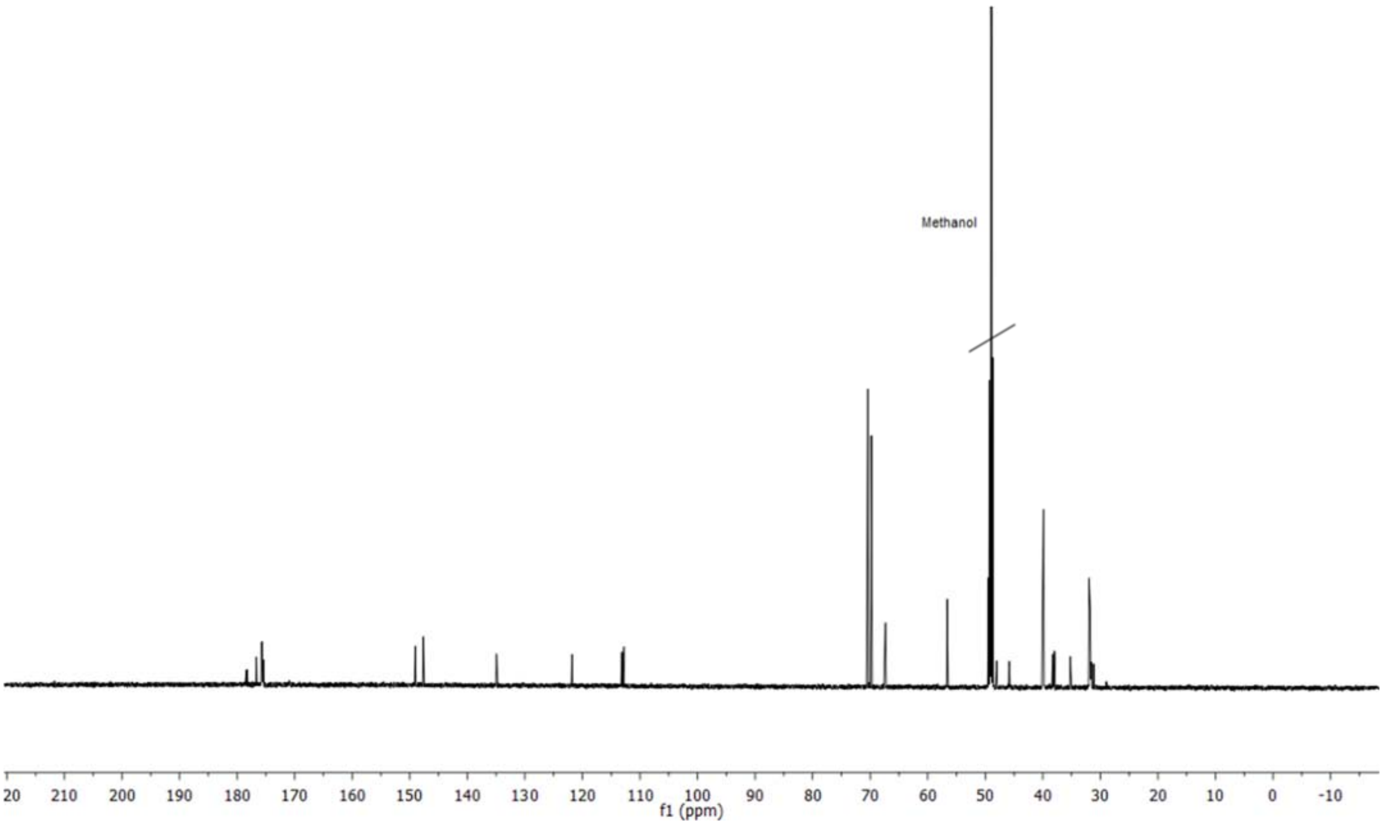

Figure S11. ^{13}C-NMR spectrum of oligomer **3** protected (126 MHz, D_2O).

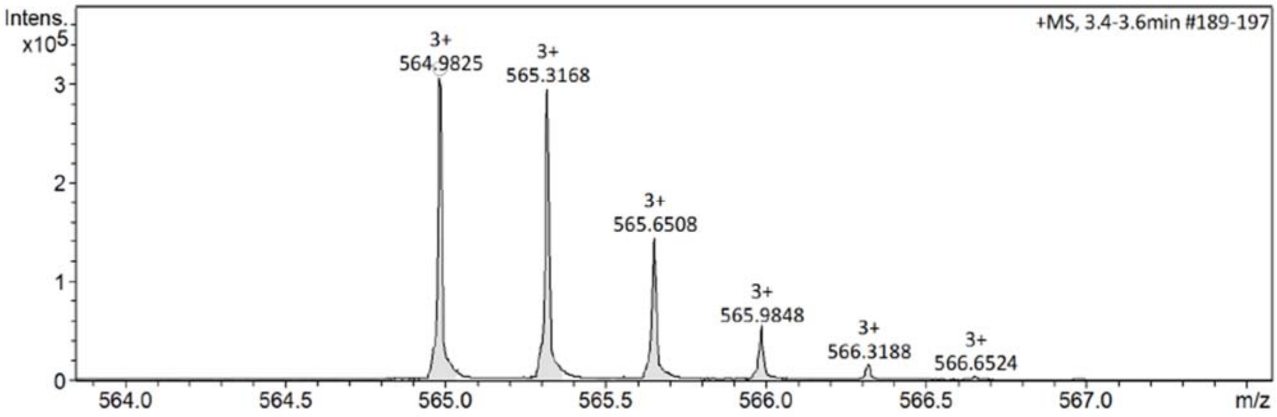

Figure S12. HR-ESI (ESI$^+$ Q-TOF) of oligomer **3** protected.

Supporting Information

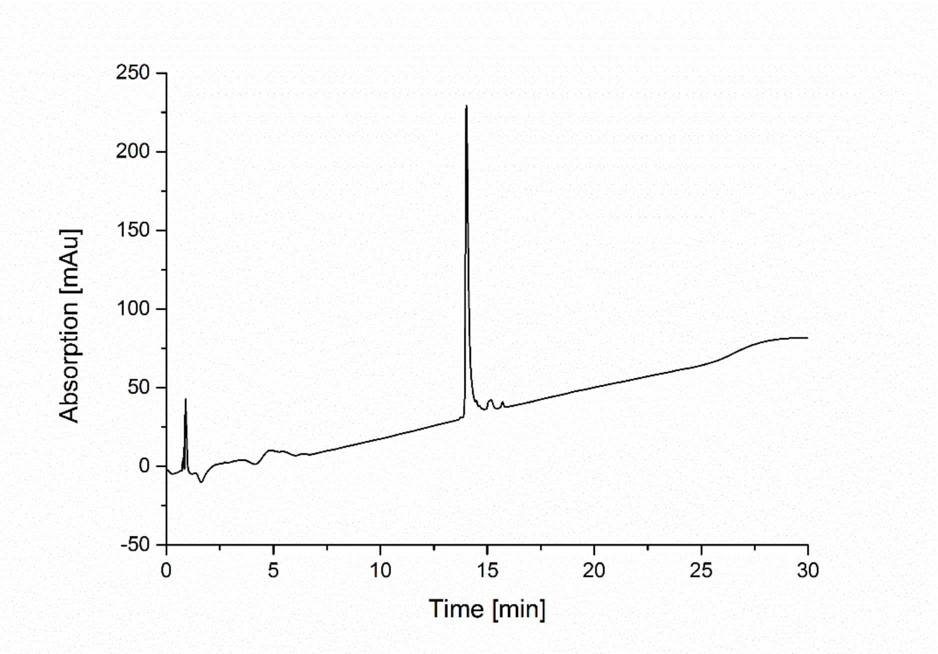

Figure S13. RP-HPLC chromatogram of compound **3** protected (gradient from 0% to 50% eluent B over 30 min at 25°C).

(**3**)

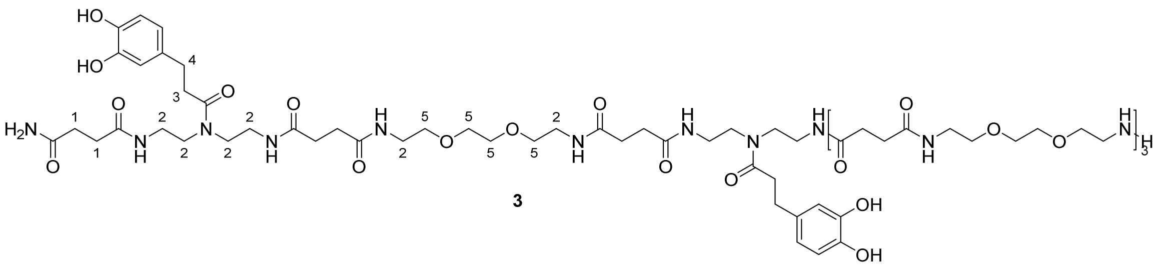

3

Compound **3** was obtained with a yield of 32% after deprotection, purification by preparative RP-HPLC and lyophilization.

^{1}H-NMR (500 MHz, D_2O) δ [ppm]: 8.32 (s, NH), 6.91 (dd, J = 8.0 Hz, 1.8 Hz, 2H, $H_{Aromatic}$), 6.85 (s, 2H, $H_{Aromatic}$), 6.75 (d, J = 8.0 Hz, 2H, $H_{Aromatic}$), 3.86-3.64 (m, 32H, H-5), 3.52-3.27 (m, 32H, H-2), 2.85 (m, 4H, H-4), 2.71 (m, 4H, H-3), 2.62-2.48 (m, 24H, H-1).

^{13}C-NMR (126 MHz, D_2O) δ [ppm]: 176.46, 176.39, 176.37, 175.40, 175.37, 175.29, 175.20, 175.09, 144.54, 144.48, 142.86, 134.10, 134.07, 121.17, 121.13, 118.92, 116.82, 116.78, 116.70, 70.12, 69.97, 69.37, 66.90, 45.41, 45.33, 39.67, 39.47, 39.40, 37.88, 37.62, 34.79, 31.61, 31.57, 31.53, 31.48, 31.42, 31.38, 31.23, 30.96, 30.79, 30.69.

HR-ESI-MS: calculated mass for $C_{78}H_{132}N_{15}O_{26}$ $[M+3H]^{3+}$ 564.9817, found 564.9825.

RP-HPLC (gradient from 0% to 50% eluent B over 30 min at 25°C):t_r=12.3 min, purity 93%.

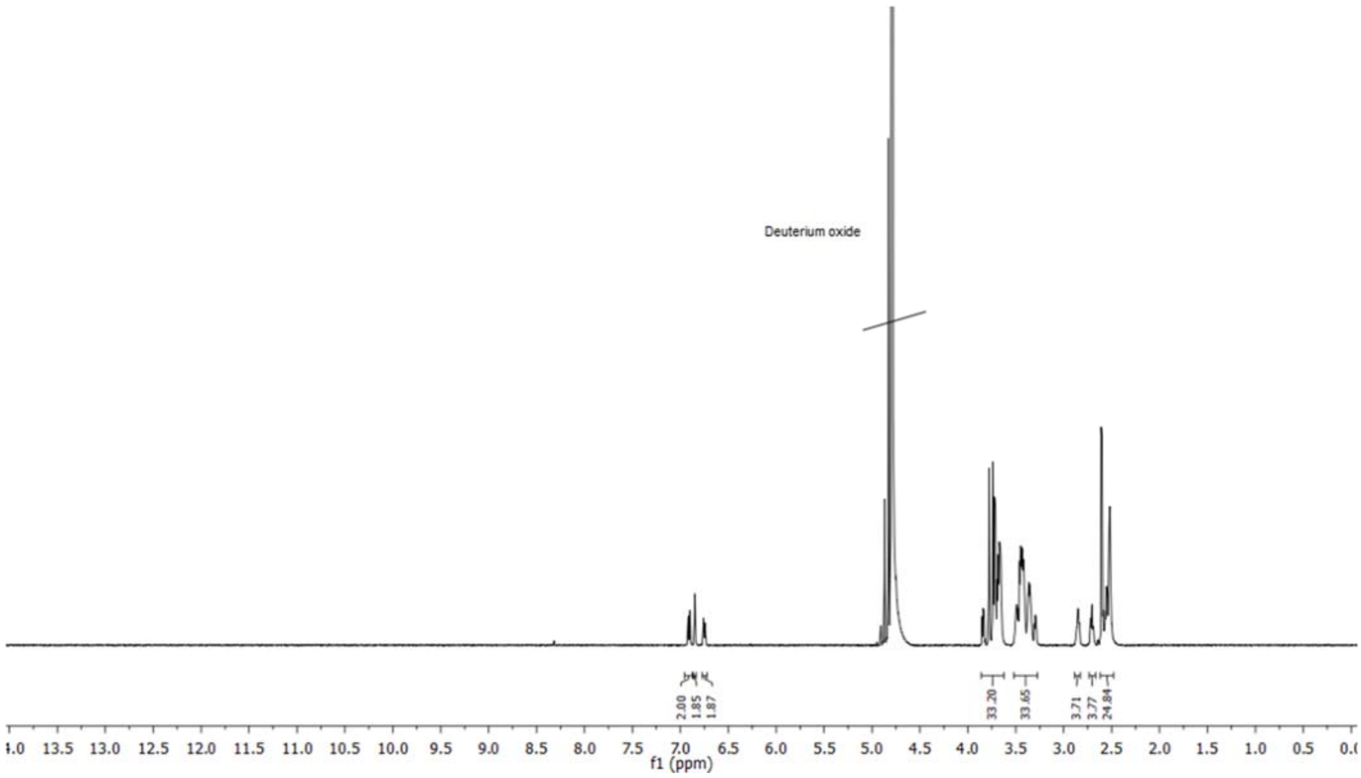

Figure S14. ^{1}H-NMR spectrum of compound **3** (500 MHz, D_2O).

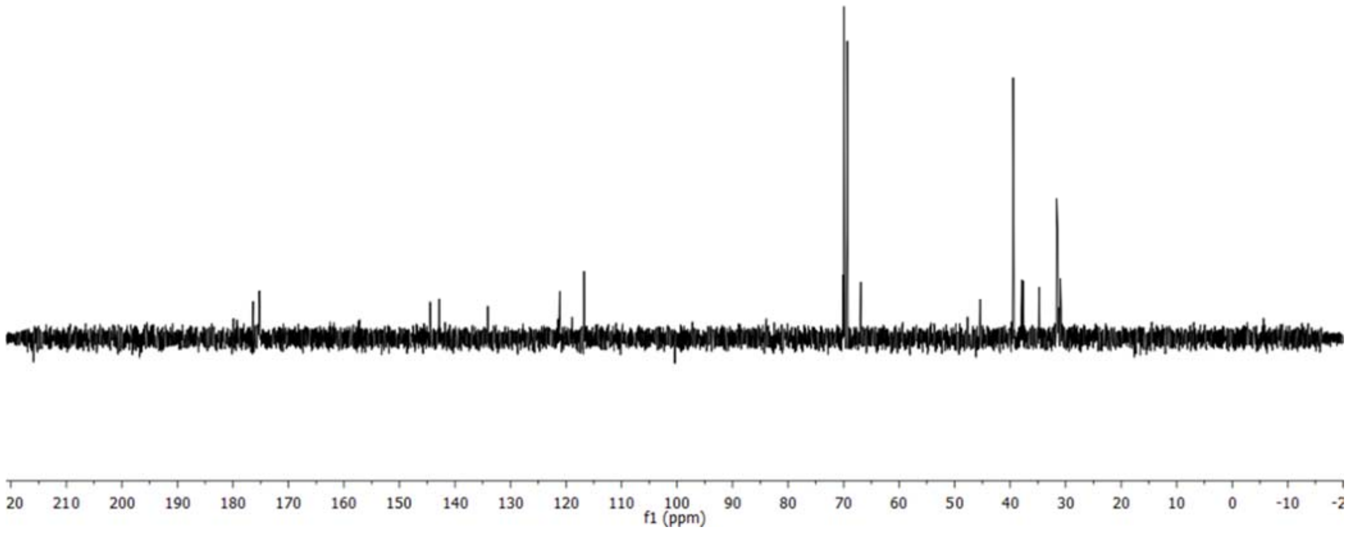

Figure S15. ^{13}C-NMR spectrum of compound **3** (126 MHz, D_2O).

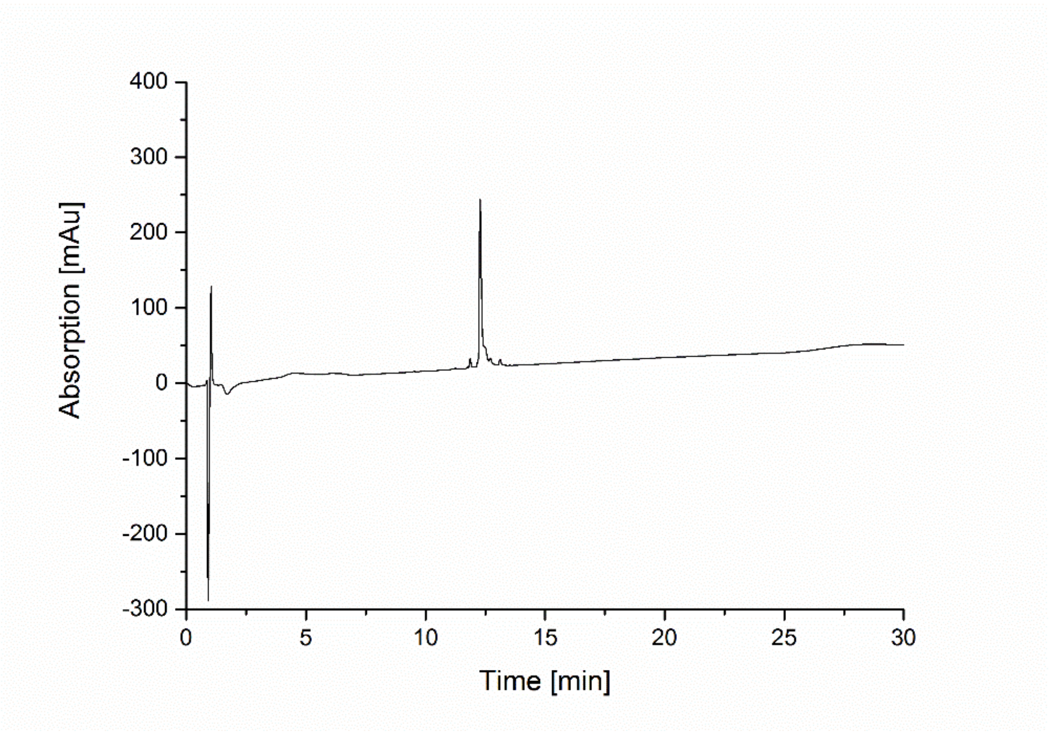

Figure S16. RP-HPLC chromatogram of compound **3** (gradient from 0% to 50% eluent B over 30 min at 25°C).

(**4**)

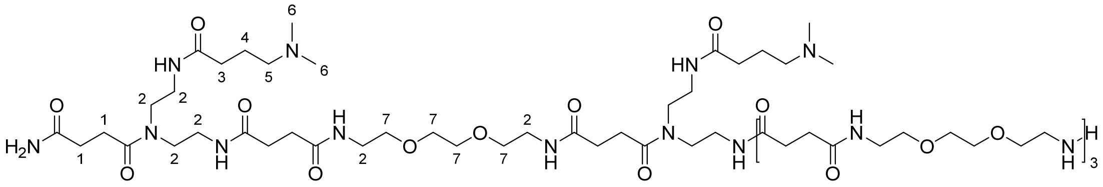

4

Compound **4** was obtained with a yield of 45% after purification by preparative RP-HPLC and lyophilization.^{1}H-NMR (500 MHz, D_2O) δ [ppm]: 8.47 (s, NH), 3.79-3.58 (m, 32H, H-7), 3.57-3.28 (m, 32H, H-2), 3.25-3.10 (m, 4H, H-5), 2.89 (s, 12H, H-6), 2.80 (m, 2H, H-8), 2.69 (m, 4H, H-3), 2.60-2.30 (m, 24H, H-1), 2.98 (m, 4H, 4-H).

^{13}C-NMR (126 MHz, D_2O) δ [ppm]: 175.85, 175.79, 175.65, 175.60, 175.56, 171.71, 70.42, 69.81, 57.96, 43.63, 39.87, 37.90, 31.98, 31.91, 31.84, 21.01, 20.98.

HR-ESI-MS: calculated mass for $C_{68}H_{130}N_{17}O_{22}$ $[M+3H]^{3+}$ 512.3187, found 512.3183.

RP-HPLC (gradient from 0% to 50% eluent B over 30 min at 25°C):t_r=7.1 min, purity 99%.

Supporting Information

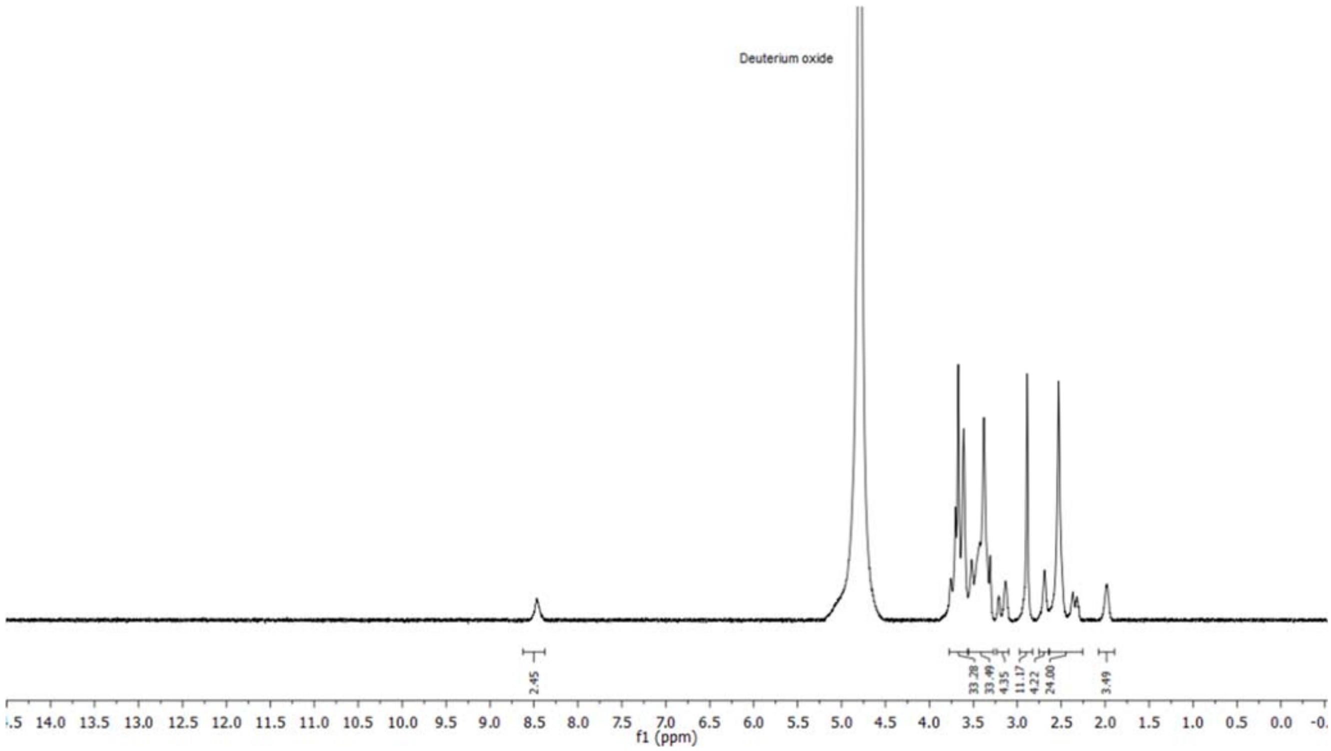

Figure S17. ^{1}H-NMR spectrum of compound **4** (500 MHz, D_2O).

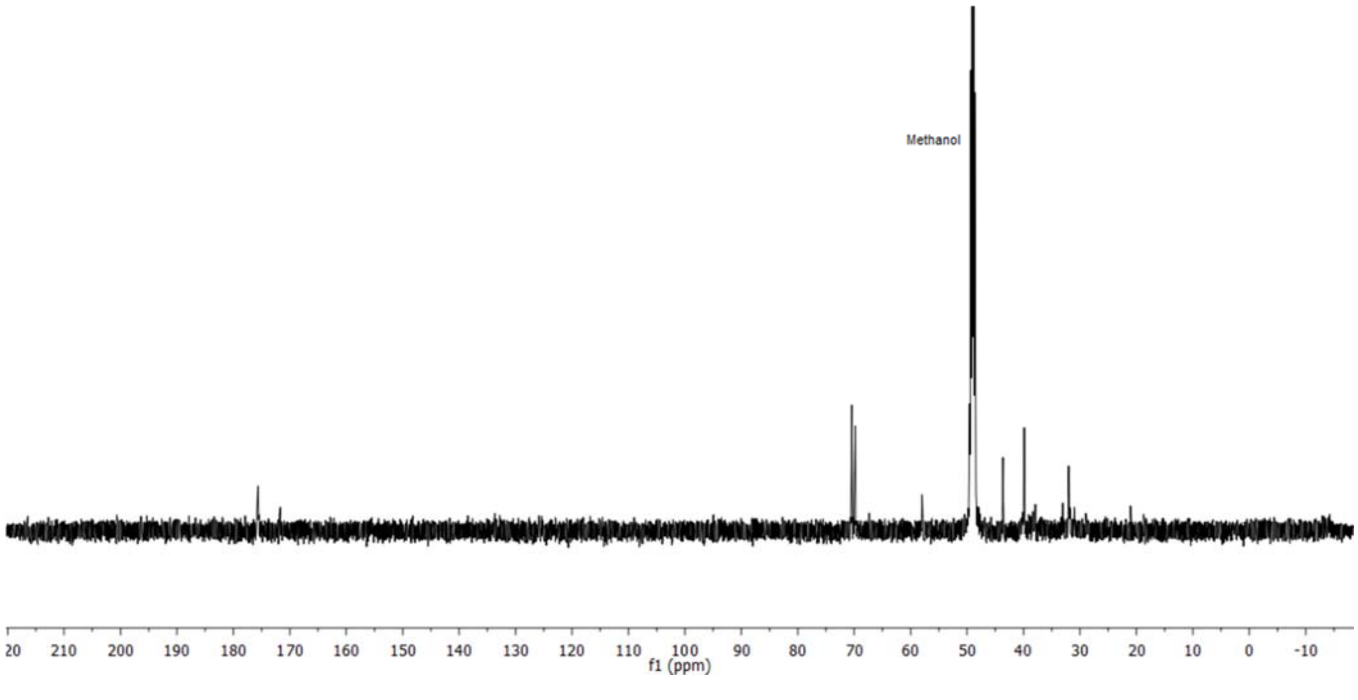

Figure S18. ^{13}C-NMR spectrum of compound **4** (126 MHz, D_2O).

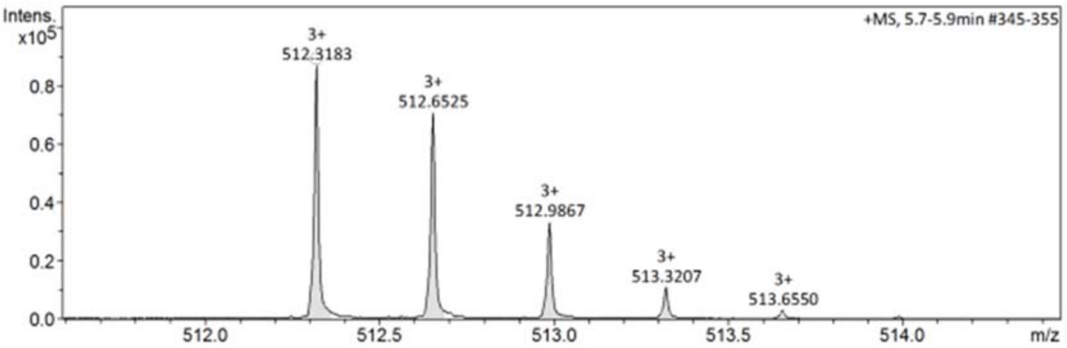

Figure S19. HR-ESI (ESI^+ Q-TOF) of compound **4**.

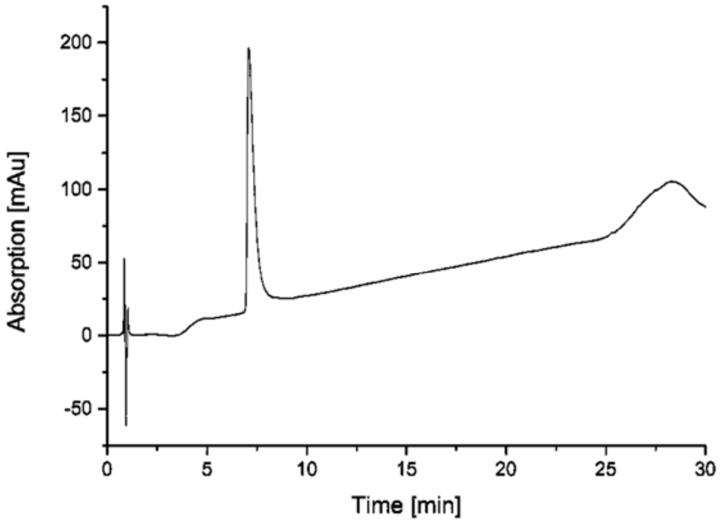

Figure S20. RP-HPLC chromatogram of compound **4** (gradient from 0% to 50% eluent B over 30 min at 25°C).

(**5**)

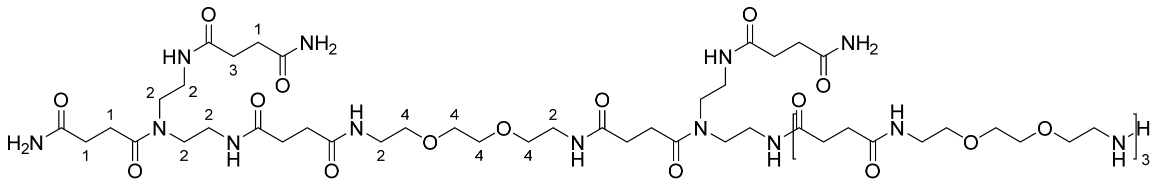

5

Compound **5** was obtained with a yield of 35% after purification by preparative RP-HPLC and lyophilization.

^{1}H-NMR (500 MHz, D_2O) δ [ppm]: 8.49 (s, NH), 3.80-3.59 (m, 32H, H-4), 3.57-3.20 (m, 32H, H-2), 2.71 (m, 4H, H-3) 2.60-2.46 (m, 28H, H-1).

^{13}C-NMR (126 MHz, D_2O) δ [ppm]: 175.73, 175.66, 175.63, 175.58, 175.53, 175.50, 175.47, 108.36, 108.21, 100.86, 70.54, 70.40, 69.80, 39.87, 39.80, 32.00, 31.95, 31.91, 31.87, 31.83, 31.79, 31.70, 31.67, 31.64, 28.94, 28.68.

HR-ESI-MS: calculated mass for $C_{64}H_{118}N_{17}O_{24}$ $[M+3H]^{3+}$ 502.9506, found 502.9499.

RP-HPLC (gradient from 0% to 50% eluent B over 30 min at 25°C):t_r=8.3 min, purity 94%.

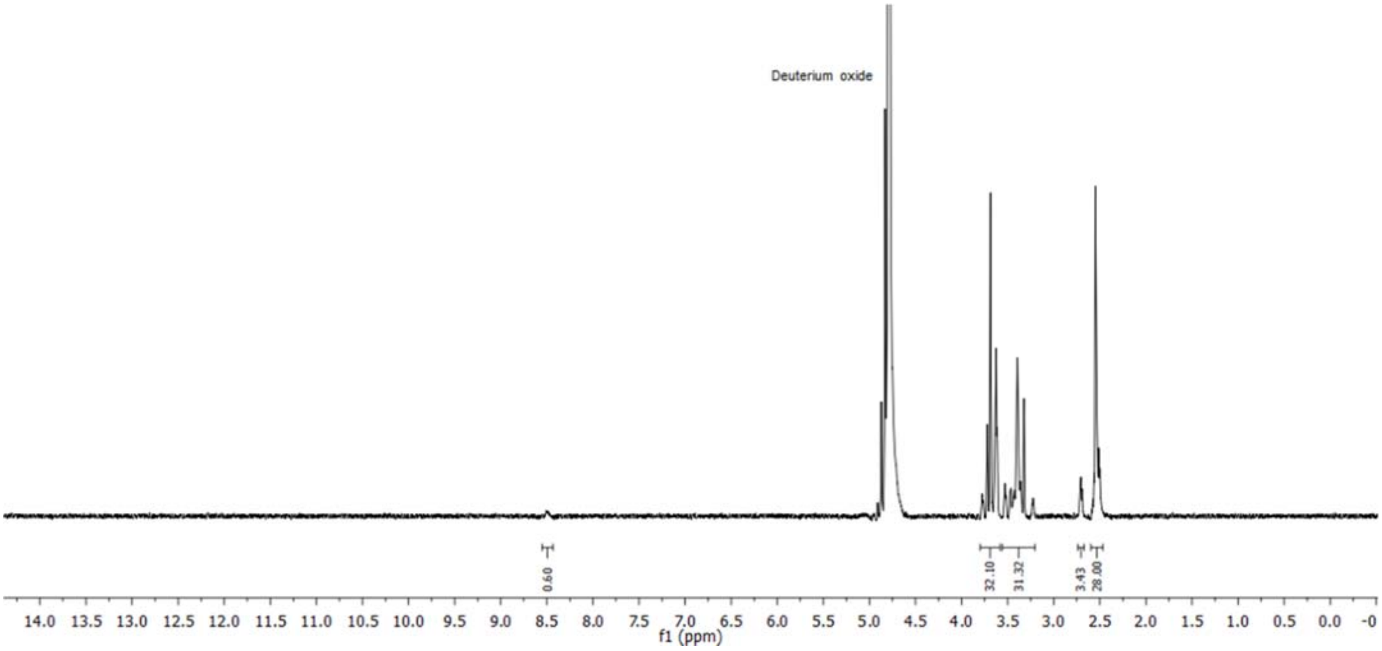

Figure S21. ^1H-NMR spectrum of compound **5** (500 MHz, D_2O).

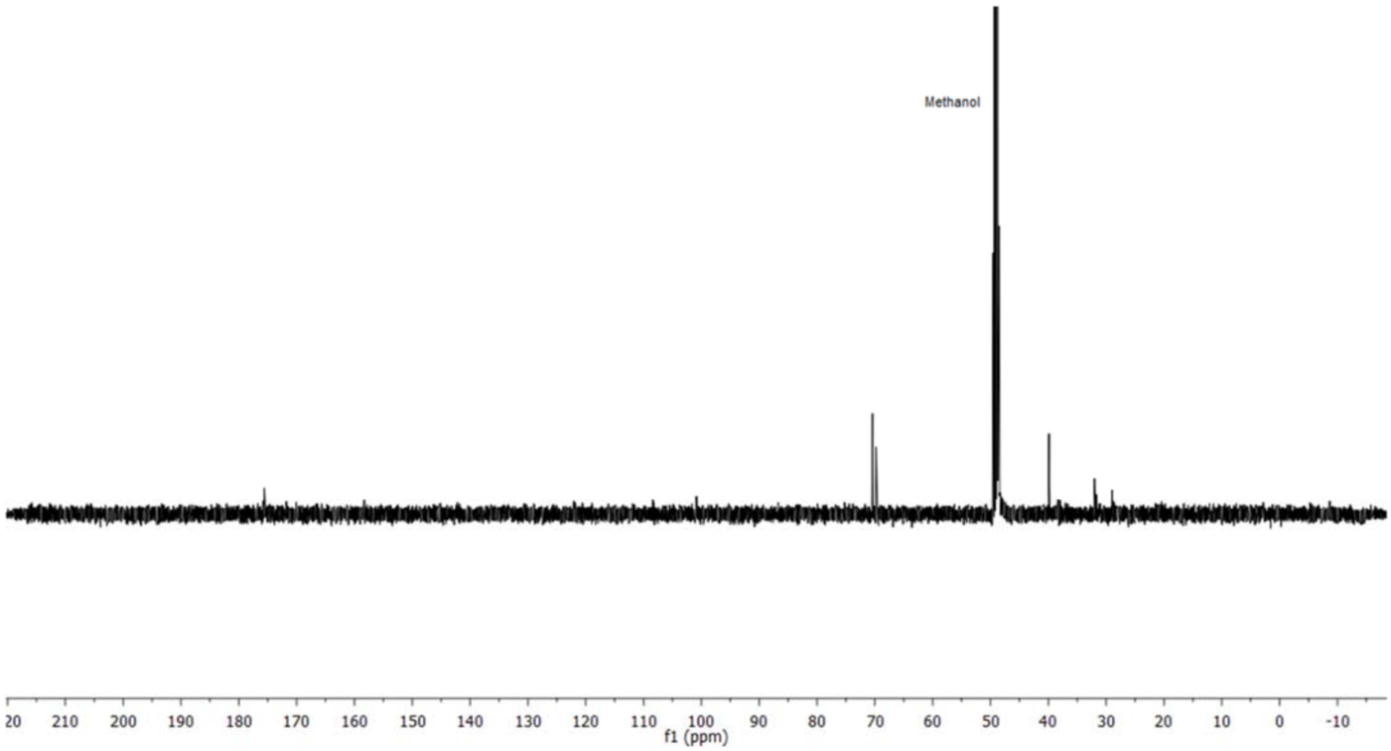

Figure S22. ^{13}C-NMR spectrum of compound **5** (126 MHz, D_2O).

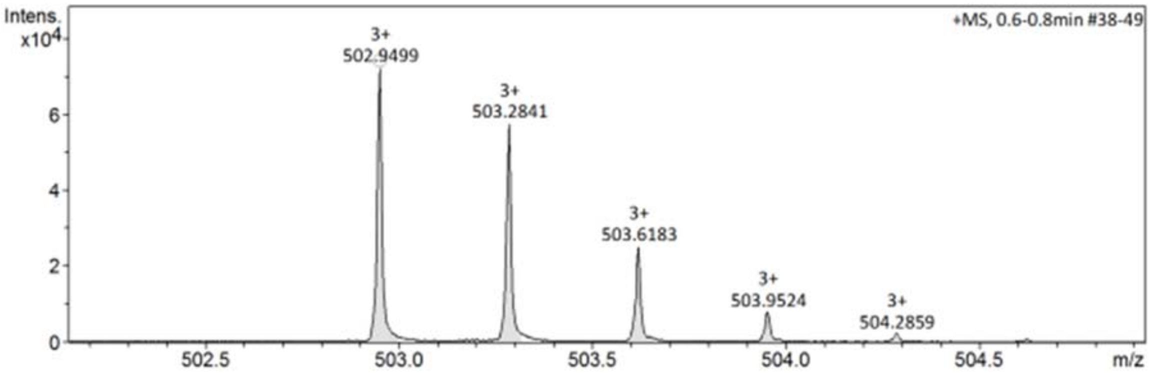

Figure S23. HR-ESI (ESI^+ Q-TOF) of compound **5**.

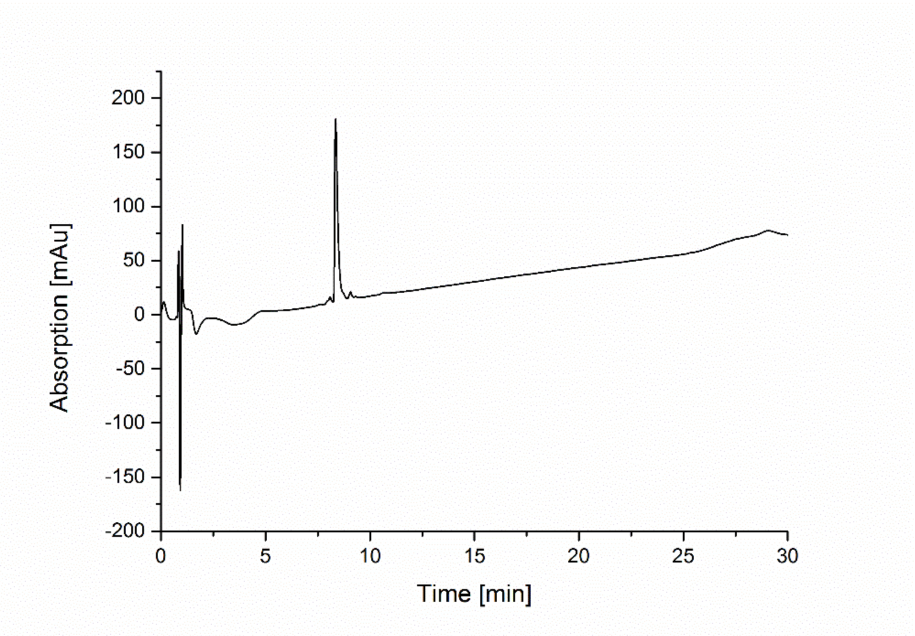

Figure S24. RP-HPLC chromatogram of compound **5** (gradient from 0% to 50% eluent B over 30 min at 25°C).

(**6**) *protected*

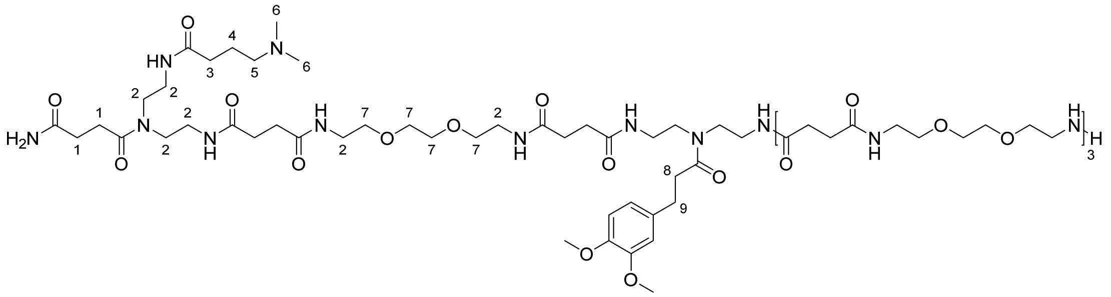

Compound **6** protected was obtained with a yield of 72% after cleavage from solid support and lyophilization.

^{1}H-NMR (500 MHz, D_2O) δ [ppm]: 6.92 (m, 2H, $H_{Aromatic}$), 6.81 (m, 1H, $H_{Aromatic}$), 3.81 (s, 3H, OCH_3), 3.79 (s, 3H, OCH_3), 3.74-3.51 (m, 32H, H-7), 3.50-3.15 (m, 32H, H-2), 3.09 (m, 2H, H-5), 2.85 (m, 8H, 6-H, 9-H), 2.64 (m, 4H, 3-H, 8-H), 2.53-2.25 (m, 24H, 1-H), 1.94 (m, 2H, 4-H).

^{13}C-NMR (126 MHz, D_2O) δ [ppm]: 178.60, 176.60, 175.81, 175.67, 175.61, 175.58, 175.50, 175.46, 175.35, 149.15, 147.73, 134.99, 130.73, 121.84, 120.71, 118.39, 116.08, 115.65, 113.76, 113.33, 113.05, 70.55, 70.39, 69.78, 67.29, 66.81, 57.95, 56.75, 56.67, 48.06, 47.95, 47.77, 45.91, 45.81, 43.62, 40.09, 39.87, 39.25, 39.07, 38.91, 38.31, 38.17, 38.00, 37.90, 35.17, 33.02, 32.90, 31.99, 31.86, 31.75, 31.62, 30.96, 28.94, 28.85, 20.99, 20.94, 15.04.

HR-ESI-MS: calculated mass for $C_{73}H_{131}N_{16}O_{24}$ $[M+3H]^{3+}$ 538.6502, found 538.6499.

RP-HPLC (gradient from 0% to 50% eluent B over 30 min at 25°C):t_r=10.6 min, purity 87%.

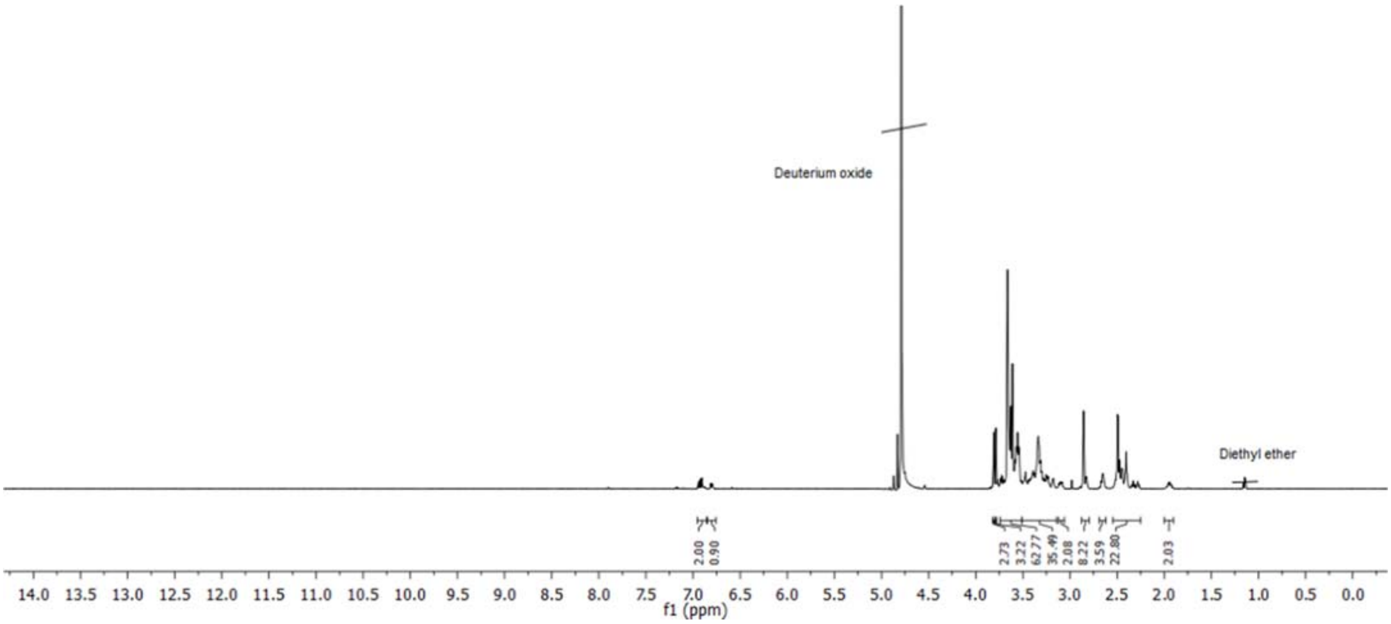

Figure S25. ^{1}H-NMR spectrum of compound **6** protected (500 MHz, D_2O).

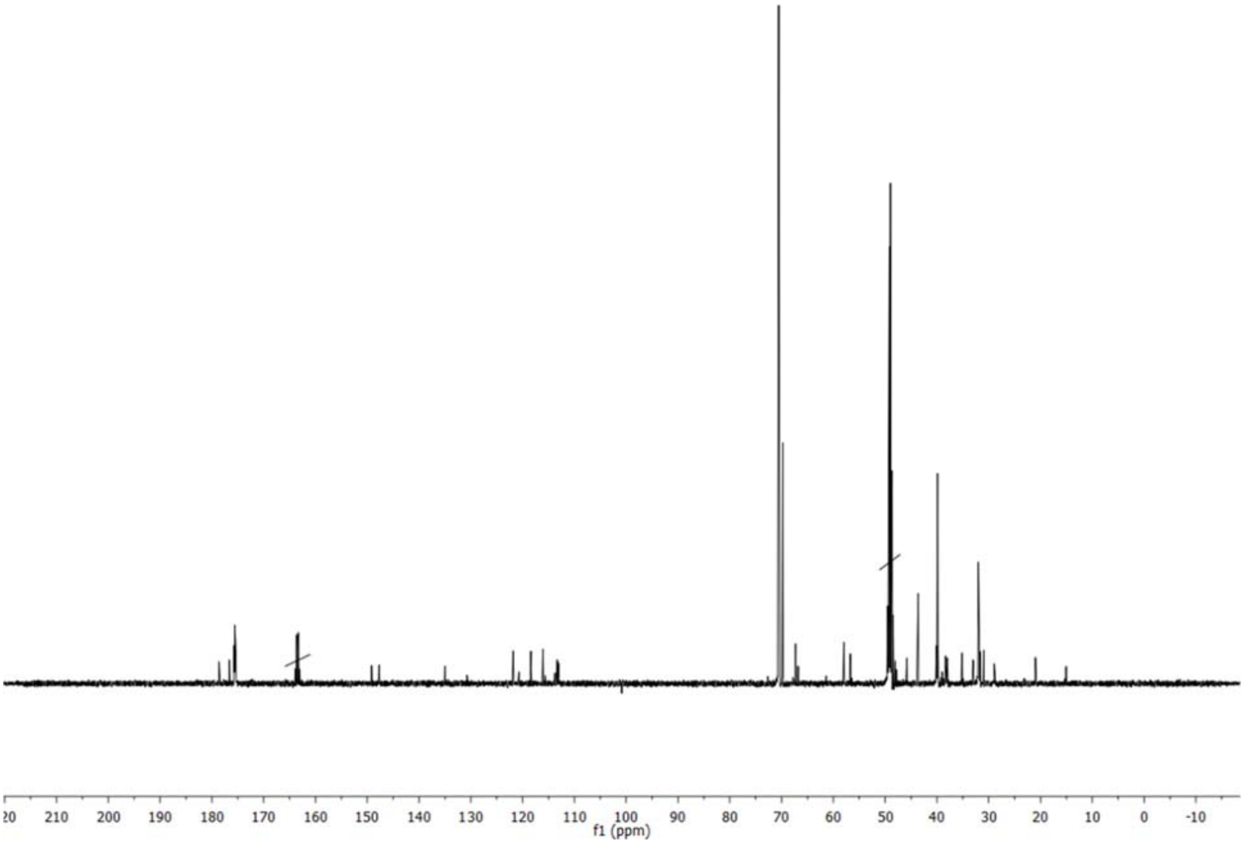

Figure S26. ^{13}C-NMR spectrum of compound **6** protected (126 MHz, D_2O).

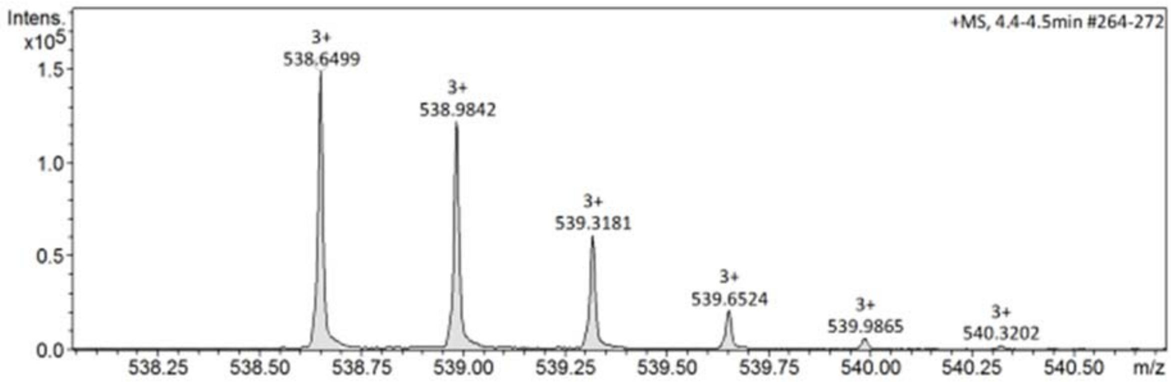

Figure S27. HR-ESI (ESI^+ Q-TOF) of compound **6** protected.

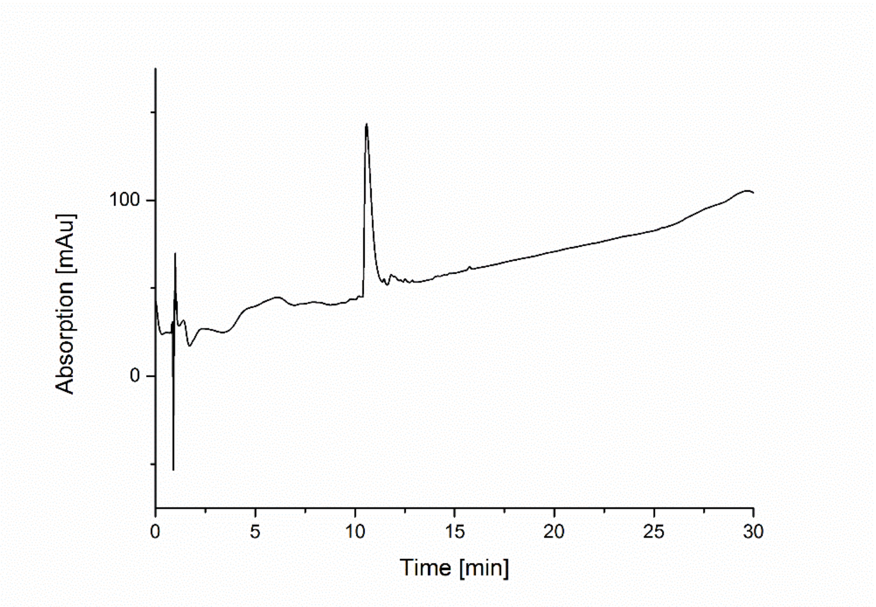

Figure S28. RP-HPLC chromatogram of compound **6** protected (gradient from 0% to 50% eluent B over 30 min at 25°C).

(**6**)

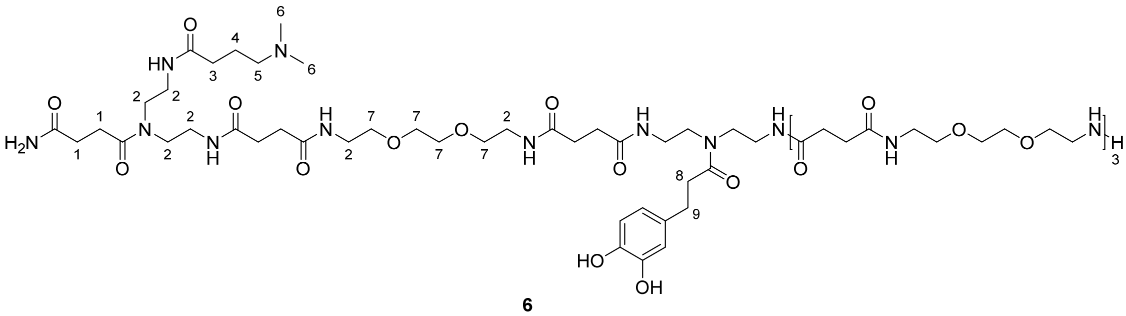

6

Compound **6** was obtained with a yield of 26% after deprotection, purification by preparative RP-HPLC and lyophilization.

^{1}H-NMR (500 MHz, D_2O) δ [ppm]: 6.84 (d, J = 8.0 Hz, 1H, $H_{Aromatic}$), 6.78 (s, 1H, $H_{Aromatic}$), 6.69 (d, J = 7.5 Hz1H, $H_{Aromatic}$), 3.77-3.55 (m, 32H, H-7), 3.53-3.20 (m, 32H, H-2), 3.12 (m, 2H, H-5), 2.88 (m,

6H, H-6,), 2.78 (t, J = 7.3 Hz, 2H, H-9), 2.68 (t, J = 6.7 Hz, 2H, H-3), 2.64 (t, J = 7.3 Hz, 2H, H-8), 2.56-2.30 (m, 24H, H-1), 1.97 (m, 2H, H-4).

^{13}C-NMR (126 MHz, D_2O) δ [ppm]: 180.71, 178.76, 177.92, 177.82, 177.77, 177.72, 177.69, 177.64, 177.62, 177.59, 177.48, 123.52, 119.08, 72.47, 72.34, 71.74, 69.30, 59.83, 50.01, 49.88, 49.68, 47.79, 47.71, 47.64, 45.58, 42.00, 41.82, 41.76, 40.18, 40.06, 39.92, 39.82, 39.80, 37.20, 34.98, 34.85, 33.93, 33.89, 33.86, 33.79, 33.73, 33.68, 33.36, 32.88, 30.90, 30.81, 23.00, 22.94.

HR-ESI-MS: calculated mass for $C_{71}H_{127}N_{16}O_{24}$ $[M+3H]^{3+}$ 529.3064, found 529.3067.

RP-HPLC (gradient from 0% to 50% eluent B over 30 min at 25°C):t_r=8.3 min, purity 97%.

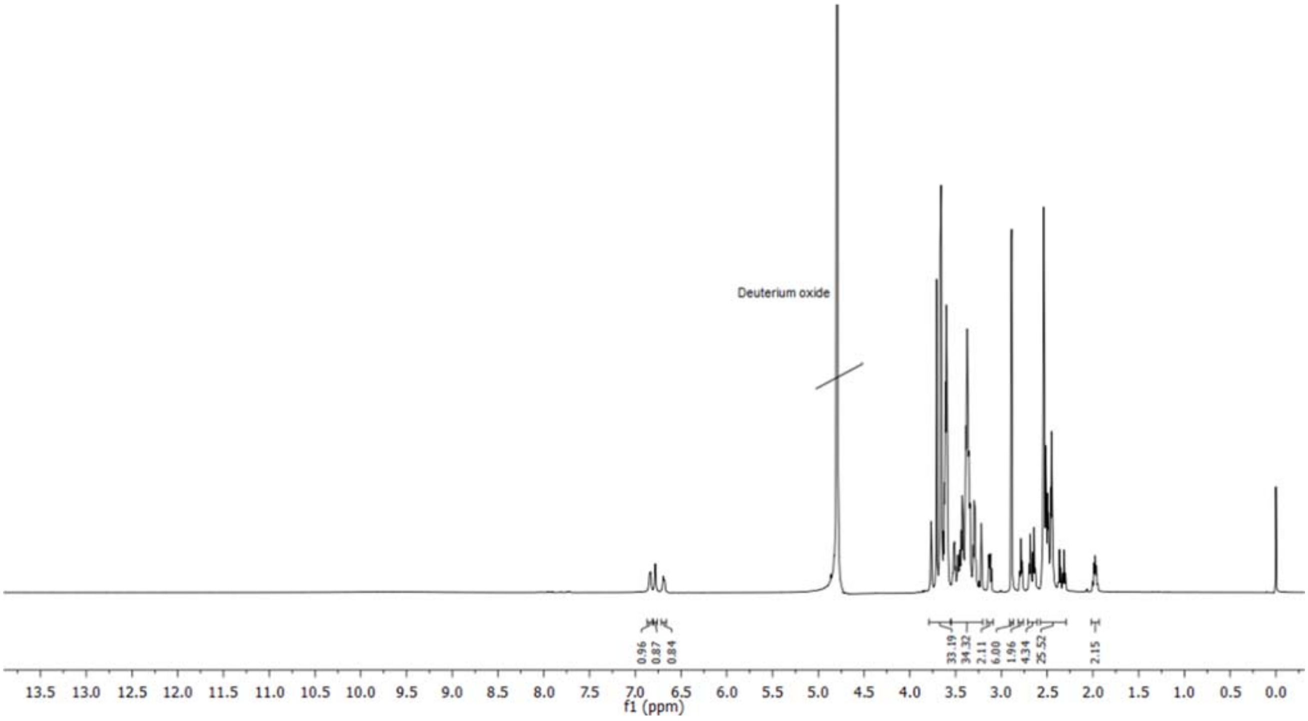

Figure S29. ^{1}H-NMR spectrum of compound **6** (600 MHz, D_2O).

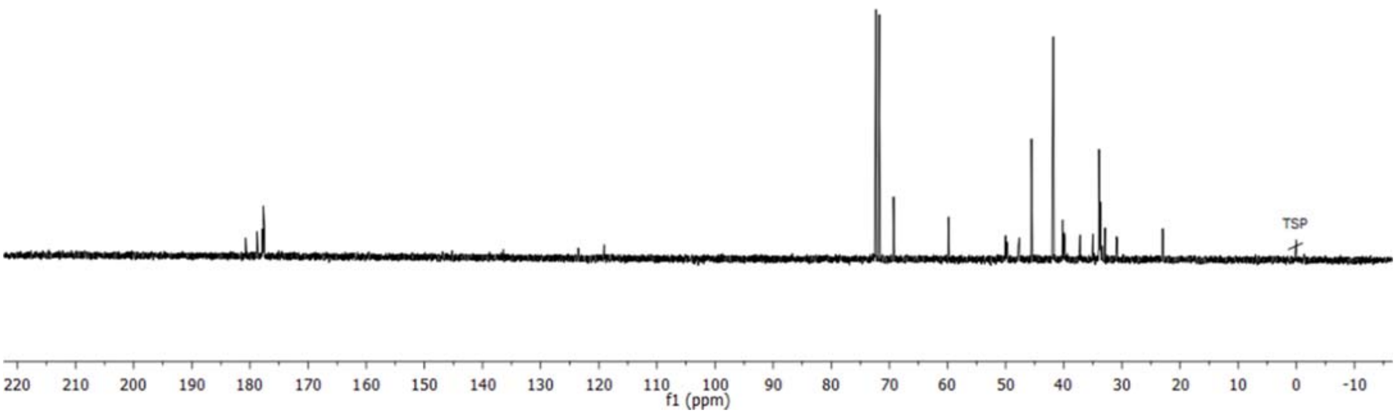

Figure S30. ^{13}C-NMR spectrum of compound **6** (126 MHz, D_2O).

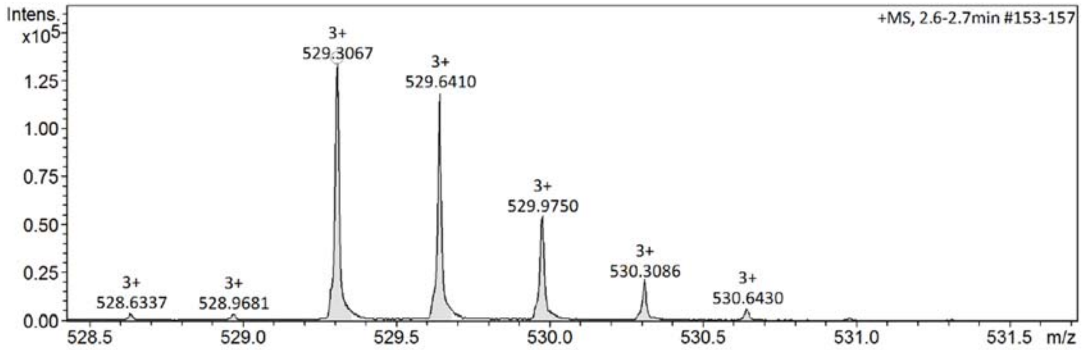

Figure S31. HR-ESI (ESI$^+$ Q-TOF) of compound **6**.

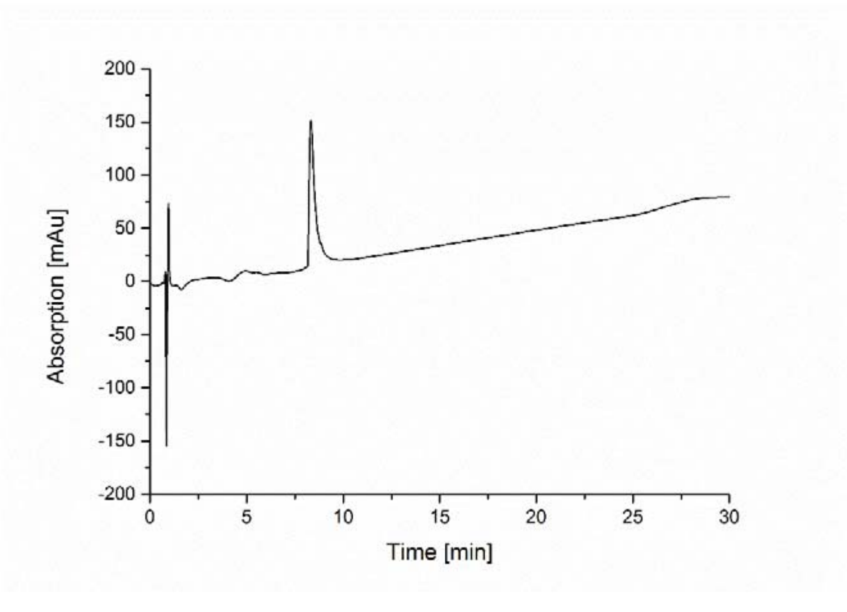

Figure S32. RP-HPLC chromatogram of compound **6** (gradient from 0% to 50% eluent B over 30 min at 25°C).

Supporting Information

(7) *protected*

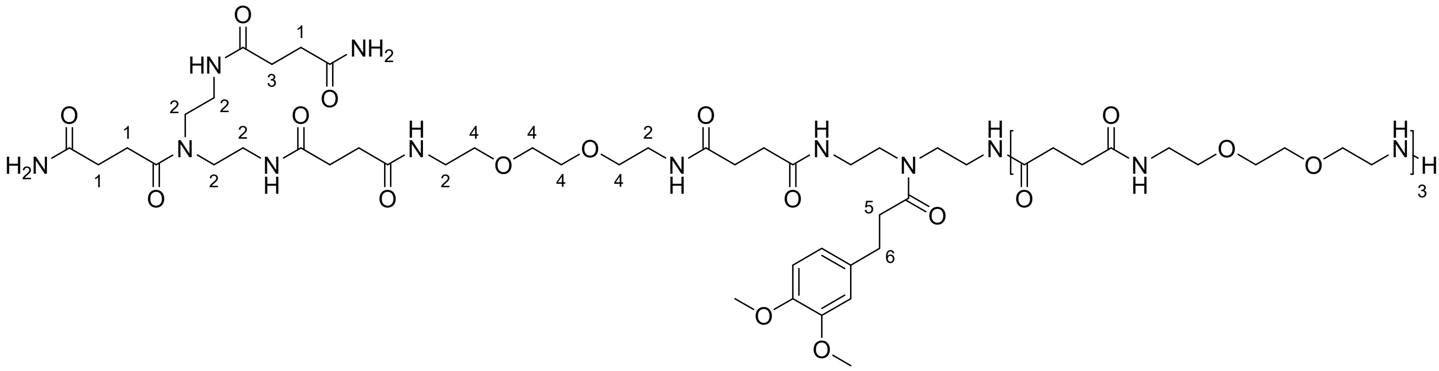

7 protected was obtained with a yield of 52% after cleavage from solid support and lyophilization.

^{1}H-NMR (500 MHz, D_2O) δ [ppm]: 8.49 (s, NH), 6.98 (d, J = 8.2 Hz, 1H, $H_{Aromatic}$), 6.95 (d, J = 2.0 Hz, 1H, $H_{Aromatic}$), 6.85 (d, J = 8.2 Hz, 2.0 Hz, 1H, $H_{Aromatic}$), 3.85 (s, 3H, OCH_3), 3.83 (s, 3H, OCH_3) 3.77-3.49 (m, 32H, H-4), 3.47-3.20 (m, 32H, H-2), 2.87 (t, J = 7.3 Hz, 2H, H-6), 2.69 (m, 4H, H-3, H-5), 2.62-2.45 (m, 26H, H-1).

^{13}C-NMR (126 MHz, D_2O) δ [ppm]: 178.64, 176.60, 175.67, 175.65, 175.59, 175.56, 175.52, 175.49, 175.45, 175.33, 171.61, 149.16, 147.75, 134.99, 121.84, 113.29, 113.00, 70.57, 70.43, 69.82, 67.33, 56.72, 56.65, 48.07, 46.06, 45.81, 40.11, 39.88, 39.82, 38.31, 38.00, 37.94, 35.21, 31.99, 31.96, 31.92, 31.86, 31.80, 31.74, 31.71, 31.66, 31.24, 31.14, 31.01, 28.93.

HR-ESI-MS: calculated mass for $C_{71}H_{125}N_{16}O_{25}$ $[M+3H]^{3+}$ 533.9662, found 533.9665.

RP-HPLC (gradient from 0% to 50% eluent B over 30 min at 25°C):t_r=10.7 min, purity 97%.

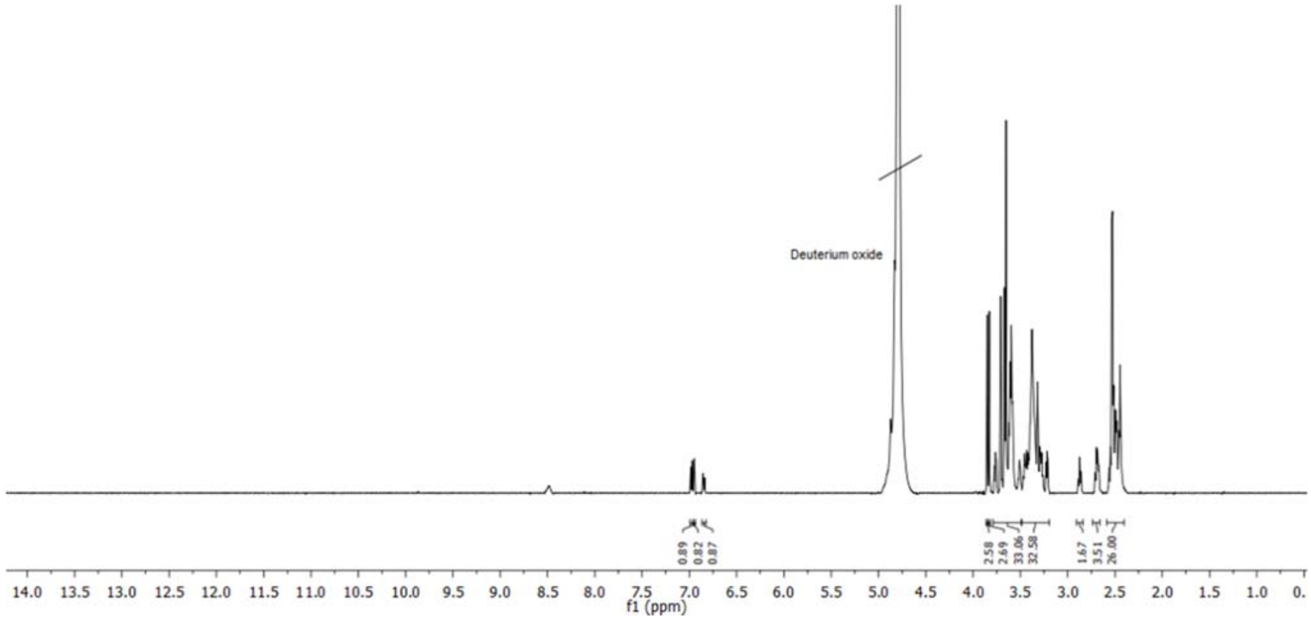

Figure S33. ^{1}H-NMR spectrum of compound **7** protected (600 MHz, D_2O).

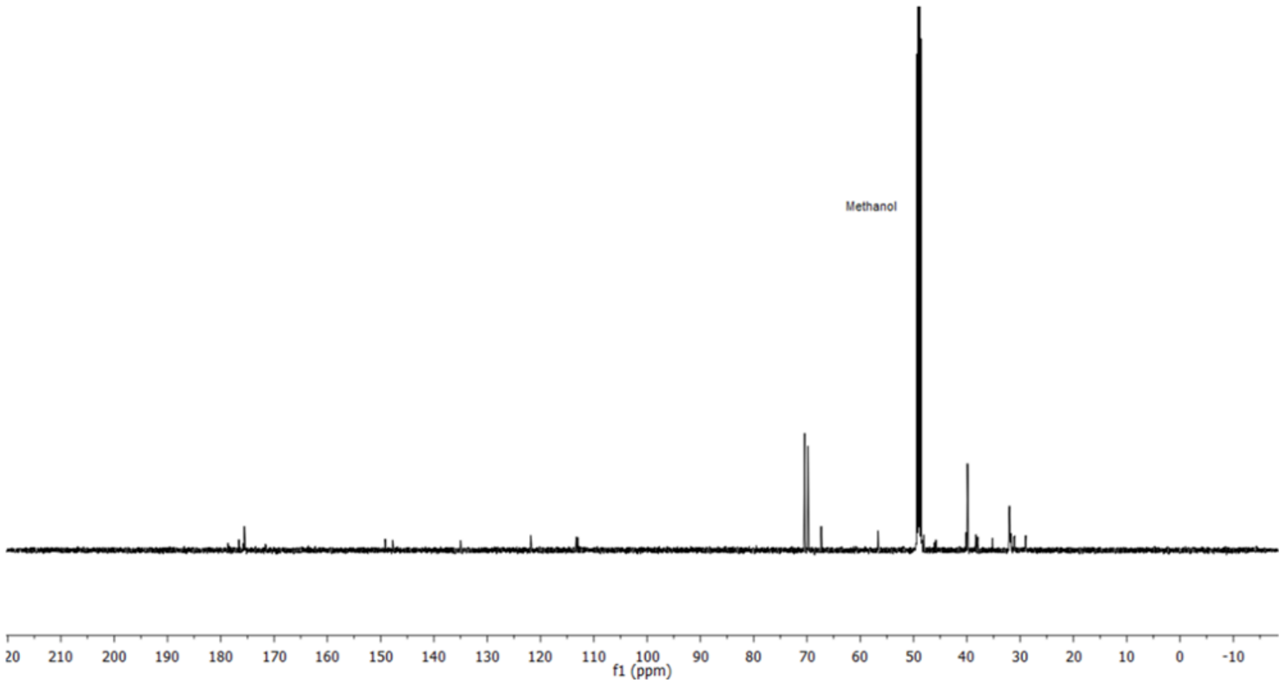

Figure S34. ^{13}C-NMR spectrum of compound **7** protected (126 MHz, D_2O).

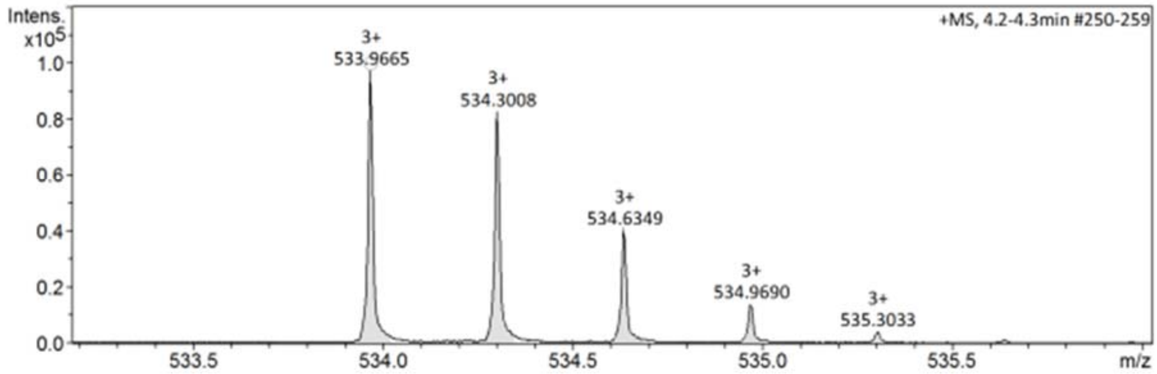

Figure S35. HR-ESI (ESI^+ Q-TOF) of compound **7** protected.

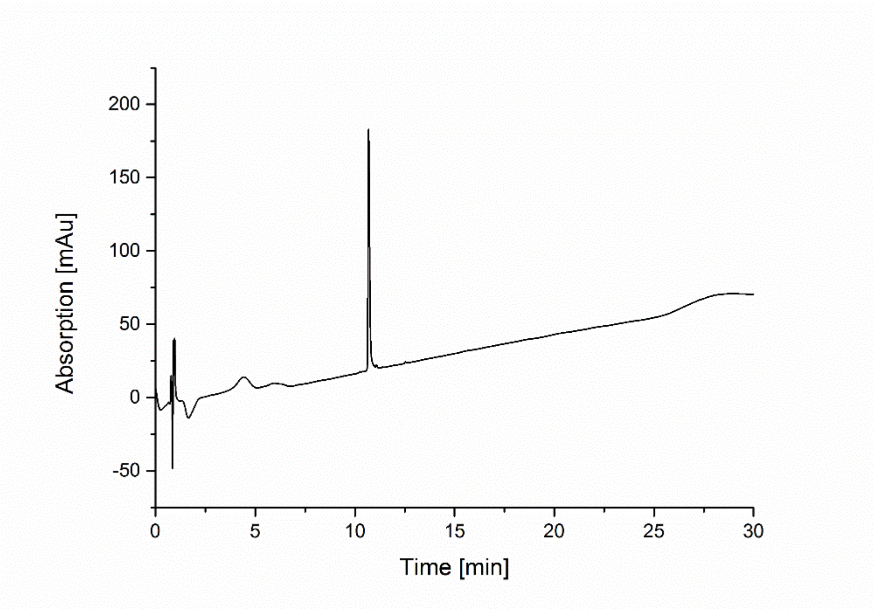

Figure S36. RP-HPLC chromatogram of compound **7** protected (gradient from 0% to 50% eluent B over 30 min at 25°C).

(**7**)

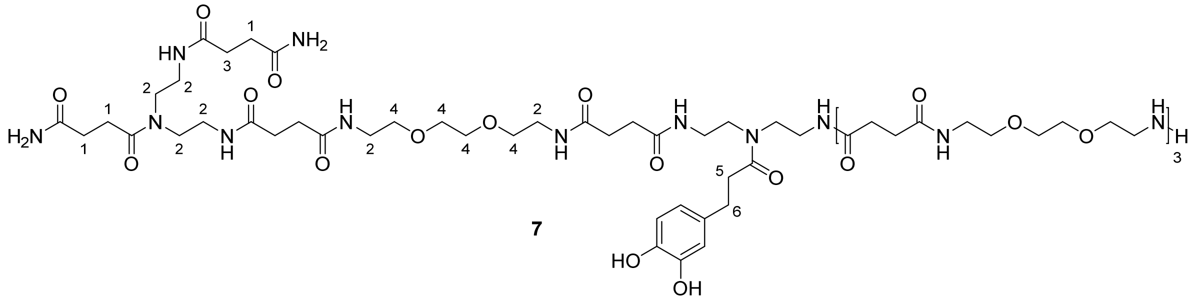

7

Compound **7** was obtained with a yield of 24% after deprotection, purification by preparative RP-HPLC and lyophilization.

^{1}H-NMR (500 MHz, D_2O) δ [ppm]: 8.45 (s, NH), 6.83 (d, J = 8.0 Hz, 1H, $H_{Aromatic}$), 6.77 (d, J = 2.1 Hz, 1H, $H_{Aromatic}$), 6.68 (dd, J = 8.0 Hz, 2.1 Hz, 1H, $H_{Aromatic}$), 3.78-3.56 (m, 32H, H-5), 3.51-3.20 (m, 32H, H-2), 2.79 (t, J = 7.0 Hz, 2H, H-4), 2.66 (m, 4H, H-3, H-6), 2.54-2.43 (m, 26H, H-1).

^{13}C-NMR (126 MHz, D_2O) δ [ppm]: 178.50, 175.91, 175.88, 175.81, 175.74, 175.66, 175.64, 171.96, 171.93, 144.91, 134.45, 117.15, 70.41, 69.81, 67.37, 39.88, 39.81, 38.25, 37.91, 35.23, 31.98, 31.94, 31.91, 31.85, 31.82, 31.76, 31.75, 31.70, 31.61, 31.60, 29.00.

HR-ESI-MS: calculated mass for $C_{69}H_{121}N_{16}O_{25}$ $[M+3H]^{3+}$ 524.6224, found 524.6221.

RP-HPLC (gradient from 0% to 50% eluent B over 30 min at 25°C):t_r=6.5 min, purity 85%.

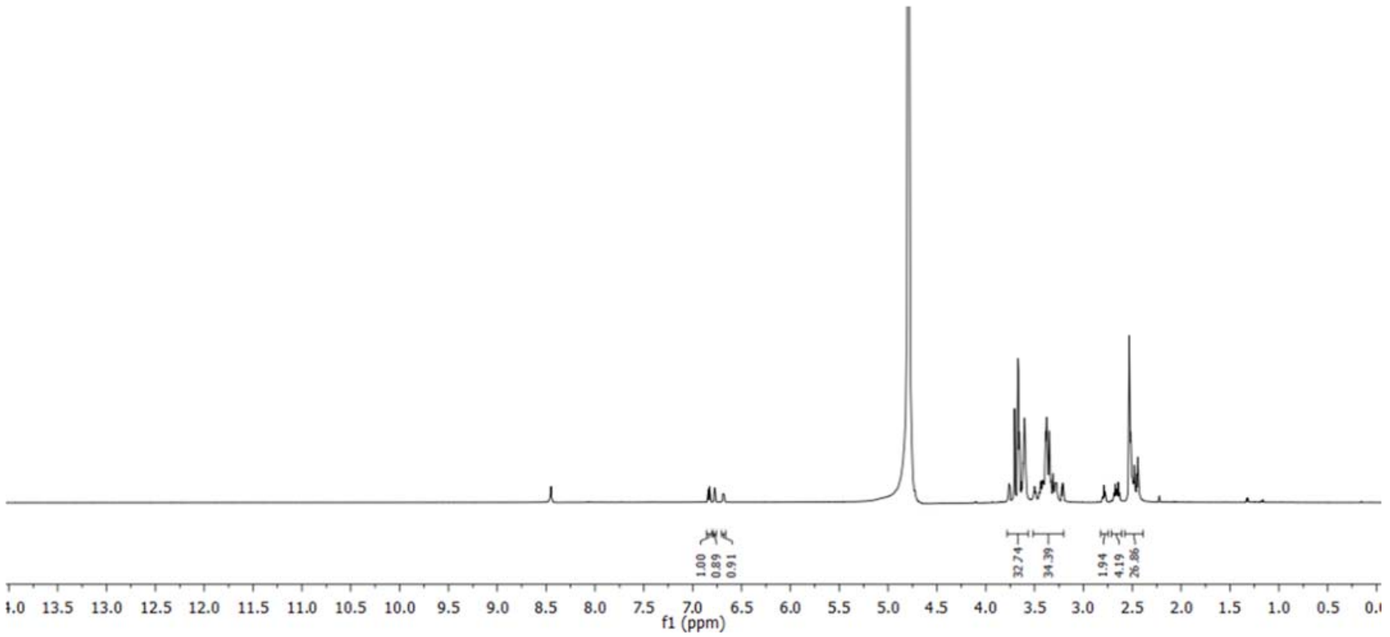

Figure S37. ^{1}H-NMR spectrum of compound **7** (600 MHz, D_2O).

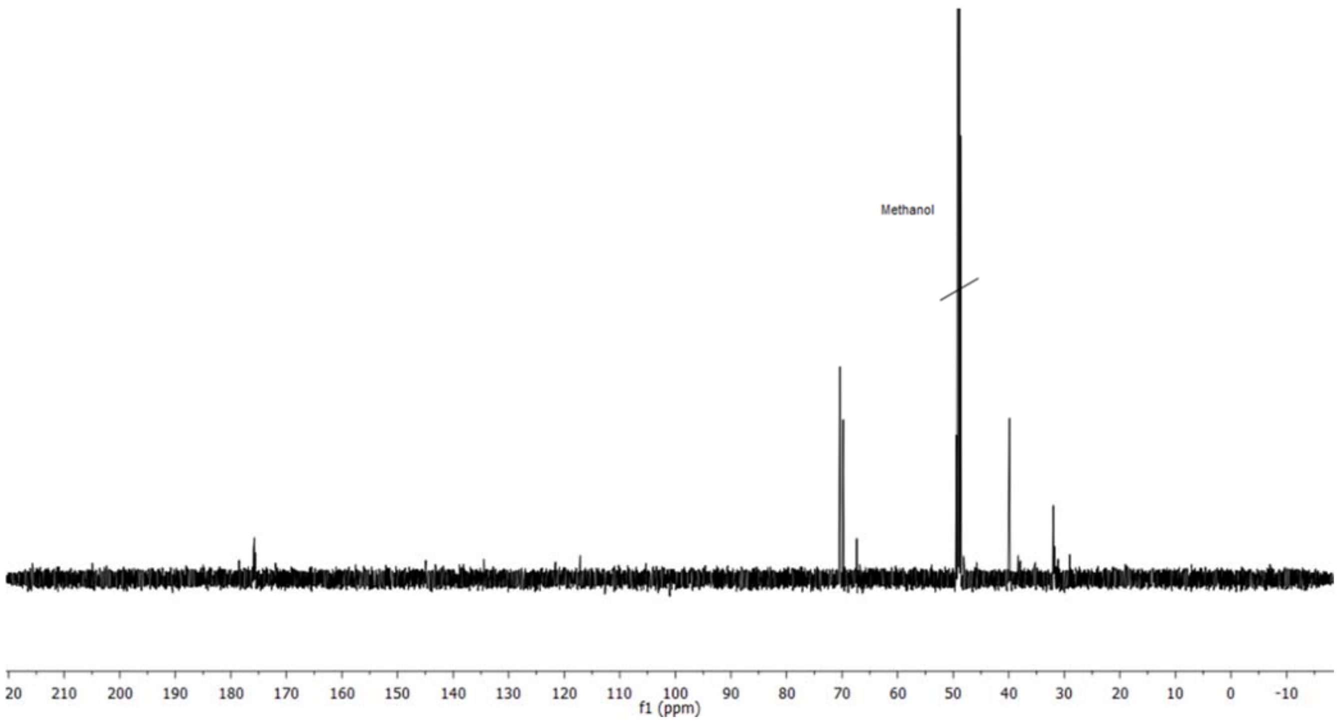

Figure S38. ^{13}C-NMR spectrum of compound **7** (126 MHz, D_2O).

Supporting Information

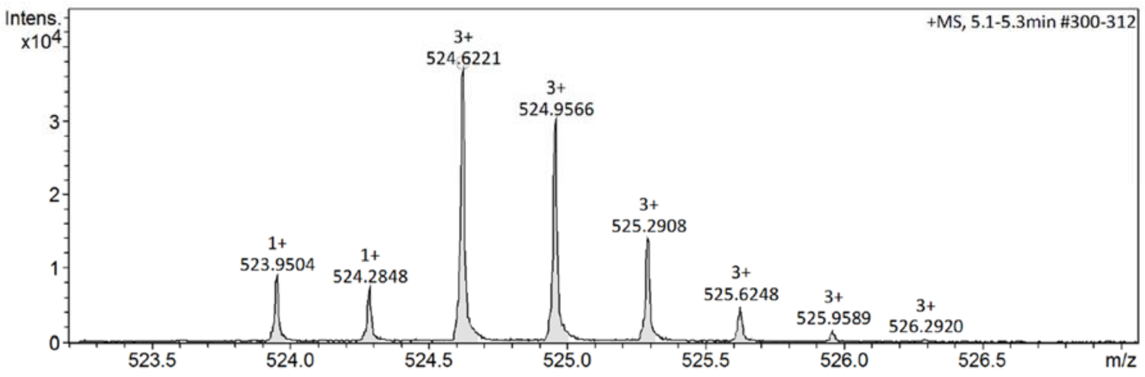

Figure S39. HR-ESI (ESI$^+$ Q-TOF) of compound **7**.

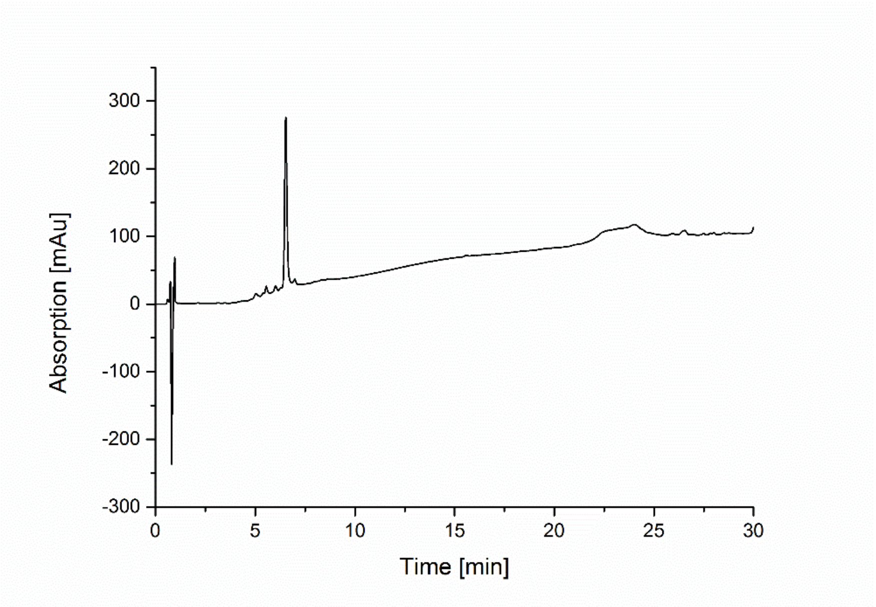

Figure S40. RP-HPLC chromatogram of compound **7** (gradient from 0% to 50% eluent B over 30 min at 25°C).

(**8**)

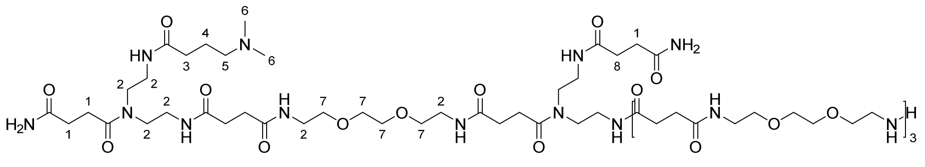

8

Compound **8** was obtained with a yield of 34% after purification by preparative RP-HPLC and lyophilization.

^{1}H-NMR (600 MHz, D_2O) δ [ppm]: 8.48 (s, NH), 3.75-3.56 (m, 32H, H-7), 3.52-3.17 (m, 32H, H-2, Methanol), 3.11 (m, 2H, H-5) 2.86 (m, 6H, H-6), 2.67 (m, 4H, H-3, H-8), 2.55-2.27 (m, 26H, H-1), 1.95 (m, 2H, H-4).

^{13}C-NMR (126 MHz, D_2O) δ [ppm]: 70.58, 70.47, 70.45, 69.89, 69.86, 69.85, 43.63, 40.11, 39.90, 31.99, 31.91, 31.88, 31.84, 31.82, 31.78, 31.63.

HR-ESI-MS: calculated mass for $C_{66}H_{124}N_{17}O_{23}$ $[M+3H]^{3+}$ 507.6347, found 507.6356.

RP-HPLC (gradient from 0% to 50% eluent B over 30 min at 25°C):t_r=1.2 min, purity 91%.

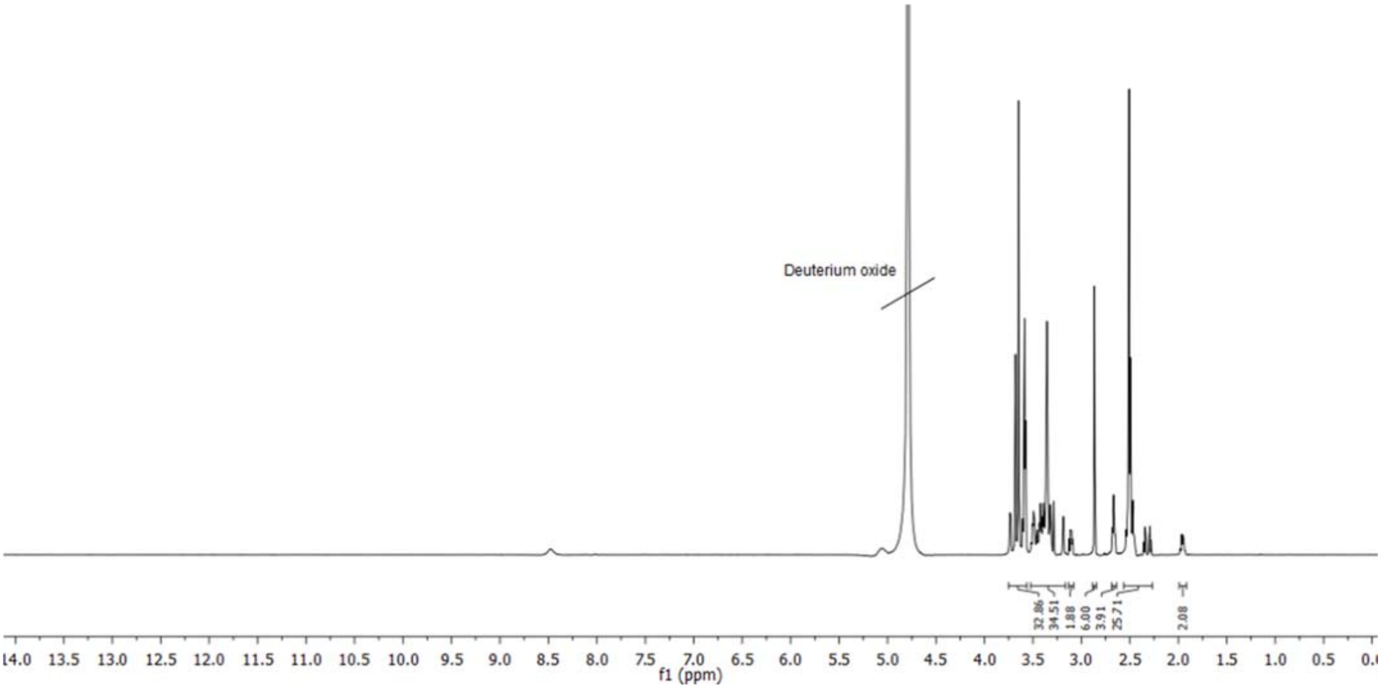

Figure S41. ^{1}H-NMR spectrum of compound **8** (500 MHz, D_2O).

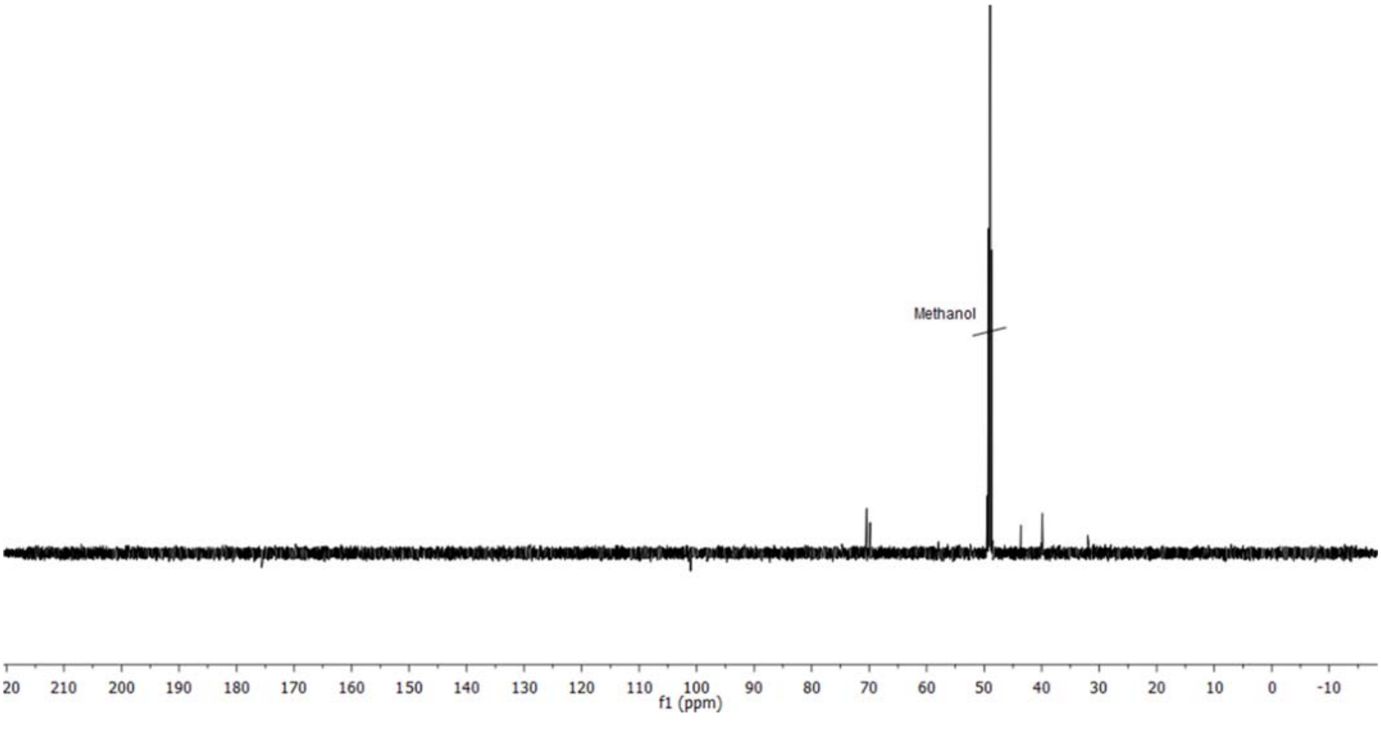

Figure S42. ^{13}C-NMR spectrum of compound **8** (126 MHz, D_2O).

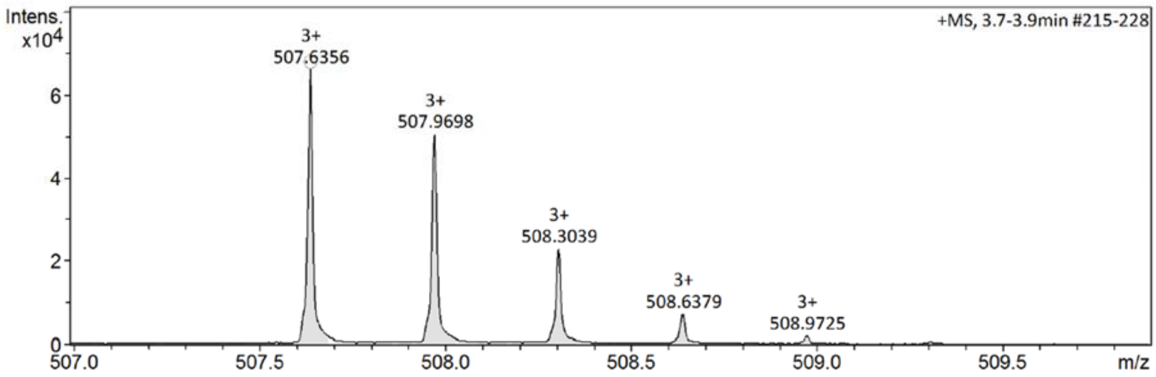

Figure S43. HR-ESI (ESI^+ Q-TOF) of compound **8**.

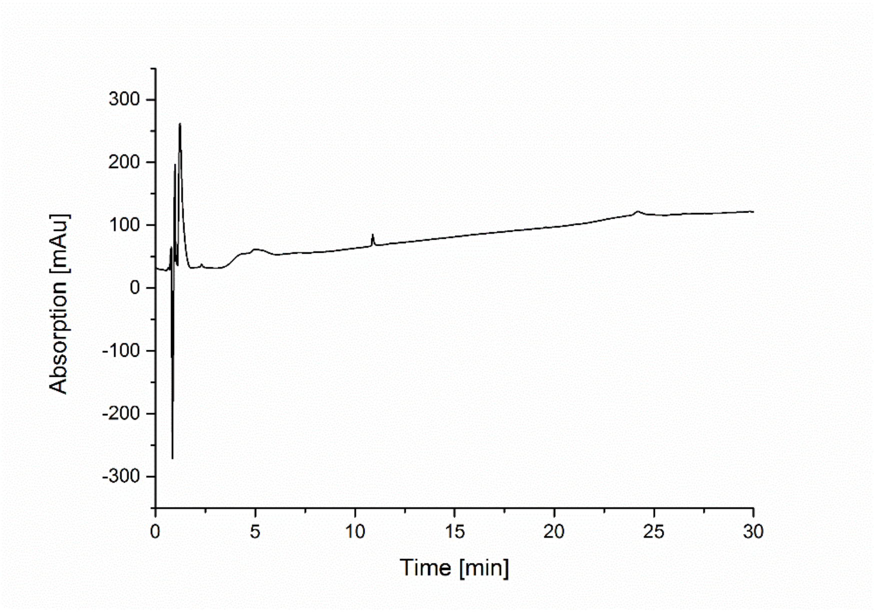

Figure S44. RP-HPLC chromatogram of compound **8** (gradient from 0% to 50% eluent B over 30 min at 25°C).

(**9**) *protected*

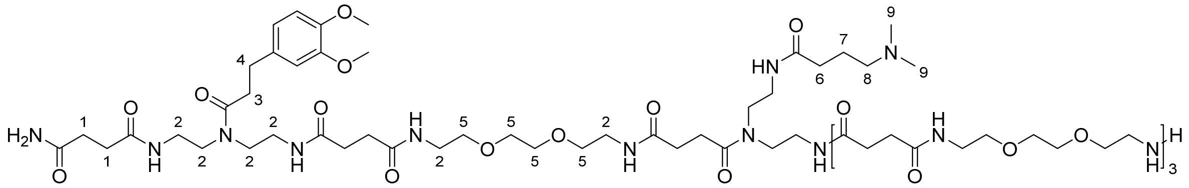

Compound **9** protected was obtained with a yield of 67% after cleavage from solid support and lyophilization.

^{1}H-NMR (500 MHz, D_2O) δ [ppm]: 8.62 (s, NH), 7.05 (d, J = 8.2 Hz, 1H, $H_{Aromatic}$), 7.02 (d, J = 1.9 Hz, 1H, $H_{Aromatic}$), 6.92 (dd, J = 8.2, 1.9 Hz, 1H, $H_{Aromatic}$), 3.91 (s, 3H, OCH_3), 3.89 (s, 3H, OCH_3), 3.85-3.64 (m, 32H, H-5), 3.60-3.26 (m, 32H, H-2), 3.19 (m, 2H, H-8), 2.95 (m, 8H, 4-H, 9-H), 2.75 (m, 4H, 6-H, 3-H), 2.62-2.35 (m, 24H, 1-H), 2.04 (m, 2H, 7-H).

^{13}C-NMR (126 MHz, D_2O) δ [ppm]: 177.88, 176.29, 175.39, 175.36, 175.28, 175.21, 175.16, 175.01, 171.29, 148.70, 147.28, 134.64, 121.50, 112.99, 112.71, 70.12, 69.98, 69.38, 66.91, 57.52, 56.39, 56.32, 47.66, 45.43, 43.24, 39.67, 39.43, 37.92, 37.60, 37.51, 34.68, 32.63, 32.52, 31.60, 31.47, 31.38, 31.20, 30.78, 30.65, 28.58, 20.60, 20.55.

HR-ESI-MS: calculated mass for $C_{73}H_{131}N_{16}O_{24}$ $[M+3H]^{3+}$ 538.6502, found 538.6503.

RP-HPLC (gradient from 0% to 50% eluent B over 30 min at 25°C): t_r=10.6 min, purity 89%.

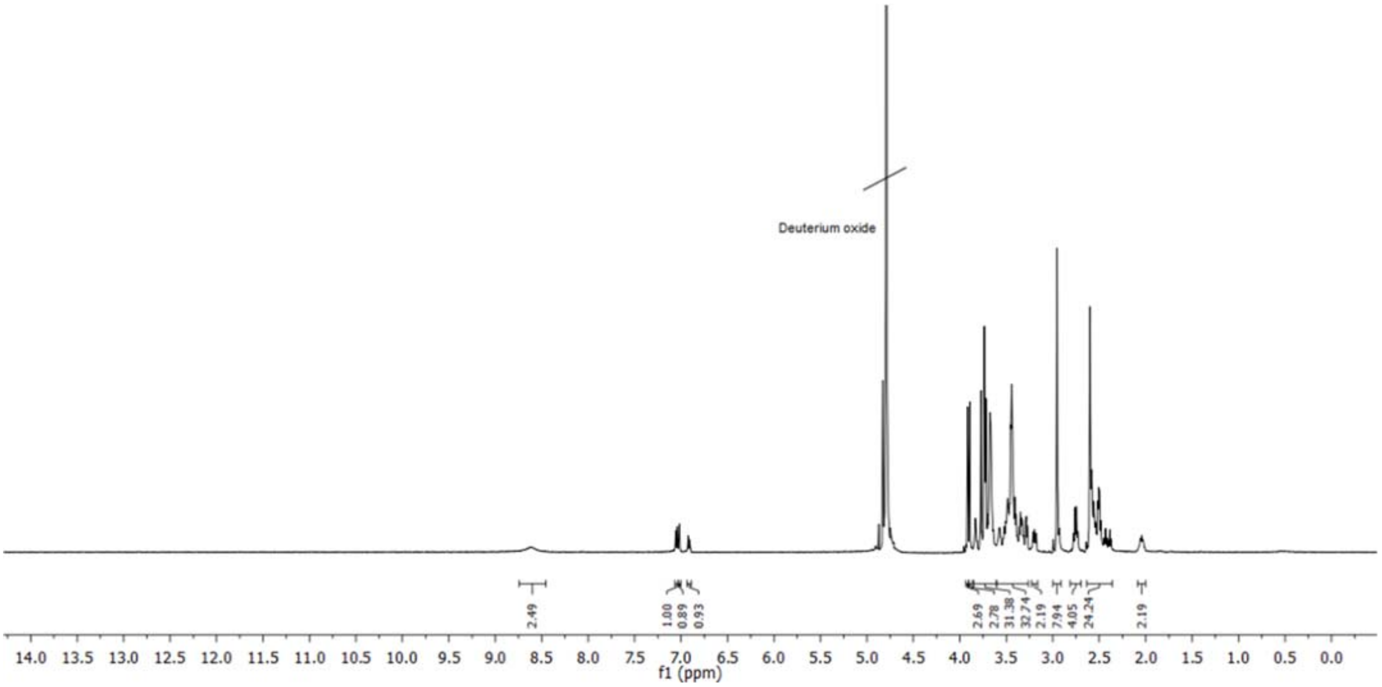

Figure S45. ^{1}H-NMR spectrum of compound **9** protected (500 MHz, D_2O).

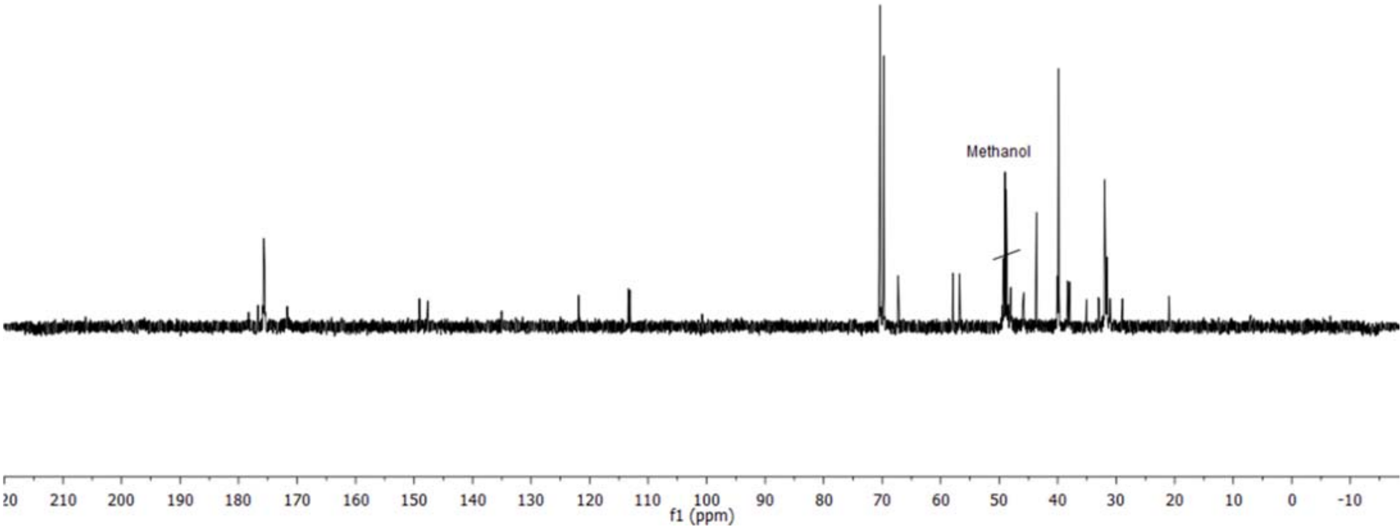

Figure S46. ^{13}C-NMR spectrum of compound **9** protected (126 MHz, D_2O).

Supporting Information

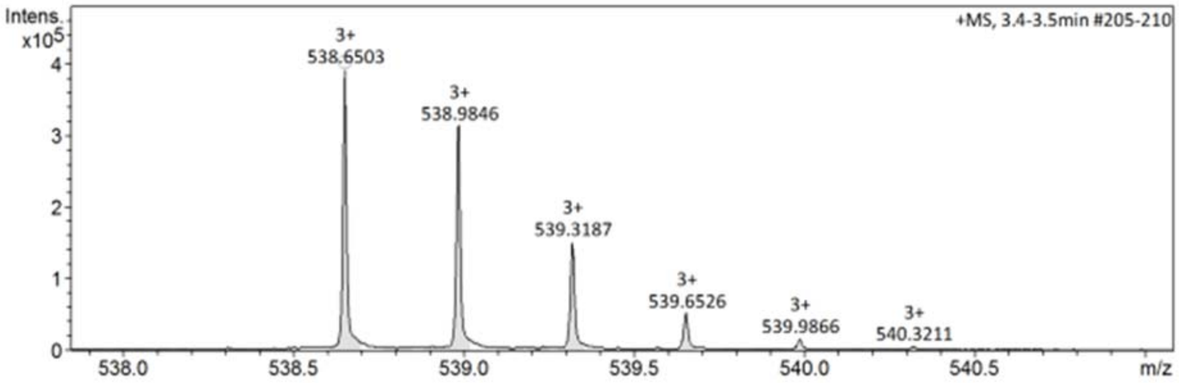

Figure S47. HR-ESI (ESI^+ Q-TOF) of compound **9** protected.

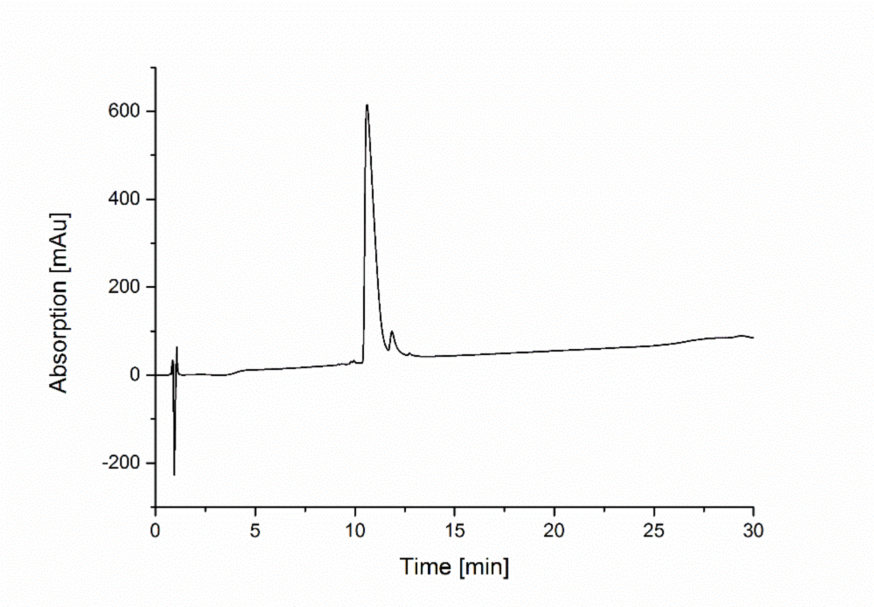

Figure S48. RP-HPLC chromatogram of compound **9** protected (gradient from 0% to 50% eluent B over 30 min at 25°C).

(**9**)

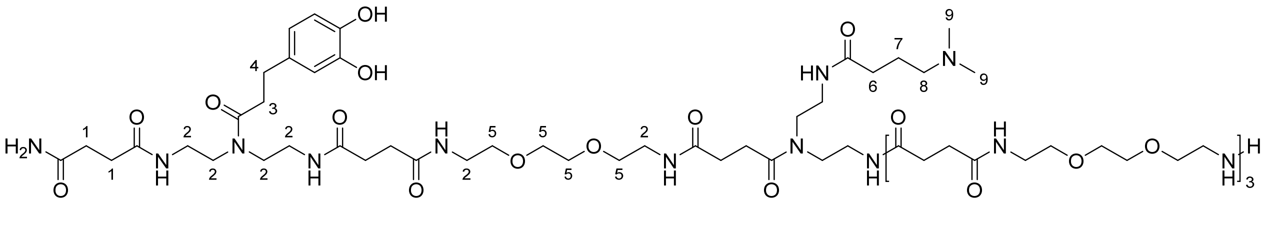

9

Compound **9** was obtained with a yield of 27% after deprotection, purification by preparative RP-HPLC and lyophilization.

^{1}H-NMR (600 MHz, D_2O) δ [ppm]: 8.44 (s, NH), 6.82 (d, J = 8.0 Hz, 1H, $H_{Aromatic}$), 6.76 (s, 1H, $H_{Aromatic}$), 6.67 (d, J = 8.0 HZ, 1H, $H_{Aromatic}$), 3.76-3.55 (m, 32H, H-5), 3.51-3.18 (m, 32H, H-2), 3.11 (t,

J = 8.1Hz, 2H, H-8), 2.87 (s, 6H, H-9), 2.77 (t, J = 7.2 Hz, 2H, H-4), 2.65 (m, 4H, H-3, H-6), 2.55-2.28 (m, 24H, H-1), 1.97 (m, 2H, H-7).

^{13}C-NMR (126 MHz, D_2O) δ [ppm]: 175.76, 175.71, 175.64, 175.54, 171.78, 121.52, 117.10, 70.53, 70.39, 69.79, 67.32, 57.93, 46.02, 45.84, 40.08, 39.86, 39.80, 38.02, 37.94, 37.91, 35.20, 33.04, 32.92, 31.99, 31.95, 31.92, 31.86, 31.78, 31.63, 31.40, 31.08, 29.02, 28.97.

HR-ESI-MS: calculated mass for $C_{71}H_{127}N_{16}O_{24}$ $[M+3H]^{3+}$ 529.3064, found 529.3056.

RP-HPLC (gradient from 0% to 50% eluent B over 30 min at 25°C): t_r=6.3 min, purity 97%.

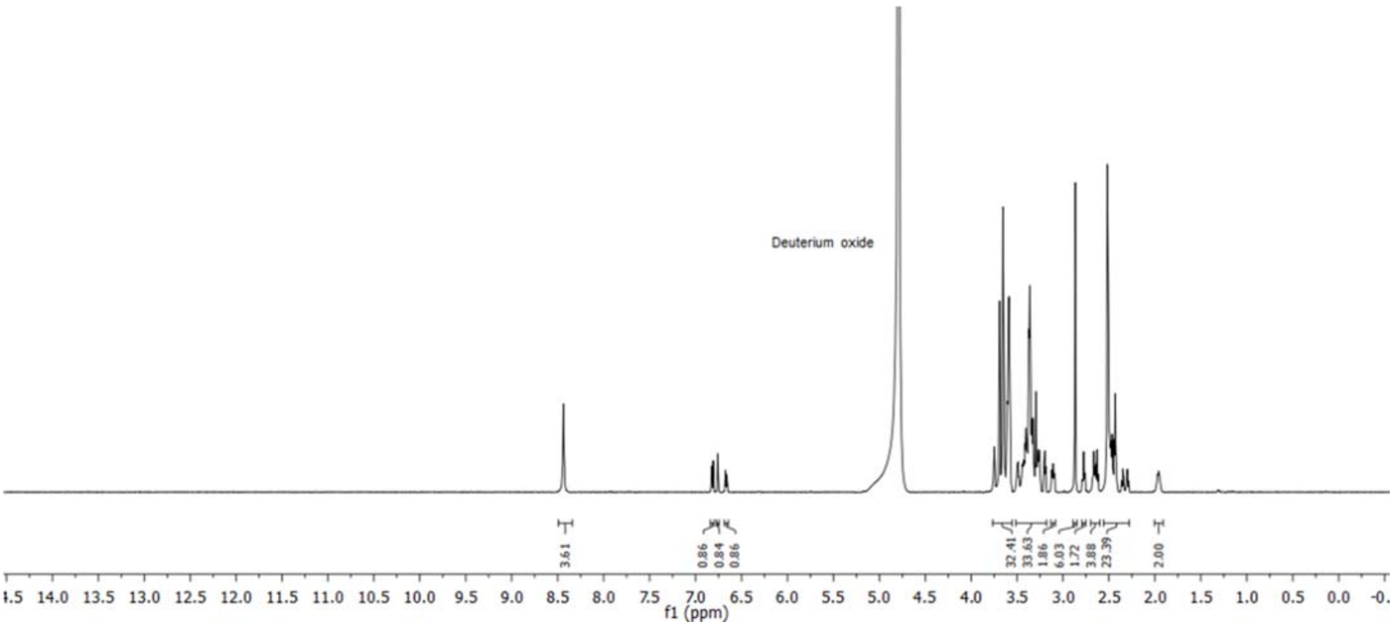

Figure S49. ^{1}H-NMR spectrum of compound **9** (500 MHz, D_2O).

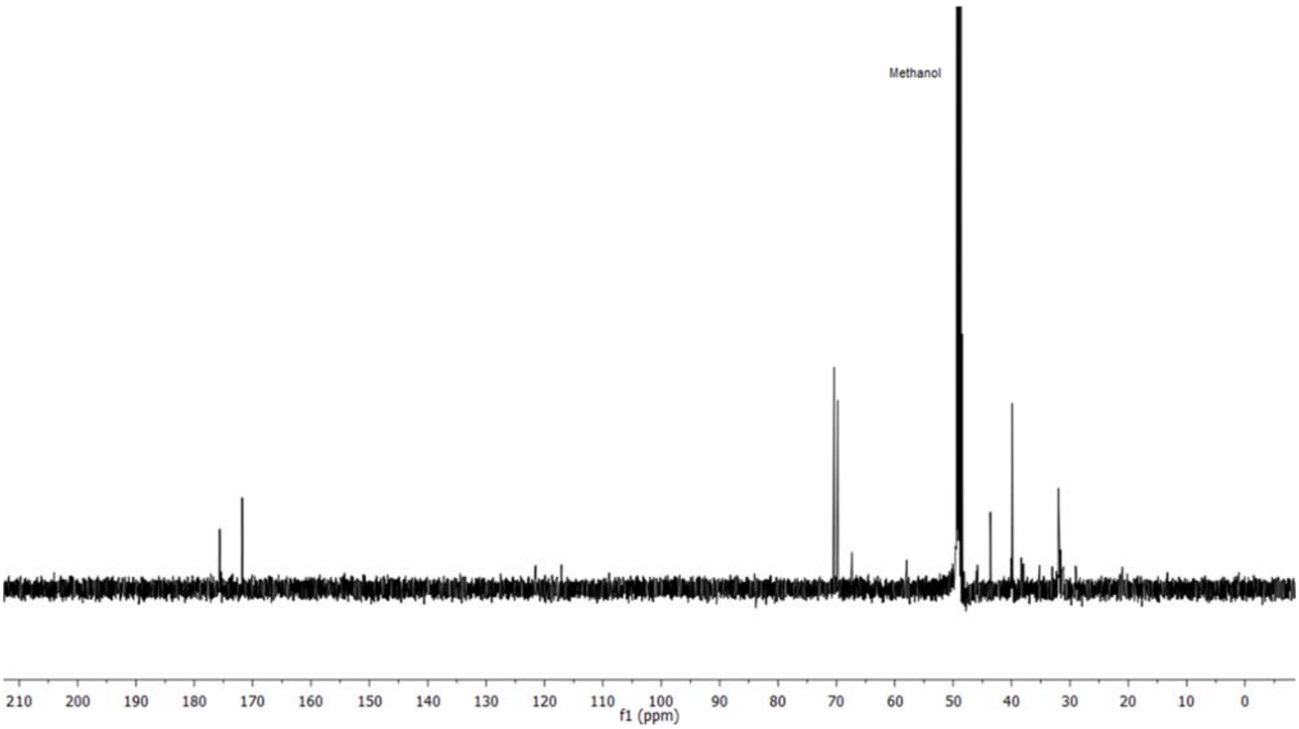

Figure S50. ^{13}C-NMR spectrum of compound **9** (126 MHz, D_2O).

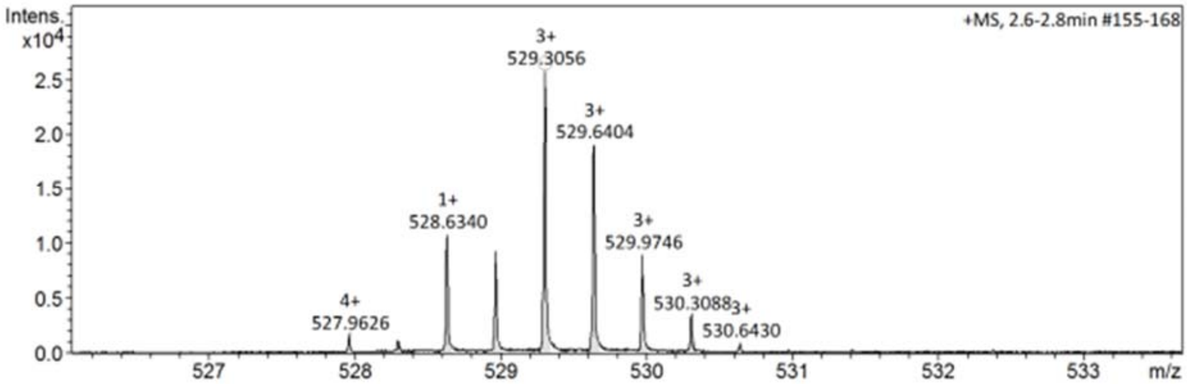

Figure S51. HR-ESI (ESI^+ Q-TOF) of compound **9**.

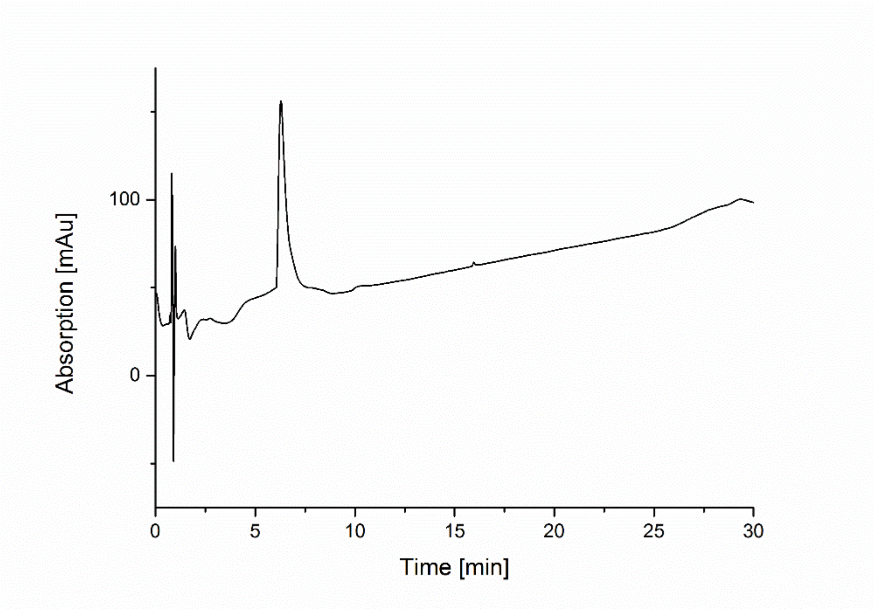

Figure S52. RP-HPLC chromatogram of compound **9** (gradient from 0% to 50% eluent B over 30 min at 25°C).

(**10**) *protected*

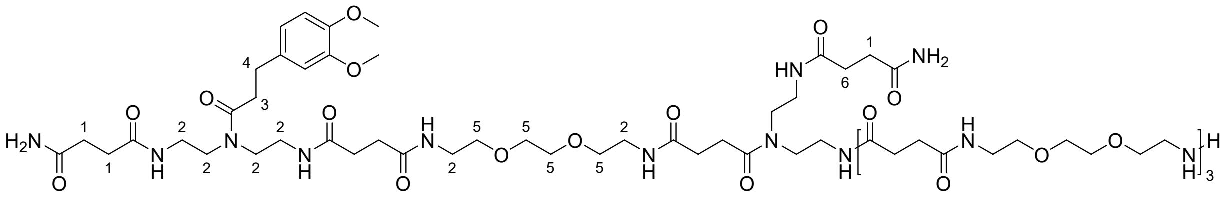

10

Compound **10** protected was obtained with a yield of 61% after cleavage from solid support and lyophilization.

^{1}H-NMR (500 MHz, D_2O) δ [ppm]: 8.45 (s, NH), 7.04 (d, J = 8.2 Hz, 1H, $H_{Aromatic}$), 7.01 (m, 1H, $H_{Aromatic}$), 6.91 (m, 1H, $H_{Aromatic}$), 3.91 (s, 3H, OCH_3), 3.89 (s, 3H, OCH_3), 3.84-3.62 (m, 32H, H-5), 3.58-3.25 (m, 32H, H-2), 2.94 (t, J = 7.2 Hz, 2H, H-4), 2.74 (m, 4H, H-3, H-6), 2.64-2.43 (m, 26H, H-1).

^{13}C-NMR (126 MHz, D_2O) δ [ppm]: 178.00, 177.88, 176.30, 175.43, 175.36, 175.29, 175.25, 175.21, 175.17, 175.03, 148.69, 147.27, 134.63, 121.49, 112.97, 112.69, 70.12, 69.97, 69.40, 69.37, 66.91, 56.38, 56.31, 47.73, 45.75, 45.43, 39.67, 39.46, 39.39, 37.92, 37.59, 37.52, 34.73, 34.68, 31.60, 31.53, 31.50, 31.47, 31.43, 31.39, 31.34, 31.30, 31.24, 31.19, 30.82, 30.78, 30.73, 30.65, 28.60.

HR-ESI-MS: calculated mass for $C_{71}H_{125}N_{16}O_{25}$ $[M+3H]^{3+}$ 533.9662, found 533.9661.

RP-HPLC (gradient from 0% to 50% eluent B over 30 min at 25°C): t_r=11.8 min, purity 98%.

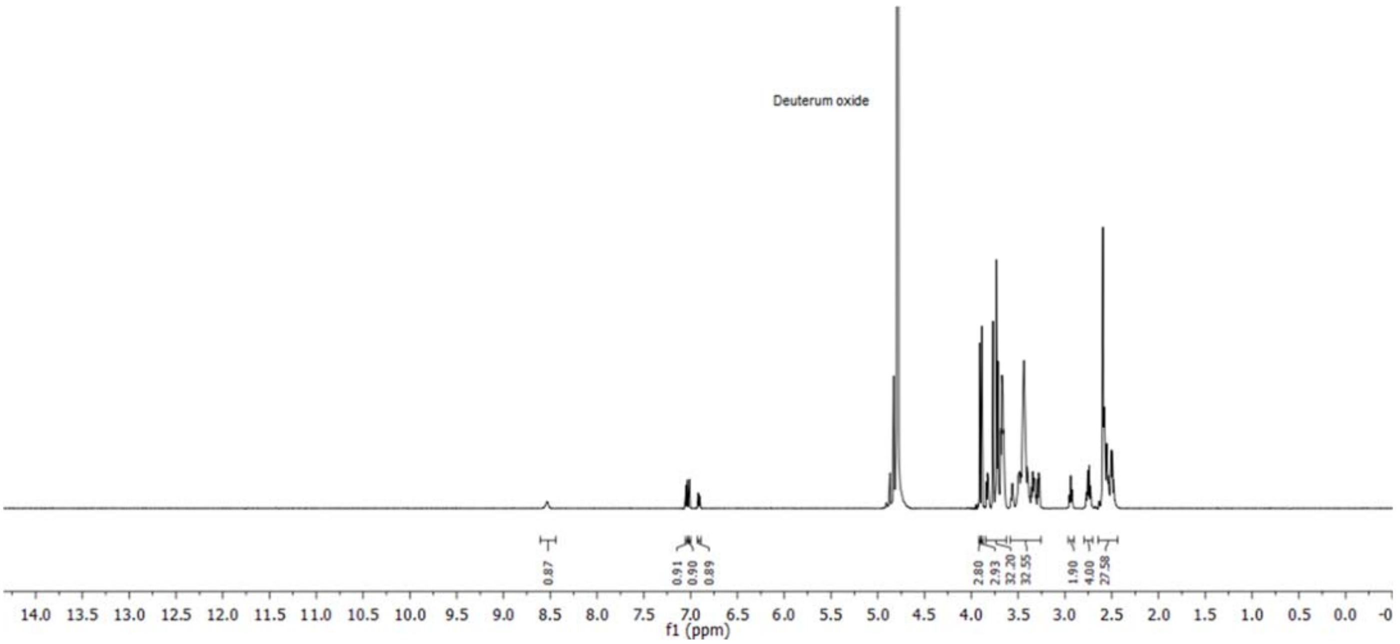

Figure S53. ^{1}H-NMR spectrum of compound **10** protected (500 MHz, D_2O).

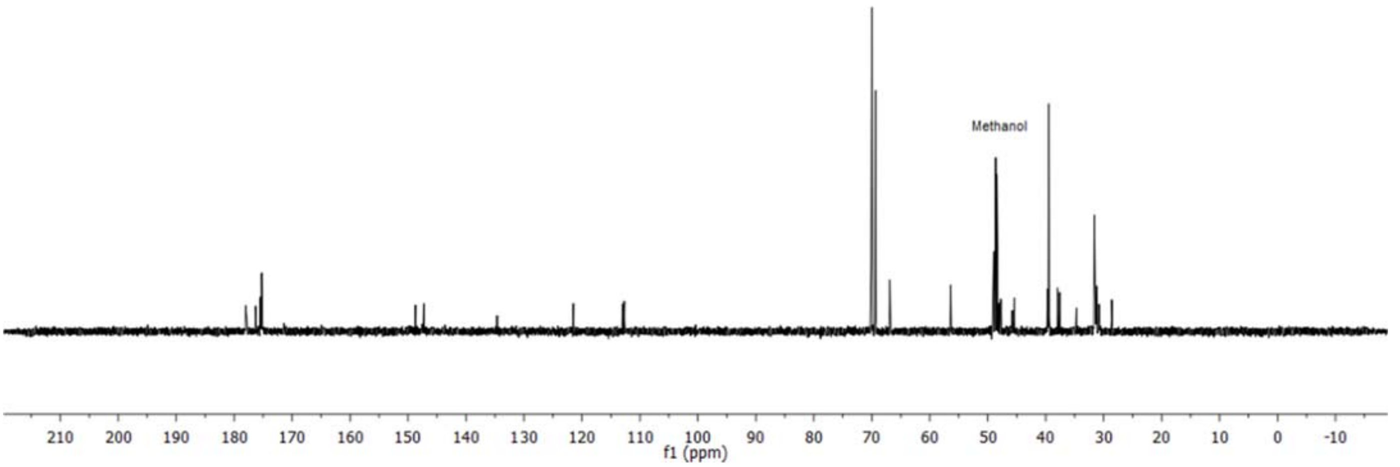

Figure S54. ^{13}C-NMR spectrum of compound **10** protected (126 MHz, D_2O).

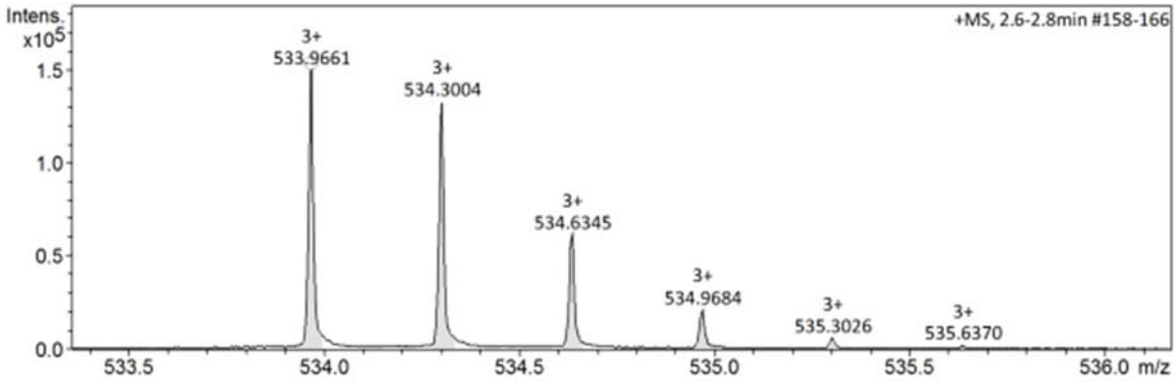

Figure S55. HR-ESI (ESI^+ Q-TOF) of compound **10** protected.

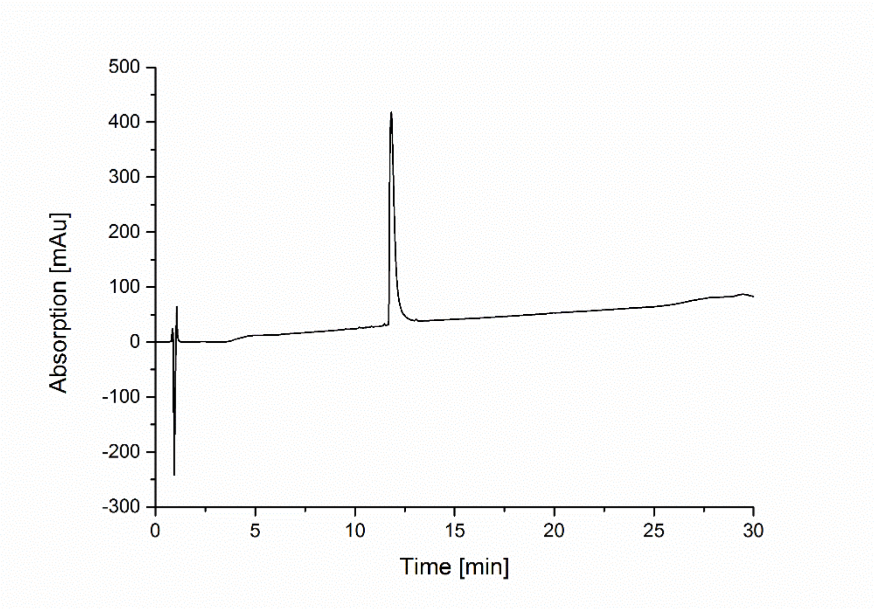

Figure S56. RP-HPLC chromatogram of compound **10** protected (gradient from 0% to 50% eluent B over 30 min at 25°C).

(**10**)

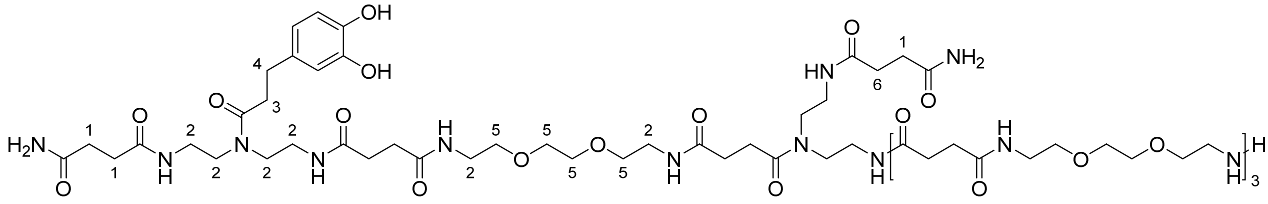

10

Compound **10** was obtained with a yield of 19% after deprotection, purification by preparative RP-HPLC and lyophilization.

^{1}H-NMR (500 MHz, D_2O) δ [ppm]: 8.5 (s, NH), 6.88 (d, J = 8.1 Hz, 1H, $H_{Aromatic}$), 7.82 (d, J = 2.0 Hz, 1H, $H_{Aromatic}$), 6.73 (d, J = 8.1 Hz, 2.0 Hz, 1H, $H_{Aromatic}$), 3.82-3.59 (m, 32H, H-5), 3.57-3.25 (m, 32H, H-2), 2.83 (t, J = 7.1 Hz, 2H, H-4), 2.70 (m, 4H, H-3, H-6), 2.62-2.45 (m, 26H, H-1).

^{13}C-NMR (126 MHz, D_2O) δ [ppm]: 178.01, 177.97, 176.35, 176.33, 175.41, 175.36, 175.32, 175.24, 175.21, 175.15, 144.46, 142.84, 134.05, 121.12, 116.73, 116.68, 70.10, 69.96, 69.38, 69.35, 66.88, 47.70, 45.39, 39.64, 39.43, 39.37, 37.91, 37.88, 37.58, 37.50, 34.76, 34.75, 31.61, 31.57, 31.53, 31.50, 31.45, 31.38, 31.28, 31.22, 30.93, 30.80, 30.75, 30.71, 30.66, 28.58.

HR-ESI-MS: calculated mass for $C_{69}H_{121}N_{16}O_{25}$ $[M+3H]^{3+}$ 524.6224, found 524.6222.

RP-HPLC (gradient from 0% to 50% eluent B over 30 min at 25°C): t_r=9.8 min, purity 89%.

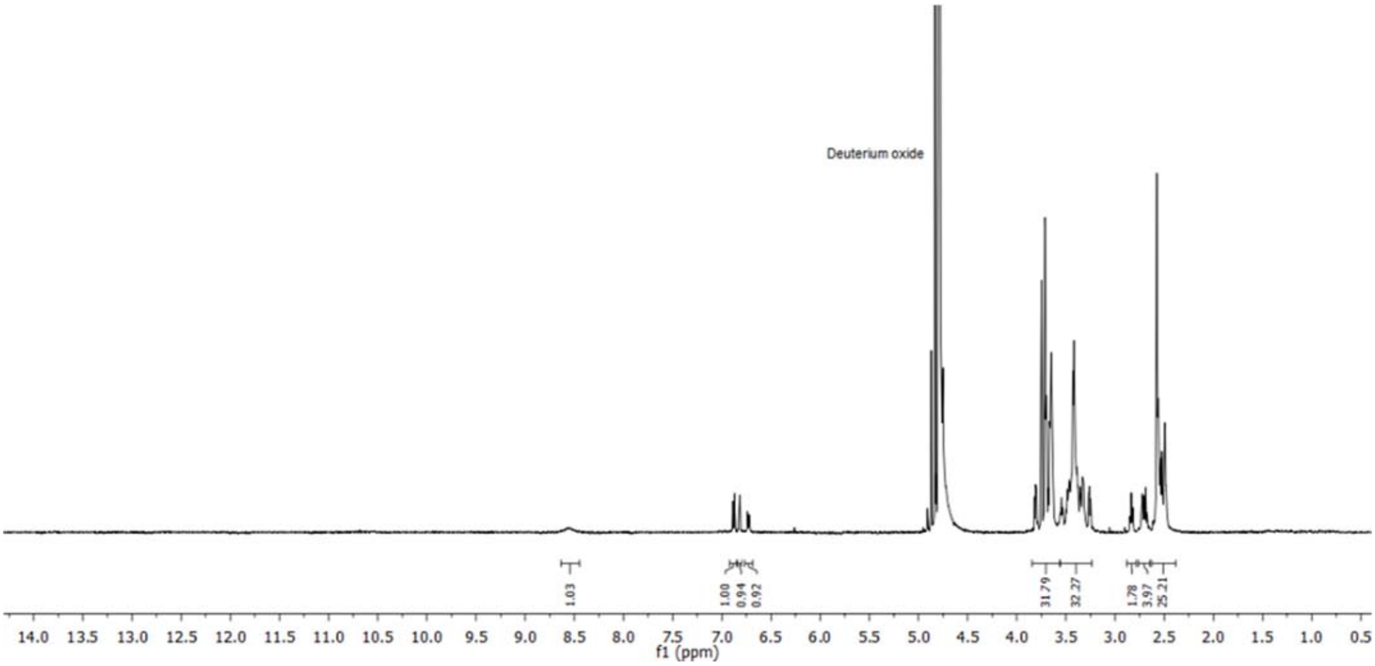

Figure S57. ^{1}H-NMR spectrum of compound **10** (500 MHz, D_2O).

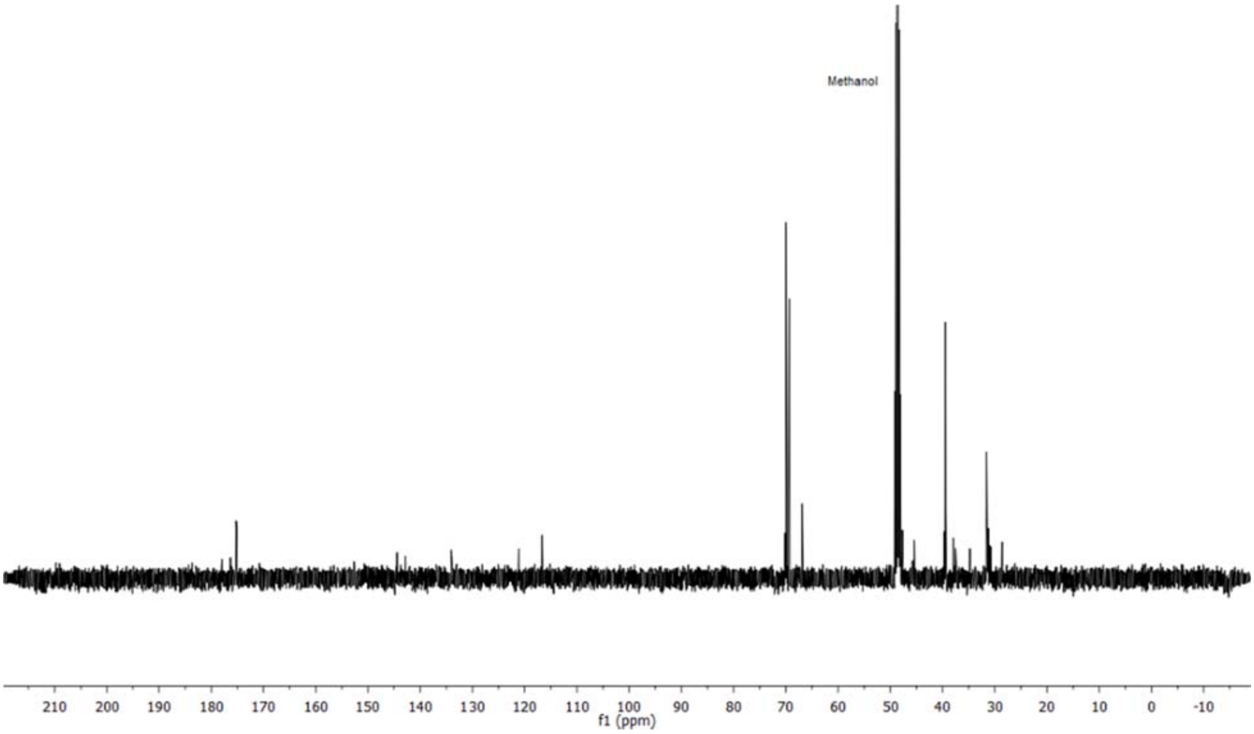

Figure S58. ^{13}C-NMR spectrum of compound **10** (126 MHz, D_2O).

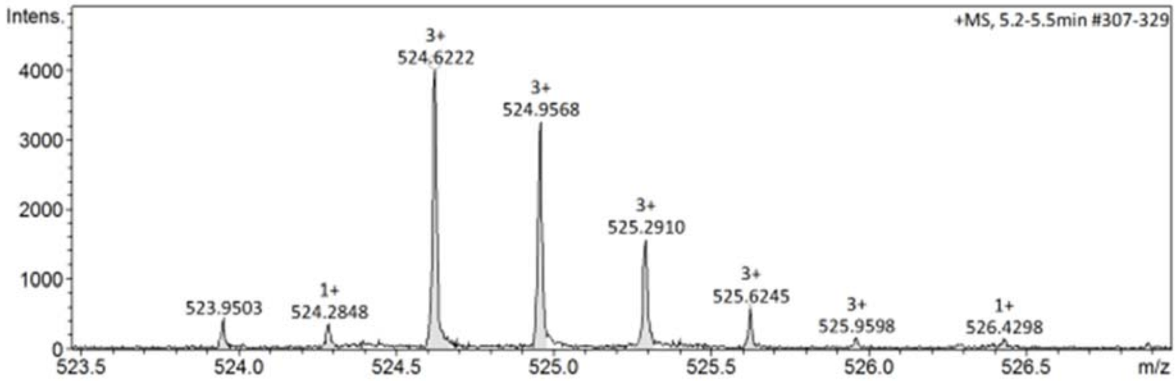

Figure S59. Figure 59: HR-ESI (ESI$^+$ Q-TOF) of compound **10**.

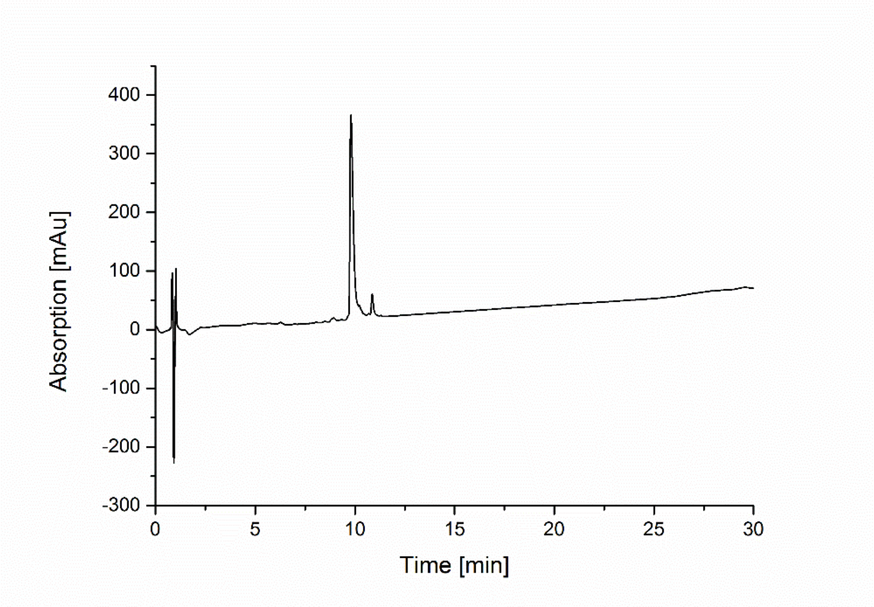

Figure S60. RP-HPLC chromatogram of compound **10** (gradient from 0% to 50% eluent B over 30 min at 25°C).

(**11**) *protected*

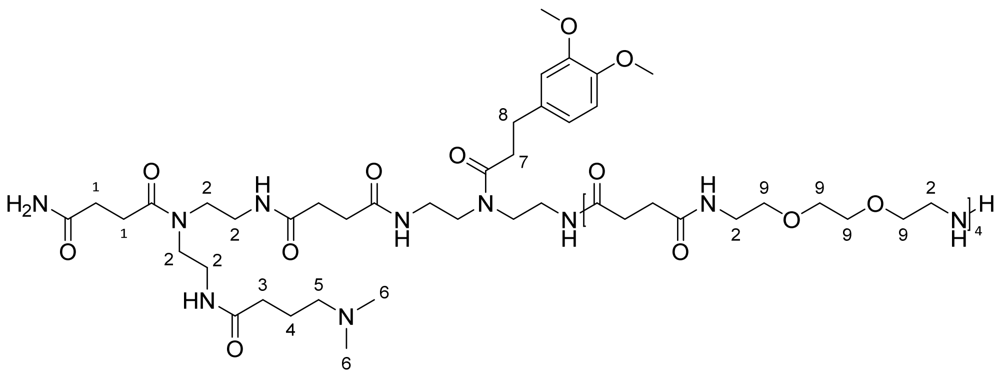

Compound **11** protected was obtained with a yield of 72% after cleavage from solid support and lyophilization

Supporting Information

^{1}H-NMR (500 MHz, D_2O) δ [ppm]: 8.50 (s, NH), 7.04 (d, J = 8.2 Hz, 1H, $H_{Aromatic}$), 7.01 (d, J = 2.0 Hz, 1H, $H_{Aromatic}$), 6.91 (dd, J = 8.2, 2.0 Hz, 1H, $H_{Aromatic}$), 3.91 (s, 3H, OCH_3), 3.89 (s, 3H, OCH_3), 3.75-3.52 (m, 32H, H-9), 3.49-3.18 (m, 32H, H-2), 3.10 (m, 2H, H-5), 2.86 (m, 8H, 6-H, 8-H), 2.65 (m, 4H, 3-H, 7-H), 2.55-2.27 (m, 24H, 1-H), 1.94 (m, 2H, 4-H).

^{13}C-NMR (126 MHz, D_2O) δ [ppm]: 178.62, 176.66, 175.74, 175.68, 175.66, 175.63, 175.59, 175.56, 175.5, 175.39, 171.46, 149.09, 147.67, 135.01, 121.83, 113.33, 113.07, 70.51, 70.36, 69.75, 67.29, 57.91, 56.77, 56.69, 43.62, 40.05, 39.81, 38.19, 37.95, 37.87, 35.14, 33.01, 32.88, 31.99, 31.95, 31.92, 31.84, 31.77, 31.72, 31.56, 30.93, 28.92, 28.82, 20.94.

HR-ESI-MS: calculated mass for $C_{73}H_{131}N_{16}O_{24}$ $[M+3H]^{3+}$ 538.6502, found 538.6497.

RP-HPLC (gradient from 0% to 50% eluent B over 30 min at 25°C):t_r=11.1 min, purity 92%.

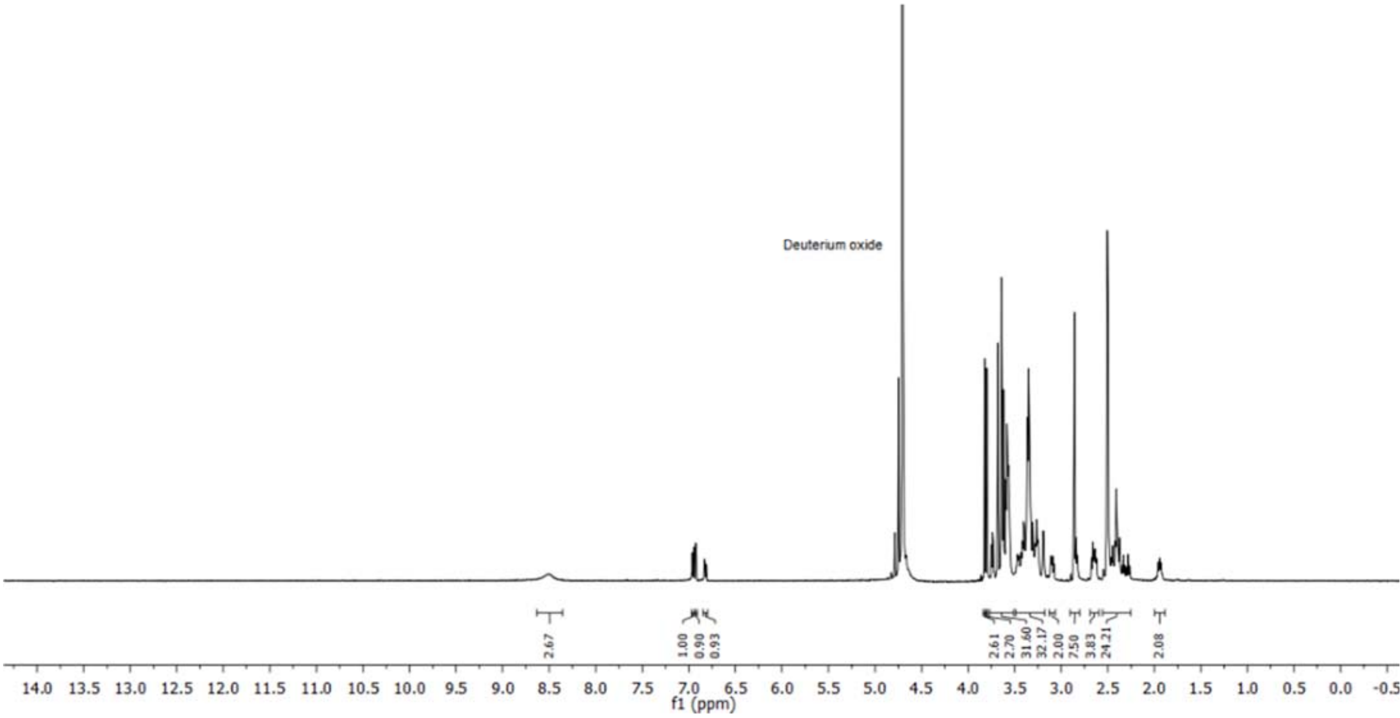

Figure S61. ^{1}H-NMR spectrum of compound **11** protected (500 MHz, D_2O).

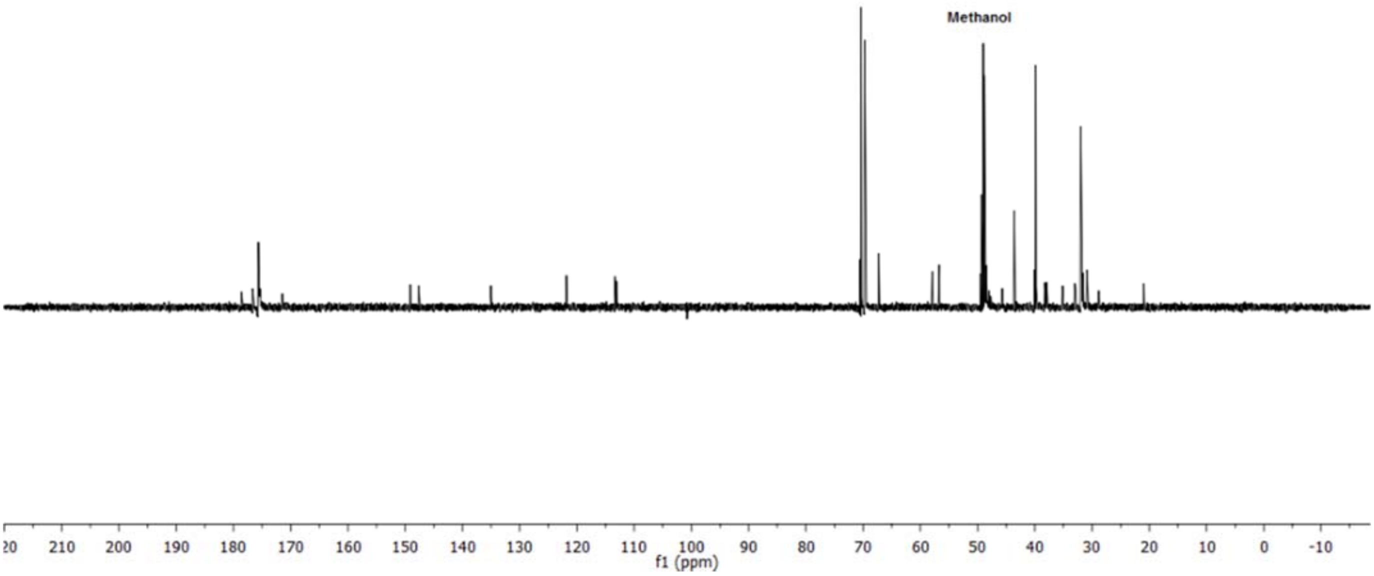

Figure S62. ^{13}C-NMR spectrum of compound **11** protected (126 MHz, D_2O).

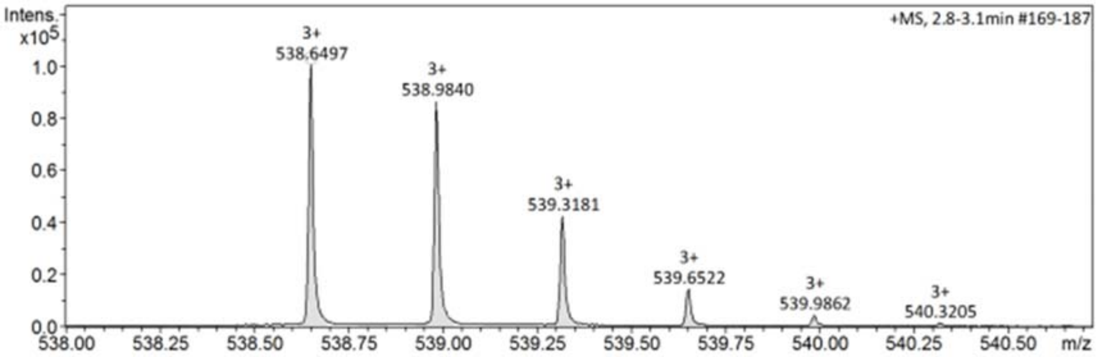

Figure S63. HR-ESI (ESI^+ Q-TOF) of compound **11** protected.

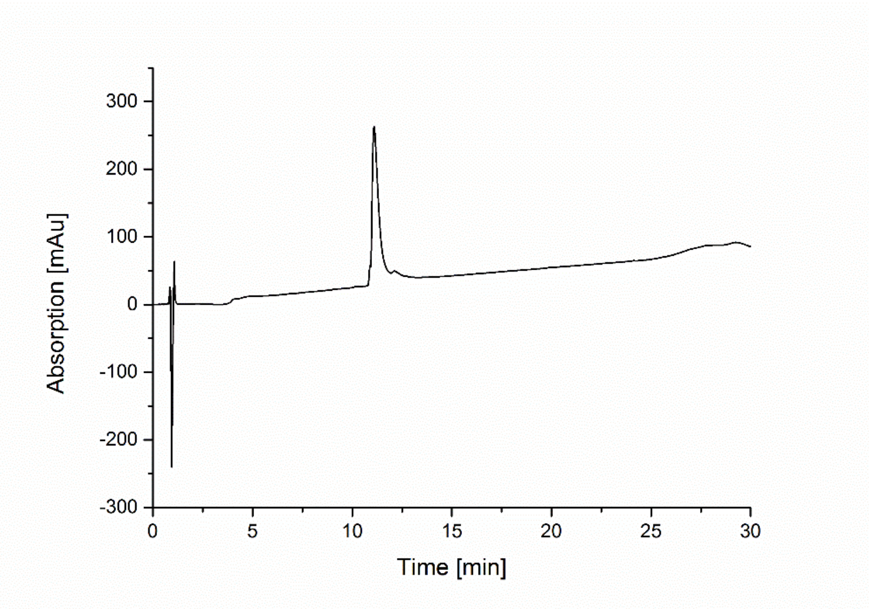

Figure S64. RP-HPLC chromatogram of compound **11** protected (gradient from 0% to 50% eluent B over 30 min at 25°C).

(**11**)

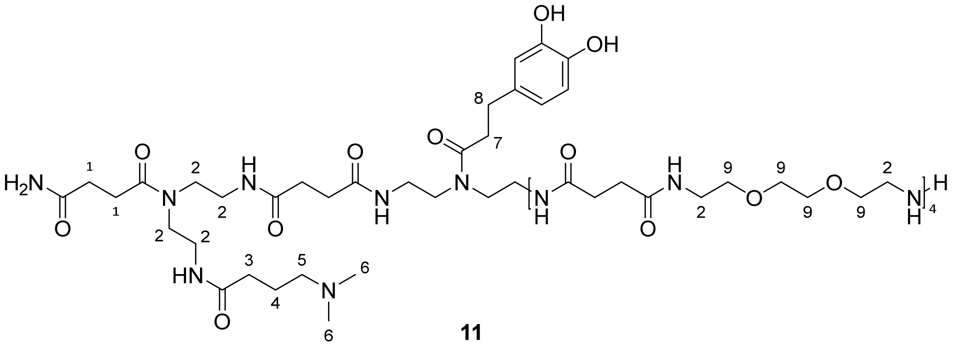

11

Compound **11** was obtained with a yield of 28% after deprotection, purification by preparative RP-HPLC and lyophilization.

^{1}H-NMR (500 MHz, D_2O) δ [ppm]: 8.43 (s, NH), 6.85 (m, 1H, $H_{Aromatic}$), 6.79 (m, 1H, $H_{Aromatic}$), 6.70 (m, 1H, $H_{Aromatic}$), 3.82-3.60 (m, 32H, H-9), 3.59-3.20 (m, 32H, H-2), 3.15 (m, 2H, H-5), 2.91 (s, 6H, H-6), 2.80 (m, 2H, H-8), 2.69 (m, 4H, H-3, H-7), 2.64-2.30 (m, 24H, H-1), 2.01 (m, 2H, 4-H).

^{13}C-NMR (126 MHz, D_2O) δ [ppm]: 178.60, 176.69, 175.73, 175.68, 175.60, 175.54, 175.49, 175.43, 175.39, 175.38, 175.36, 171.57, 144.94, 143.32, 134.42, 121.48, 117.11, 117.06, 70.53, 70.39, 69.78, 67.30, 57.93, 43.62, 40.09, 39.86, 39.79, 38.26, 37.99, 37.90, 35.24, 33.01, 32.89, 31.99, 31.95, 31.91, 31.85, 31.80, 31.75, 31.70, 31.38, 30.94, 28.93, 28.83.

HR-ESI-MS: calculated mass for $C_{71}H_{127}N_{16}O_{24}$ $[M+3H]^{3+}$ 529.3064, found 529.3056.

RP-HPLC (gradient from 0% to 50% eluent B over 30 min at 25°C): t_r=9.5 min, purity 90%.

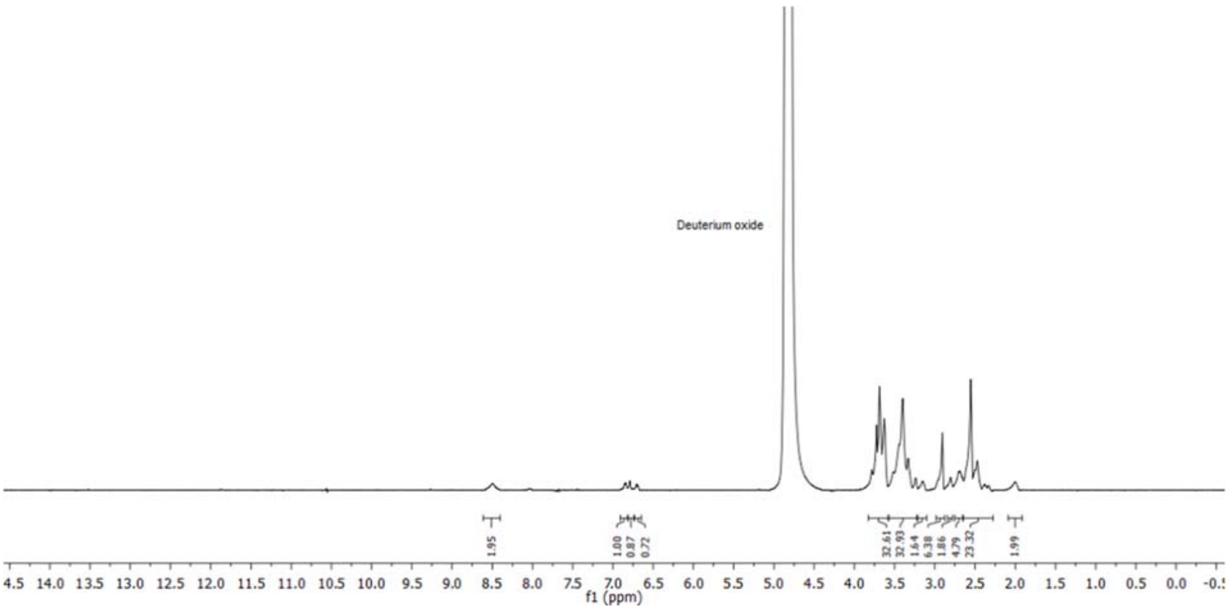

Figure S65. ^{1}H-NMR spectrum of compound **11** (500 MHz, D_2O).

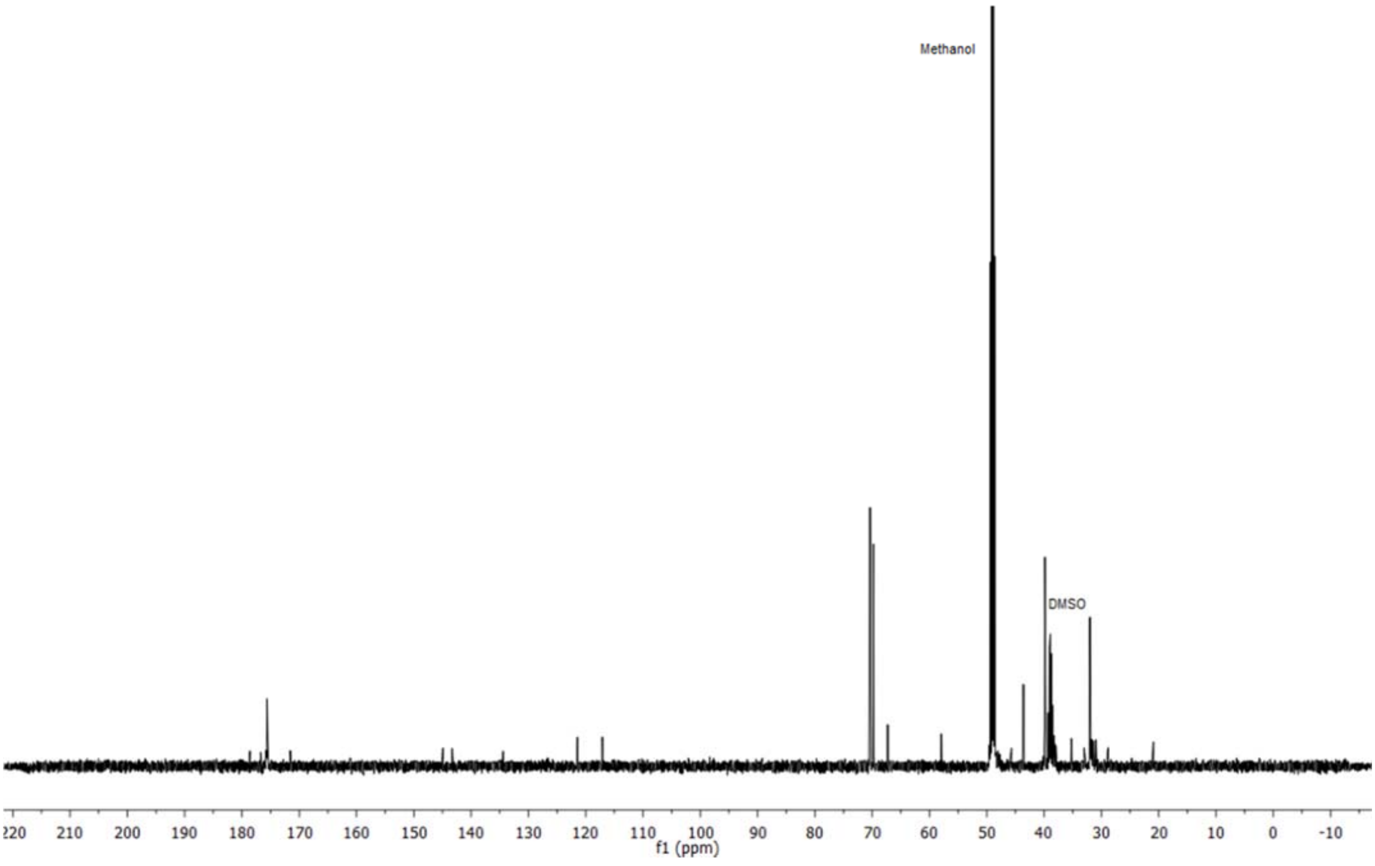

Figure S66. ^{13}C-NMR spectrum of compound **11** (126 MHz, D_2O).

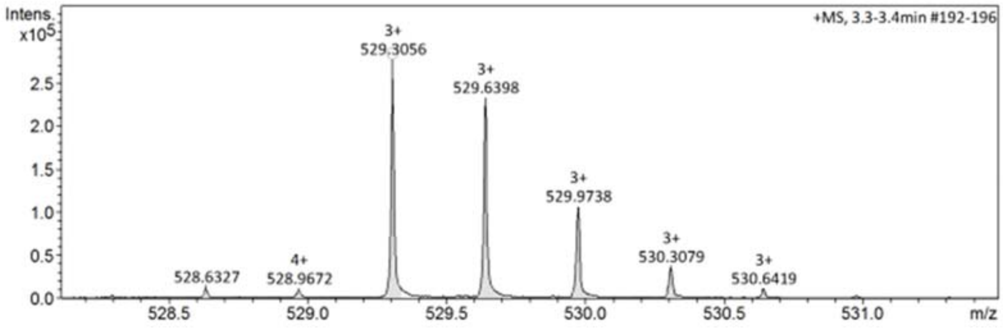

Figure S67. HR-ESI (ESI$^+$ Q-TOF) of compound **11**.

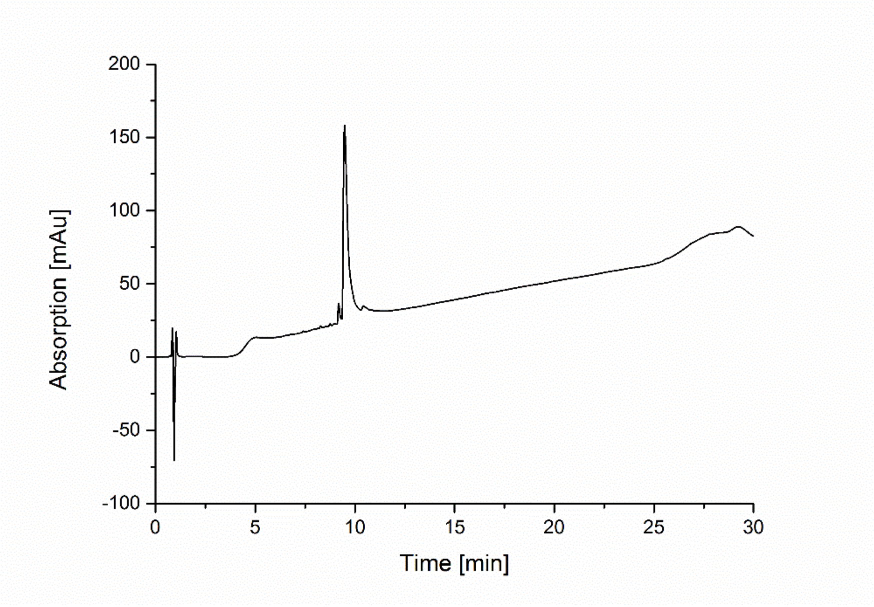

Figure S68. RP-HPLC chromatogram of compound **11** (gradient from 0% to 50% eluent B over 30 min at 25°C)

S5 Determination of SCP Functionalization Degrees

Oligomer Functionalization of PEG-CA-SCPs

For the functionalization of PEG-CA-SCPs with oligomers 1 mL of SCP dispersion is washed with 2-(*N*-morpholino)ethansulfonic acid (MES) buffer with a concentration of 0.1 mol L^{-1} with pH 5 via centrifugation (13500 rpm, 5 min). Afterwards, 200 µL of MES buffer is added to the particles. Additionally, 500 µL of Oligomer in MES buffer is added. The amount of Oligomer was equal to a 10 fold excess in comparison to carboxylic acid groups on the particles (see Table S1). To start the reaction 100 µL of a solution of 1-Ethyl-3-(3-dimethylaminopropyl)carbodiimide hydrochloride (EDC) with concentration 100 mg mL^{-1} in ultrapure water is added. The reaction solution is shaken for 2 h before the reaction solution was removed via centrifugation (13500 rpm, 5 min) and replaced with a fresh reaction solution. After an additional reaction time of 2 h the supernatant is removed and the particle

are washed with ultrapure water via centrifugation (13500 rpm, 5 min). The functionalization degree was determined via microscope based TBO titration.

Table S1: Overview over molecular weight and amount of the oligomers that were used per reaction step for PEG-CA-SCP functionalization and the functionalization degree of the particles determined via microscope based TBO titration.

**Oligomer**	MW [g/mol]	Amount of oligomer per reaction step [mg]	Functionalization degree [%]
1N3N (4)	1650	3.0	88
1C3C (3)	1636	2.9	98
1D3D (5)	1622	2.9	84
1D3C (6)	1584	2.8	86
1N2C (11)	1584	2.8	87
1C3N (10)	1584	2.8	98
1D3C (7)	1570	2.8	98
1C3D (9)	1570	2.8	98
1N3D (8)	1636	2.9	98

Crotonic Acid Titration via UV-VIS Spectroscopy

The determination of carboxylic acid groups on the PEG-CA particles was done in triplicates. 1 mL of SCP dispersion was dried after exchanging the water with ethanol via centrifugation (13500 rpm, 5 min) to determine the amount of particles. To the dried particles 1 mL of TBO (toluidine blue O) solution with a concentration of 0.3125 mmol L^{-1} with a pH of 10-11 was added wrapped in aluminum foil and shaken overnight. After that the solution was centrifuged (13500 rpm, 5 min) and 0.3 mL were taken and diluted to 2 mL with sodium hydroxide solution with pH 10-11. The same procedure was done with a blank where no particles were added in the beginning. The absorption of this solution was measured via UV-VIS spectroscopy and the absorption at 633 nm was used to calculate the functionalization degree using the following equation:

$$D_{CGF} = N_R(1 - A_S/A_E)/W_{Dry}$$

Where D_{CGF} is the carboxylic acid functionalization degree, A_S and A_R are the UV-VIS absorbances of sample and reference, W_{Dry} is the dry weight of 1.0 mL SCPs, N_R is the amount of TBO in the reference in units of µmol.

Determination of oligomer functionalization degree via microscope based TBO titration

For the determination of functionalization degree of oligomer functionalized SCPs 100 µL of SCP solution was washed via centrifugation (13500 rpm, 5 min) with sodium hydroxide solution pH 10-11. After removing the supernatant 125 µL of TBO solution with 0.3125 mmol L^{-1} were added, wrapped in aluminum foil and shaken overnight. Next, the TBO solution was removed and the particles were washed three times with 1 mL of sodium hydroxide solution with pH 10-11 and afterwards dissolved

in 125 µL. The same procedure was done for PEG-CA particles and non-functionalized PEG particles. Next, for all particle solutions the grey value was determined for 20 particles per batch to calculate the functionalization degree as following:

$$D_{OGF} = (1 - (G_N - G_{SCP})/\Delta G_B) * 100$$

Where D_{OGF} is the oligomer functionalization degree, ΔG_B is the difference of grey values between non-functionalized and carboxylic acid functionalized SCPs ($\Delta G_B > 0$), G_N is the average grey value of non-functionalized SCPs and G_{SCP} is the average grey value of oligomer functionalized SCPs.

S6 Determination of the SCPs elastic modulus

Force-indentation measurement with a NanoWizard 2 AFM provided the elastic modulus of the SCPs. A silica bead with a raduis of 2.3 µm was glued with an epoxy glue onto a tipless, non-coated cantilever (spring constant 0.32 N/m; NanoAndMore GmbH). Several force curves were recorded from different particles and analyzed with the novel contact model developed by Glaubitz et al.[5]. The model considers deformation of the object at two sites: the indentation site of the AFM probe and at the contact with the solid support. The respective deformation (*δ*) –force (*F*) dependence reads:

$$\delta(F) = \left(\frac{3F}{4E} \cdot \frac{1-\upsilon^2}{R_{AFM}^{\frac{1}{2}}}\right)^{\frac{2}{3}} + \left[\frac{3(1-\upsilon^2)\left(F + 6W\pi R_{SCP} + \sqrt{12W\pi R_{SCP} F_c (6W\pi R_{SCP})^2}\right)}{4E \cdot R_{SCP}^{\frac{1}{2}}}\right]^{\frac{2}{3}} - \left[\frac{9W\pi(1-\upsilon^2)}{E}\right]^{\frac{2}{3}} \cdot R_{SCP}^{\frac{1}{3}}$$

where *E* is the elastic modulus of the indented SCP, R_{SCP} its radius, *υ* the Poisson ratio of the SCP, *W* the SCP adhesion energy with the support surface and R_{AFM} the radius of the indenter. The Poisson ration was assumed to be 0.5 (volume conservation upon indentation). *E* and *W* were free fit parameters. The elastic moduli of FN SCPs were on the order of 72 kPa and their surface energy varied only marginally between 20 and 30 µJ/m^2 for the different fits.

For all SCPs except for the diamine oligomer (**4**) carrying SCPs the elastic moduli were similar, around 71.9 ± 10.5 kPa. The elastic modulus for the diamine oligomer (**4**) functionalized SCPs was 103 ± 14.4 kPa. The increase in elastic modulus for the diamine carrying SCP is probably the to an extended conformation of the of the oligomer stiffening the PEG network. But overall, the rather low variations of the elastic moduli for the different SCPs are expected due to the low density of oligomers in the SCP. About 13.5-14.2 wt% of the SCPs material are oligomers. Due to the high SCP swelling degree the oligomer concentration within the SCP network is 11 mmol l^{-1}.

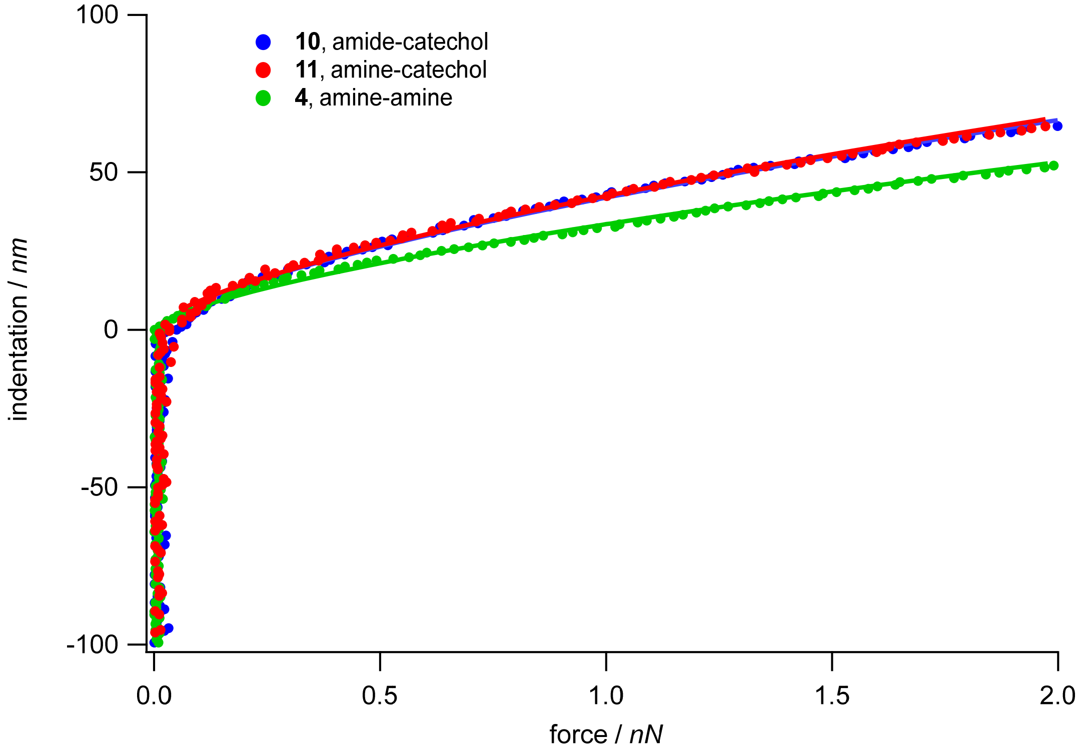

Figure S69. Typical AFM indentation-force curves for the analysis with the contact model developed by Glaubitz et al.[5] The solid lines are fits to the data.

S7 Reflection Interference Contrast Microscopy (RICM) measurements Setup

RICM on an inverted microscope (Olympus IX73) was used to obtain the contact area between the microparticles and a hard glass surface. For illumination a monochromatic (530 nm) collimated LED (Thorlabs, Germany, M530L2-C1) was used. An UPlanFL N 60x/0.90 dry objective (Olympus Corporation, Japan), additional polarizers and a quarter waveplate (Thorlabs, germany) to avoid internal reflections and a monochrome CMOS camera (DMK 33UX174, The Imaging Source Europe GmbH, Germany) were used to image the RICM patterns.

Determination of the Contact Radius

RICM was used to measure the contact radius formed by the SCPs resting on the polymer surface (Figure S2). Polarized light waves reflected from the upper glass surface (I_1) and the surface of the bead (I_2) interact to create an interference image. The intensity at a given position in the image depends on the separation $h(x)$ between the two surfaces: $I(x) = I_1 + I_2 + 2\cdot\mathrm{sqrt}(I_1 \cdot I_2)\cos[2k\cdot h(x) + \pi]$, where $k = 2\pi n/\lambda$, and n and λ are the index of refraction of water and the wavelength of the monochromatic light, respectively. In order to detect the interference pattern, stray light was reduced by an ‘antiflex’ technique. This is accomplished by crossed polarizer and analyzer filter with a λ/4-plate placed between the objective lens and the analyzer.[6]

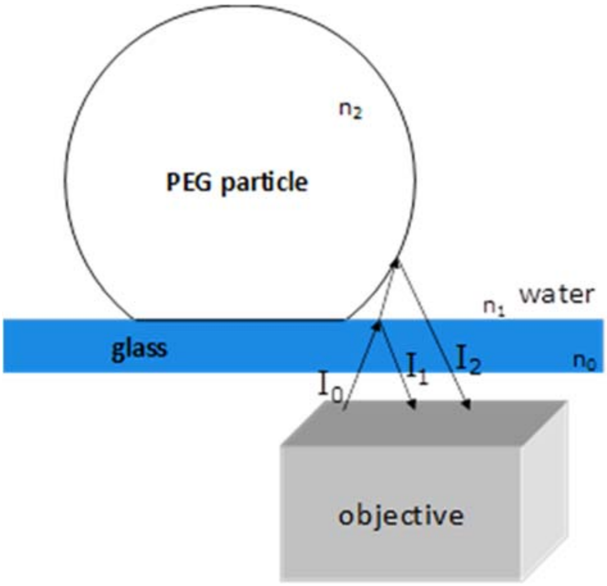

Figure S70. Schematic drawing of the RICM principle.

Correction Factors

For analysis of the RICM patterns correction factors must be determined for finite aperture and geometry effects. To obtain the correction factors, we imaged hard, non-deformable glass beads on a glass surface in RICM mode with a known size and curvature. We recorded 5 glass beads with a diameter in the range of 20-40 µm (polysciences) and extracted the intensity profile. Using the profiles, we reconstructed the shape of the beads and compared it to the known spherical shapes of the glass beads (glass bead radius *R* measured by light microscope), and determined the correction factors, see Pussak et al.[7]

Contact radius determination

To determine the contact radius *a* of the SCP on the polymer surface we reconstructed the height profile of the particles from the RICM images (see Figure S3). This was done by determining the lateral $x(i)$ positions of the *i*-th minima and maxima by a self-written IgorPro procedure (Wavemetrics, USA). Next, the vertical position $y(i)$ of the maxima and minima were determined by

$$y(i) = \frac{i\lambda}{4n} + c_i ,$$

where *n* is the refractive index and λ the wavelength. The height profile was then reconstructed by plotting $y(i)$ vs $x(i)$ and fitting the data by a circle equation representing the assumed shape of the SCP:

$$y(x) = y_0 + \sqrt{R^2 - x^2} .$$

where *R* is the independently measured SCP radius and y_0 the vertical shift of the SCP center due to flattening of the SCP upon adhesion. The fit with y_0 as the only free fit parameter intersects with the x-axis and gives the contact radius *a*.

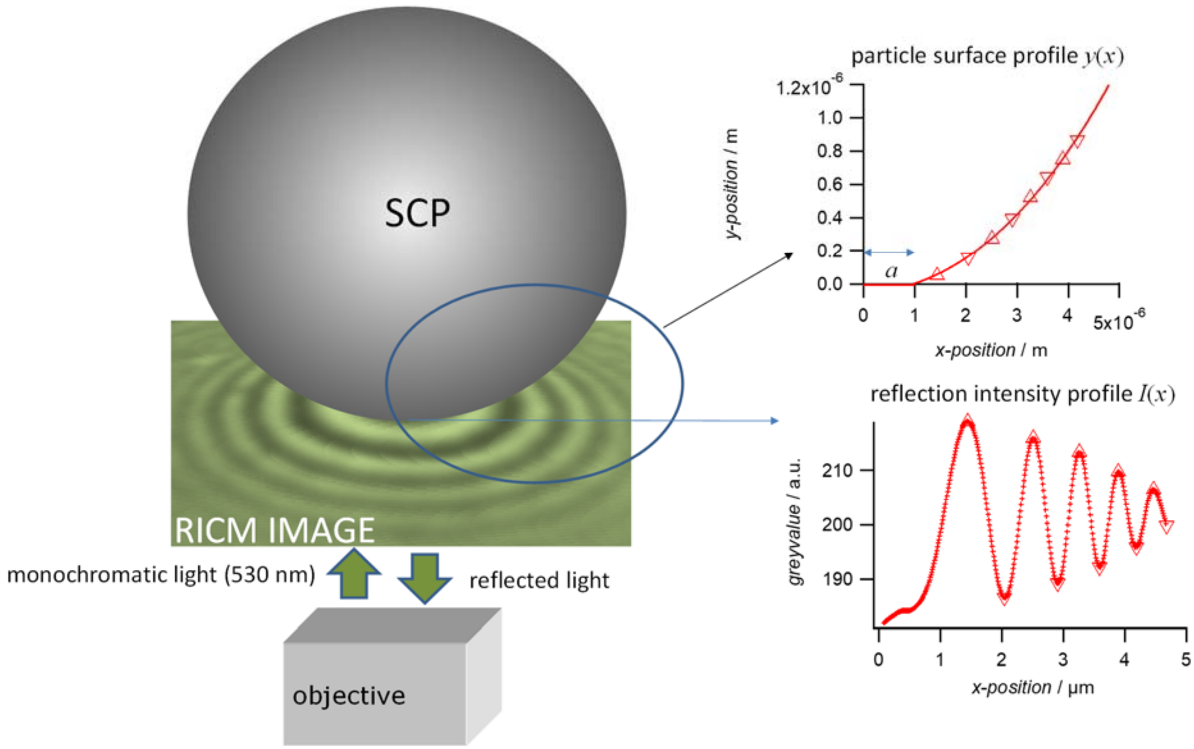

Figure S71. Left: schematic representation of the measurement setup. Bottom right: actual intensity profile of an adherent SCP showing 5 minima and 5 maxima. Top right: reconstructed surface profile of the SCP and the contact radius *a* at the intersection of the profile at $y = 0$.

S8 Stability of the catechol group

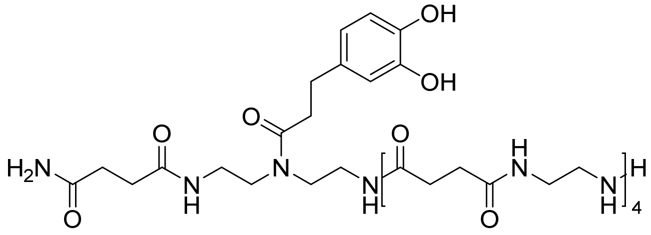

12

Compound **12** was used as a model for the investigation of the catechol stability. For this 1 mg was dissolved in 500 µl water and the mixture was measured via RP-HPLC directly after dissolving and after 12 days.

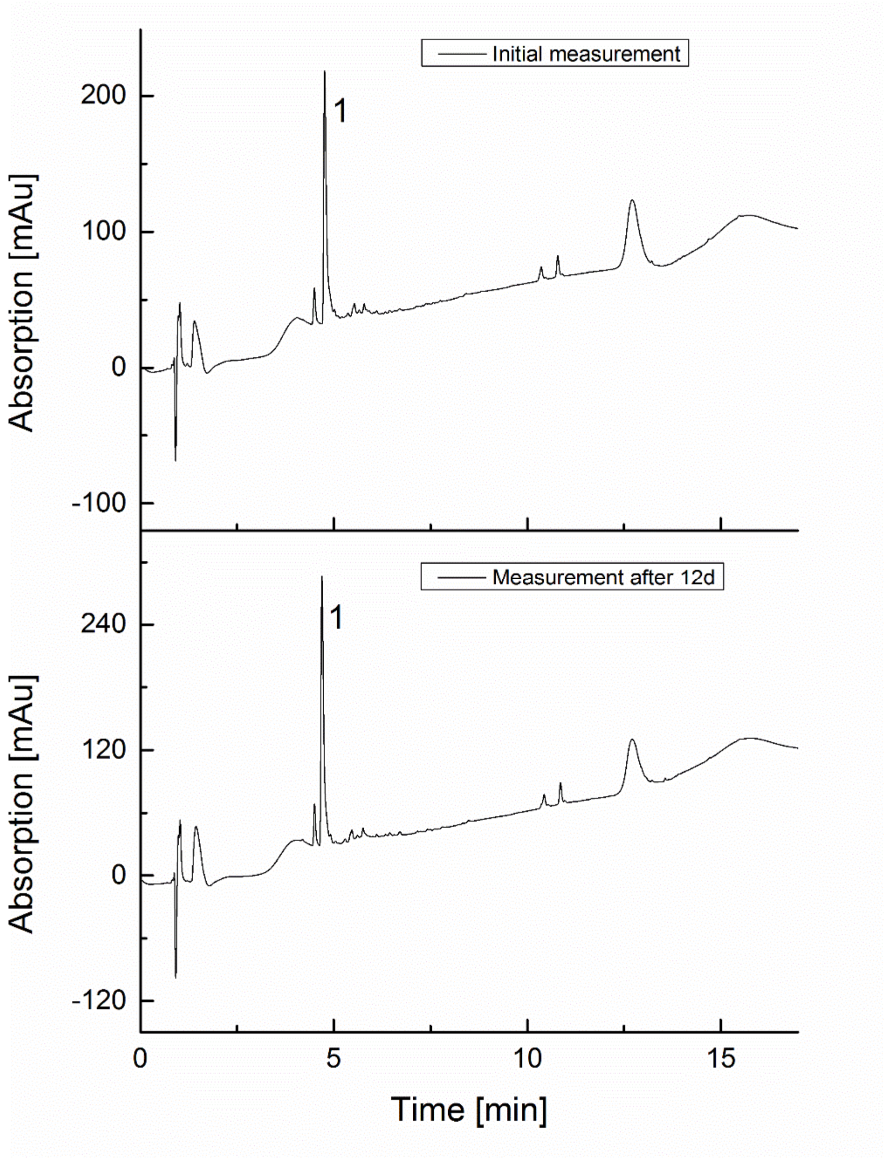

Figure S72. RP-HPLC of compound **12** directly after dissolving and after 12 days. Peak 1 shows compound **12**.

S9 Non-normalized and oligomer concentration normalized adhesion energy values

Supporting Information

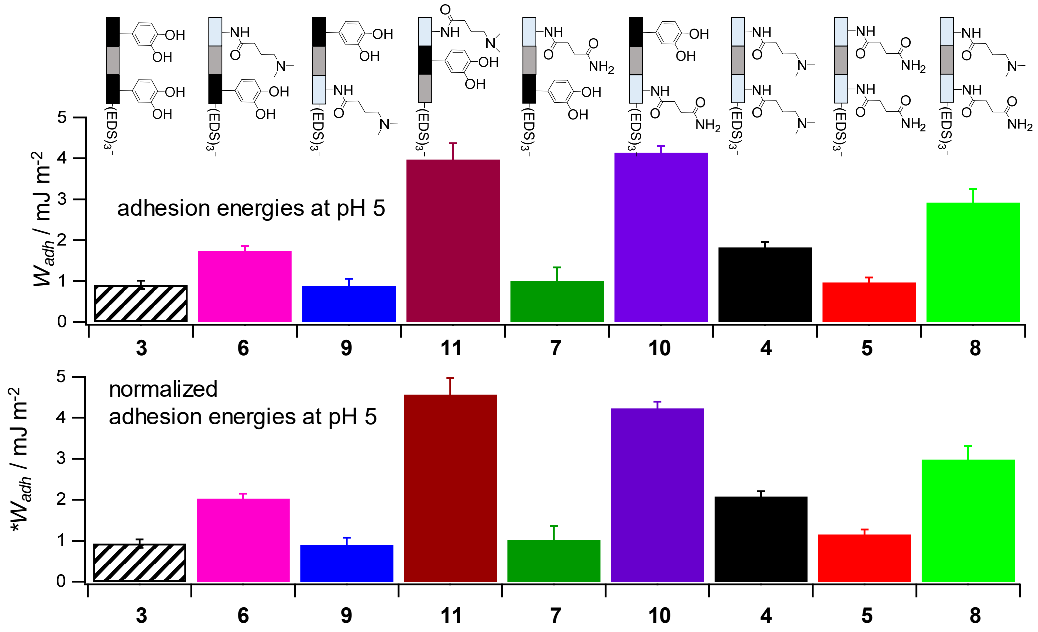

Figure S73. Comparison between non-normalized and normalized adhesion values. Top: non-normalized adhesion values (W_{adh}). Bottom: oligomer concentration normalized adhesion energy values (W_{adh}).

Supporting References

[1] C. Gerke, M. F. Ebbesen, D. Jansen, S. Boden, T. Freichel, L. Hartmann, *Biomacromolecules* **2017**, *18*, 787-796.

[2] D. Pussak, M. Behra, S. Schmidt, L. Hartmann, *Soft Matter* **2012**, *8*, 1664-1672.

[3] S. Schmidt, H. Wang, D. Pussak, S. Mosca, L. Hartmann, *Beilstein Journal of Organic Chemistry* **2015**, *11*, 720-729.

[4] D. Ponader, F. Wojcik, F. Beceren-Braun, J. Dernedde, L. Hartmann, *Biomacromolecules* **2012**, *13*, 1845-1852.

[5] M. Glaubitz, N. Medvedev, D. Pussak, L. Hartmann, s. schmidt, C. A. Helm, M. Delcea, *Soft Matter* **2014**.

[6] L. Limozin, K. Sengupta, *ChemPhysChem* **2009**, *10*, 2752-2768.

[7] D. Pussak, D. Ponader, S. Mosca, S. V. Ruiz, L. Hartmann, S. Schmidt, *Angewandte Chemie International Edition* **2013**, *52*, 6084-6087.
